# Effect of leukotriene receptor antagonist use on the future risk of Parkinson’s disease in older patients with asthma

**DOI:** 10.1093/braincomms/fcaf340

**Published:** 2025-09-10

**Authors:** Chengsheng Ju, Boqing Chen, Anette Schrag, Camille Carroll, Thomas Foltynie, Li Wei

**Affiliations:** Research Department of Practice and Policy, University College London School of Pharmacy, London WC1N 1AX, United Kingdom; Research Department of Practice and Policy, University College London School of Pharmacy, London WC1N 1AX, United Kingdom; Department of Clinical and Movement Neurosciences, University College London Institute of Neurology, London WC1N 3BG, United Kingdom; Translational and Clinical Research Institute, Newcastle University, Newcastle-upon-Tyne, NE1 7RU, United Kingdom; Department of Clinical and Movement Neurosciences, University College London Institute of Neurology, London WC1N 3BG, United Kingdom; Research Department of Practice and Policy, University College London School of Pharmacy, London WC1N 1AX, United Kingdom; Laboratory of Data Discovery for Health (D24H), Hong Kong Science Park, Hong Kong Special Administrative Region, China; Centre for Medicines Optimisation Research and Education, University College London Hospitals National Health Service (NHS) Foundation Trust, London NW1 2PG, United Kingdom

**Keywords:** Parkinson’s disease, leukotriene receptor antagonist, target trial emulation, asthma

## Abstract

Current treatments for Parkinson’s disease focus on symptom management, with no therapies yet demonstrated to slow disease progression. Leukotriene receptor antagonists, widely used for asthma, have shown potential neuroprotective effects for Parkinson’s disease in preclinical studies, but have also been associated with an elevated risk of neuropsychiatric events and sleep disorders. We assessed the effect of leukotriene receptor antagonist treatment on the risk of Parkinson’s disease, neuropsychiatric events, and sleep disorders in patients with asthma aged over 50 years. We conducted a cohort study using the UK Clinical Practice Research Datalink between January 2000 and December 2020. The study emulated sequential target trials (*n* = 140) using observational data, comparing leukotriene receptor antagonist treatment to no leukotriene receptor antagonist treatment among patients aged 50–84 years with asthma. The primary outcome was the risk of incident Parkinson’s disease, and the secondary outcomes were neuropsychiatric events (anxiety, depression, and psychosis), and sleep disorders. Propensity score matching was employed to minimize confounding. We used pooled logistic regression models to calculate risk ratios as observational analogues of intention-to-treat and per protocol effects. A total of 97 049 matched pairs were included in the analysis, with 573 Parkinson’s disease cases observed in the leukotriene receptor antagonist group and 537 in the nonleukotriene receptor antagonist group over a median follow-up of 5.9 years and 5.7 years, respectively. No significant difference in Parkinson’s disease risk was observed between the two groups in either the intention-to-treat analysis [10-year risk ratio: 1.09; 95% confidence interval (CI), 0.94–1.26] or the per protocol analysis (10-year risk ratio: 0.95; 95% CI, 0.75–1.16). However, there was a higher risk of depression (intention-to-treat effect: 10-year risk ratio: 1.12; 95% CI, 1.07–1.16; number-needed-to-harm = 93; per protocol effect: 10-year risk ratio: 1.15; 95% CI, 1.08–1.22; number-needed-to-harm = 75) and sleep disorders (intention-to-treat effect: 10-year risk ratio: 1.14; 95% CI, 1.11–1.19; number-needed-to-harm = 77; per protocol effect: 10-year risk ratio: 1.12; 95% CI, 1.06–1.19; number-needed-to-harm = 88) with leukotriene receptor antagonist treatment. No clear effect was observed for anxiety or psychosis. Leukotriene receptor antagonist treatment was not associated with an altered risk of Parkinson’s disease among people aged 50–84 years with asthma but was linked to a higher incidence of neuropsychiatric events.

## Teaser

Ju *et al.* reported that leukotriene receptor antagonist treatment was not associated with altered risk of Parkinson’s disease in patients aged 50–84 years with asthma. However, the treatment was linked to higher risks of depression and sleep disorders, highlighting the need for monitoring neuropsychiatric outcomes in this population.

## Graphical Abstract

**Graphical Abstract:**
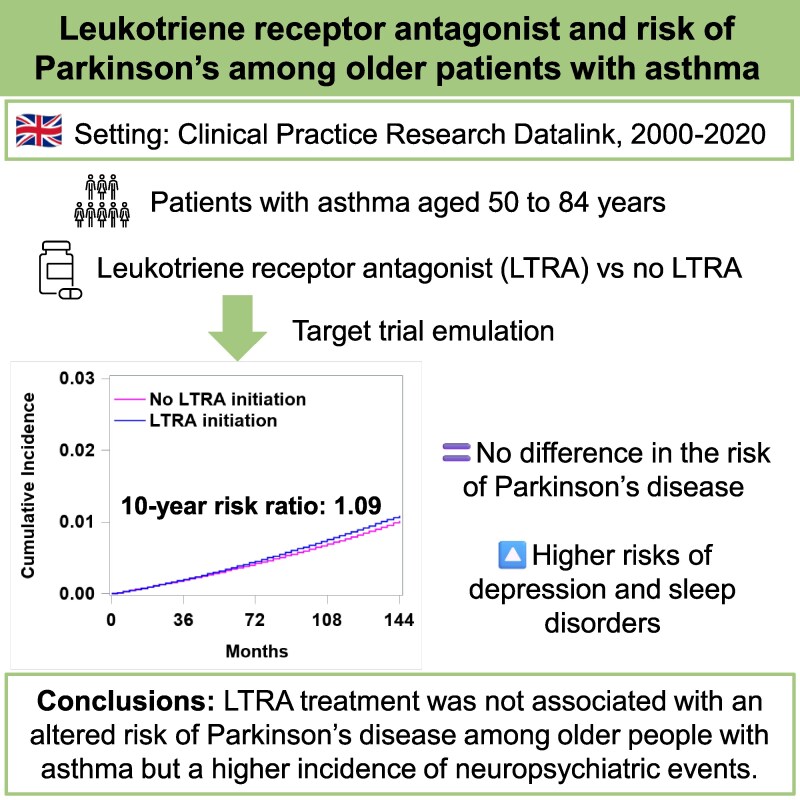

## Background

Parkinson’s disease affects approximately 145 000 people in the UK, predicted to increase to 172 000 by 2030.^1^ Globally, this number increased from 2.5 million in 1990 to 6.1 million in 2016.^2^ Symptoms progress relentlessly, resulting in impaired mobility, falls, dysautonomia, cognitive impairment, and mental health decline. This results in escalating care needs and an economic impact of >£20 000 per annum per Parkinson’s disease household.^3^ Current treatment is limited to symptomatic management with no interventions that slow progression. The identification of disease-modifying agents for Parkinson’s disease remains a major research priority.^4^

Leukotriene receptor antagonists (LTRA) are safe and well-tolerated drugs that are widely used in asthma and allergic rhinitis, with montelukast being the most commonly prescribed of the LTRA in the UK.^5^ Numerous preclinical studies have suggested a neuroprotective effect of LTRA on dopaminergic neurons, possibly via reduced microglial activation and attenuated oxidative stress.^6-8^ The clinical relevance of LTRA on the risk of progression of Parkinson’s disease still needs to be teased out by trials and observational studies, which are currently lacking. To date, there is only one observational study available, suggesting a lower risk of Parkinson’s disease associated with higher cumulative doses of montelukast,^9^ while another pharmacovigilance study has suggested LTRA treatment is associated with an increased use of anti-parkinsonian drugs.^10^ A recent pilot study evaluating short-term, high-dose montelukast in patients with Parkinson’s disease showed the treatment is well tolerated and safe in this population.^11^

However, the Medicines and Healthcare products Regulatory Agency (MHRA) has issued warnings about neuropsychiatric side effects associated with montelukast, including sleep disturbance, agitation, and depression.^12^ These neuropsychiatric events are of particular concern in the context of Parkinson’s disease, as they commonly occur not only in established Parkinson’s disease but also the prodromal phase.^13^ Although the recent study reported no psychiatric adverse effects, it was a small, open-label pilot study with no control group and a short follow-up duration, limiting the generalizability of its findings.^11^ The occurrence of long-term neuropsychiatric effects of LTRA would therefore be of major concern if the drug were to be repurposed for modifying the risk or rate of progression of Parkinson’s disease.

The current study aimed to provide robust real-world evidence on the effectiveness of LTRA on the future risk of developing Parkinson’s as well as the risks of neuropsychiatric events to help consider the net impact of the use of these drugs. We used existing longitudinal data to explicitly emulate a sequence of randomized controlled trials of LTRA, to minimize the risk of bias in all analyses.

## Materials and methods

### Ethics approval

This study was approved by the Clinical Practice Research Datalink (CPRD) Research Data Governance (RDG) process; protocol number: 23_003508.

#### Data sources

This population based cohort study was conducted using data from the UK CPRD GOLD and Aurum databases, linked with the Hospital Episode Statistics Admitted Patient Care (HES APC) and the Office for National Statistics (ONS) UK databases.^14^ CPRD covers data from more than 60 million cumulative patients since it started in 1987 from approximately 2000 general practices in the UK.^15^ CPRD contains longitudinal information on primary care diagnoses, prescriptions, sociodemographic characteristics, personal information, and laboratory test results. Linkage from CPRD to HES in-patient data was available for about 90% of participating general practices in England.^16^ HES APC contains information on in-hospital diagnoses, procedures, and admission and discharge dates. ONS contains information on each person’s vital status.

#### Study design

This study was a cohort study emulating a target pragmatic trial using observational data. We first designed a hypothetical target trial aiming to evaluate the effect of LTRA on the risk of Parkinson’s disease, and then emulated the trial with observational data from the CPRD database. The specification for the protocol of the hypothetical target trial we wished to emulate, and the actual trial emulation using observational data, are summarized in Supplementary Table 1.

#### Eligibility criteria

Eligible patients for this study were those who had a history of asthma diagnosis aged between 50 and 84 years between January 2000 and December 2020 and had linked data from HES and ONS. We excluded any patients at baseline with a diagnosis of Parkinson’s disease or secondary Parkinsonism, any prescription of dopaminergic anti-parkinsonian treatment, or prior LTRA treatment within the preceding 365 days.

#### Treatment strategies and follow-up

The treatment strategies under comparison were as follows: (i) initiation and continuous use of LTRA versus (ii) no initiation of LTRA during follow-up.

We emulated a sequence of trials evaluating the treatment effect of LTRA in patients with asthma aged between 50 and 84 years using 3-month time intervals to define individuals into age defined groups and to assign the treatment strategies. Specifically, for the first trial, we identified all patients who turned 50 and met the eligibility criteria at that exact age (index date) and assigned the treatment strategies at the index date. If they initiated LTRA treatment within the next 3 months, they were classified as LTRA initiators, and vice versa. All patients were followed from the index date until the occurrence of study outcome, transfer-out from the registered practice, end of data collection from that general practitioner (GP) practice, whichever occurred first. Follow-up time was calculated separately for each study outcome.

Next, we emulated the second trial 3 months after the first trial, and identified all eligible patients who turned 50 years and 3 months, and used the same criteria to assign the treatment strategies to them on this day. We then repeated this process for all patients aged 50–84 years for every 3-month age interval. In total, 140 trials were emulated between the ages of 50 and 84 years old in 3-month subgroups. During this process, one patient can be included in multiple trials if they meet the eligibility criteria at multiple time points.

#### Outcomes

The primary outcome was a new diagnosis of Parkinson’s disease, which was defined as having a GP record or a hospital admission including a code of Parkinson’s disease and at least two prescriptions of anti-parkinsonian drugs (levodopa, amantadine, dopamine receptor agonists, catechol-O-methyl transferase inhibitors, or monoamine-oxidase-B inhibitors).

We also used alternative definitions for Parkinson’s disease as following:

1. Having a GP record of Parkinson’s disease (read codes or SNOMED-CT codes); or
2. Having a hospital admission including a code of Parkinson’s disease (ICD-10 codes); or
3. Having a GP record or a hospital admission including a code of Parkinson’s disease.

The secondary outcomes were the following:

1. Neuropsychiatric events (including anxiety, depression, and psychosis) or sleep disorders (insomnia, hypersomnia, parasomnia, circadian rhythm sleep–wake disorders, and nonspecific). We excluded patients with a history at baseline of each respective neuropsychiatric/sleep disorder event in the analyses of the neuropsychiatric outcomes.
2. All-cause mortality and a composite outcome of Parkinson’s disease and mortality. We also conducted an alternative analysis on all-cause mortality alone and a composite outcome of mortality and Parkinson’s disease, which allowed us to better examine the impact of competing risk on our findings. Additionally, the analysis of mortality can be used to assess whether severity of asthma (the perceived reason for LTRA initiation in the real-world) has confounded our results.^17^ We considered mortality occurring before a Parkinson’s disease diagnosis as a competing risk event for Parkinson’s disease. In the primary analysis, we considered mortality as a censoring event, on which our target causal estimate has a controlled direct effect—which should be of interest to our question on drug repurposing but comes with unverifiable assumptions.^18^

All diagnostic codes for the study outcomes are provided in Supplementary Table 2.

#### Covariates

The covariates measured at baseline included age (not included in the propensity score model, as it was used for exact matching in the sequential trial emulation), sex, duration of asthma, number of hospitalisations in the past year, number of asthma-related hospitalisations in the past year, calendar year of cohort entry (2000–20), Index of Multiple Deprivation (IMD) score for socioeconomic status (1–5), smoking status (current smoker, ex-smoker, and nonsmoker), body mass index (BMI) categories (underweight, normal weight, overweight, and obese), eosinophilia in the past year, frailty measured by electronic frailty index^19^ (fit, mild frailty, moderate frailty, and severe frailty), comorbidity history: allergic rhinitis, atopic dermatitis, alcohol-related disorder, cancer, chronic kidney disease, chronic obstructive pulmonary disease, dementia, diabetes, gout, liver disease, hypertension, myocardial infarction, stroke, depression, sleep disorder, epilepsy, psychosis, head injury, fall, fracture, lower respiratory tract infection, and influenza infection; concurrent medications in past 180 days: short-acting beta-agonists, long-acting beta-agonists, muscarinic antagonists, inhaled corticosteroids, oral corticosteroids, xanthine-derived bronchodilators, antihistamines, low-dose aspirin, calcium-channel blockers, statins, anxiolytics and sedatives, antidepressants, antipsychotics, metformin, incretin-based antidiabetic drugs, and insulin. The diagnoses of all comorbidities were identified using validated algorithms available on the CALIBER platform.^20^

#### Trial emulation and statistical analyses

We emulated a series of sequential target trials at 3-month intervals, defining trial entry based on age, from 50 years to 84 years old. In each trial, we updated the LTRA treatment, baseline covariates, and outcome status for each patient, and the LTRA treatment and outcome were further updated at each 3-month interval during follow-up. This method has been used in previous target trial emulation to increase statistical efficiency in analysis.^21^

To estimate the intention-to-treat effect, the baseline confounding was adjusted using propensity score matching to achieve baseline exchangeability between patient cohorts. The propensity score (defined as the probability of initiating LTRA treatment, which is conditional on the baseline characteristics for each patient) was estimated using a logistic regression model based on all measured covariates except for age, and all continuous variables were included in the linear and quadratic terms. Patients who initiated LTRA in the first 3 months were matched to patients who did not initiate LTRA based on the propensity score using a ‘greedy nearest-neighbour’ method with a caliper of 0.05.^22^

To estimate the per protocol effect, we fitted the same pooled logistic models as the intention-to-treat analysis, but replaced the treatment arm indicator with a time-varying function of LTRA prescription since baseline. This approach allowed us to model the dose–response relationship, capturing the effect of sustained LTRA treatment over time. The function included both linear and quadratic terms to account for potential nonlinearity in the dose–response.^23^ Using this model, we estimated the effect of continuous LTRA use (‘always treated,’ where the cumulative exposed time equalled time since baseline) versus no treatment (‘never treated,’ where the cumulative exposed time remained zero throughout).

We estimated the cumulative incidence in the matched cohort for the intention-to-treat and per protocol effect. We fitted a pooled logistic model including an indicator for treatment, month and its quadratic term, and an interaction term between treatment and time (to allow for nonproportional hazards). The predicted probabilities from this logistic model were used to estimate the adjusted absolute risks and risk ratios (RRs) for each study outcome at each interval. We used nonparametric bootstrapping with 300 individual-level resamplings within matched pairs to compute the 95% CIs for the absolute risks and RRs. This approach has been shown to yield accurate variance estimates in propensity score matched samples^24^ and is widely applied in sequential trial emulations.^25,26^

All data were summarised as medians with interquartile ranges (IQR) for continuous variables and numbers of subjects (%) for categorical variables. Standardised mean differences (SMD) were used to evaluate the differences in baseline variables between groups. An SMD lower than 0.1 was considered as good balance between groups. Findings were considered to be statistically significant when the 95% CIs for risk on a relative scale did not cross 1 or when the 95% CI for risk difference on an absolute scale did not cross zero. For the subgroup analyses, *P*-values smaller than 0.05 were considered statistically significant. Number-needed-to-treat (NNT) or number-needed-to-harm (NNH) was derived from the adjusted absolute risks and their 95% CIs for any statistically significant findings. All statistical analyses were performed with SAS software, version 9.4 (SAS Institute).

#### Sensitivity analysis and exploratory analysis

In the main analysis, we allowed patients who reinitiated LTRA after 365 days following LTRA treatment discontinuation to be reincluded as LTRA initiators in subsequent trials; as a first sensitivity analysis, we excluded these patients and their matched pairs. Second, we conducted sequential trial emulation based on calendar time, with 84 trials initiated at 3-month intervals from January 2000 to December 2020. This approach ensures that findings are consistent across different time periods and accounts for temporal trends in prescribing patterns and disease incidence. Third, we excluded further patients with less than 5-year record history in the database to ensure adequate baseline covariate capture and reduce potential bias from incomplete medical histories. Fourth, we applied a 3-year lead time by defining the date of Parkinson’s disease onset at 3 years before the date of Parkinson’s disease diagnosis. This is to account for the long latency period of Parkinson’s disease. Fifth, we performed exploratory outcomes of autonomic and sensory presentations associated with Parkinson’s disease, including anosmia, constipation, dizziness, hearing loss, and urinary incontinence.^13^ This analysis provides additional insight into potential early manifestations of Parkinson’s disease in relation to LTRA treatment. Lastly, we explored age-based (≥65 years or <65 years) and sex-based (male or female) differences on the risks of Parkinson’s disease.

## Results

**Figure 1:**
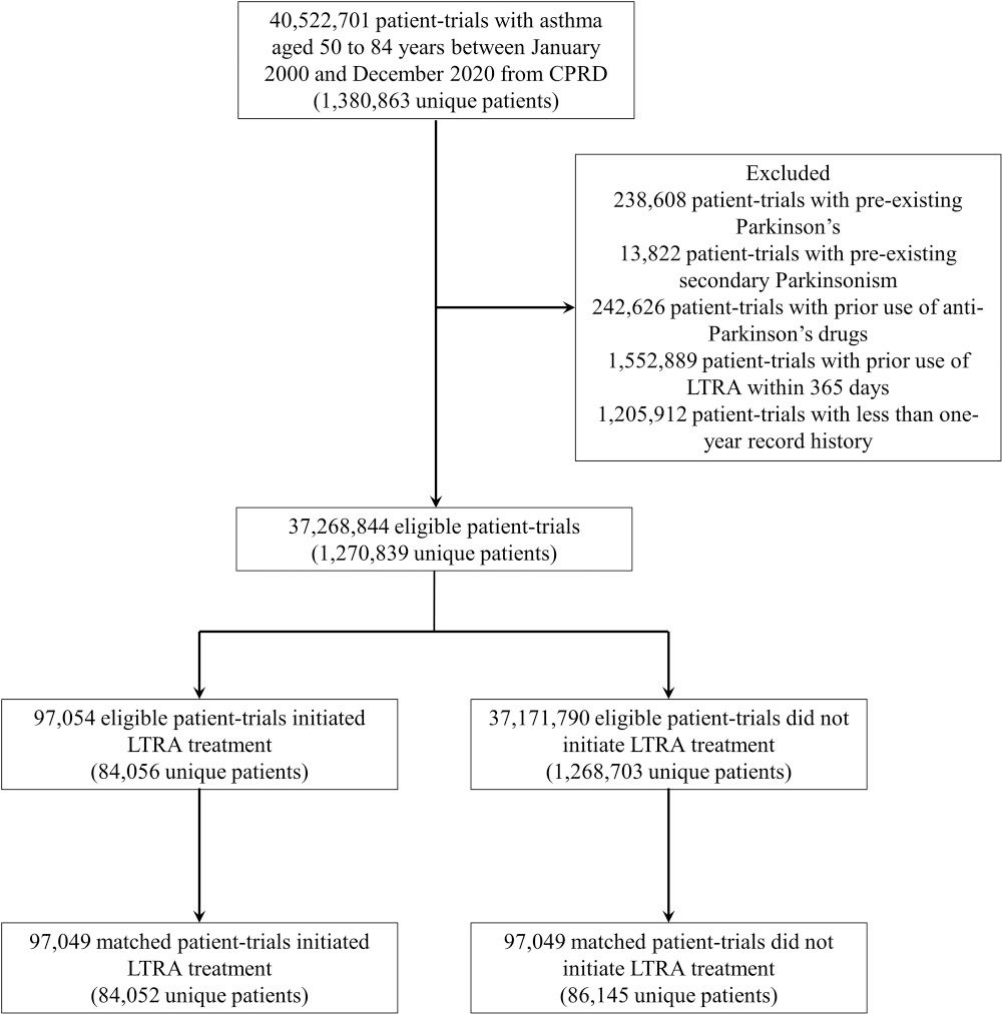
**Selection of patients from clinical practice research datalink (CPRD) for trial emulation.** A total of 1 270 839 unique patients from the CPRD met the eligibility criteria. Under the sequential trial design, these patients contributed to 140 nested trials, resulting in 37 268 844 patient-trials. Of these, 97 054 patient-trials were eligible for the leukotriene receptor antagonist (LTRA) initiation arm and 37 171 790 for the no LTRA initiation arm. After propensity score matching, 97 049 patient-trials initiating LTRA were matched to 97 049 patient-trials not initiating LTRA. Numbers in parentheses represent unique individuals in each group. The number of LTRA initiators and noninitiators does not sum to the total number of eligible individuals because some contributed to both arms across different emulated trials.

There were 1 270 839 unique patients with a history of asthma diagnosis aged 50–84 years from CPRD who met the eligibility criteria and were included in the analysis. With the sequential trial definition, these patients participated in 140 trials 37 268 844 times (37 268 844 patient-trials). Among them, there were 97 054 patient-trials compatible with ‘initiating LTRA treatment’ arm and 37 171 790 patient-trials were compatible with ‘no LTRA treatment’ arm. After propensity score matching, there were 97 049 patient-trials on LTRA initiation matched to 97 049 patient-trials on no LTRA initiation. Figure 1 shows a flowchart of participant selection, and the description of the number of eligible patients, number of Parkinson’s disease cases, and mean follow-up in each emulated trial before and after matching (shown in Supplementary Table 3). Table 1 shows the selected baseline characteristics of pooled individuals from all emulated trials before and after matching, and the full description of the baseline characteristics can be found in Supplementary Table 4. All covariates are balanced in the matched cohort. The median age of the included participants was 62.5 [IQR, 56.0–70.3] years and 63.7% of participants were women.

**Table 1:** Selected baseline characteristics of patients who initiated LTRA treatment and did not initiate LTRA treatment pooling all emulated trials, before and after matching (pooled emulated trials)

	Before matching			After matching		
	LTRA	No LTRA	SMD	LTRA	No LTRA	SMD
	*N* = 97 054	*N* = 37 171 790		*N* = 97 049	*N* = 97 049	
Age, years [IQR]	62.5 [56–70.3]	63.8 [56.3–72.5]	−0.124	62.5 [56–70.3]	62.5 [56–70.3]	0
Female sex, *n* (%)	61 853 (63.7)	21 327 141 (57.4)	−0.130	61 849 (63.7)	61 834 (63.7)	0
Duration since asthma diagnosis, years [IQR]	13.7 [6.2–23.8]	12.6 [6.2–21.2]	0.093	13.7 [6.2–23.8]	14.0 [7.0–23.2]	−0.002
IMD quintiles, *n* (%)						
1 (most deprived)	20 095 (20.7)	8 130 533 (21.9)	−0.029	20 095 (20.7)	20 017 (20.6)	0.002
2	20 533 (21.2)	7 870 390 (21.2)	0	20 532 (21.2)	20 559 (21.2)	−0.001
3	18 666 (19.2)	7 060 718 (19)	0.006	18 666 (19.2)	18 790 (19.4)	−0.003
4	17 949 (18.5)	6 748 996 (18.2)	0.009	17 948 (18.5)	17 937 (18.5)	0
5 (least deprived)	18 257 (18.8)	6 651 835 (17.9)	0.024	18 257 (18.8)	18 179 (18.7)	0.002
Smoking status, *n* (%)						
Current smoker	11 090 (11.4)	6 184 540 (16.6)	−0.15	11 090 (11.4)	11 279 (11.6)	−0.006
Ex-smoker	33 971 (35)	12 689 159 (34.1)	0.018	33 971 (35)	34 148 (35.2)	−0.004
Nonsmoker	49 672 (51.2)	16 888 465 (45.4)	0.115	49 668 (51.2)	49 384 (50.9)	0.006
Unknown	2321 (2.4)	1 409 626 (3.8)	−0.081	2320 (2.4)	2238 (2.3)	0.006
BMI class, *n* (%)						
Underweight (<18.5 kg/m^2^)	1200 (1.2)	667 765 (1.8)	−0.046	1200 (1.2)	1222 (1.3)	−0.002
Normal weight (18.5–24.9 kg/m^2^)	22 857 (23.6)	9 975 694 (26.8)	−0.076	22 857 (23.6)	22 940 (23.6)	−0.002
Overweight (25.0–29.9 kg/m^2^)	33 290 (34.3)	12 946 335 (34.8)	−0.011	33 287 (34.3)	33 118 (34.1)	0.004
Obese (≥30.0 kg/m^2^)	35 993 (37.1)	11 376 463 (30.6)	0.137	35 991 (37.1)	36 114 (37.2)	−0.003
Unknown	3714 (3.8)	2 205 533 (5.9)	−0.098	3714 (3.8)	3655 (3.8)	0.003
Comorbidity history, *n* (%)						
Allergic rhinitis	36 697 (37.8)	9 795 616 (26.4)	0.247	36 695 (37.8)	36 505 (37.6)	0.004
Atopic dermatitis	25 065 (25.8)	8 306 651 (22.3)	0.081	25 064 (25.8)	25 053 (25.8)	0
Cancer	7846 (8.1)	3 421 846 (9.2)	−0.04	7846 (8.1)	7929 (8.2)	−0.003
Chronic kidney disease	6609 (6.8)	2 692 281 (7.2)	−0.017	6607 (6.8)	6710 (6.9)	−0.004
Chronic obstructive pulmonary disease	16 447 (16.9)	6 514 676 (17.5)	−0.015	16 447 (16.9)	16 792 (17.3)	−0.009
Dementia	403 (0.4)	352 404 (0.9)	−0.065	403 (0.4)	407 (0.4)	−0.001
Diabetes	11 409 (11.8)	4 771 726 (12.8)	−0.033	11 407 (11.8)	11 510 (11.9)	−0.003
Hypertension	37 370 (38.5)	14 380 000 (38.7)	−0.004	37 368 (38.5)	37 317 (38.5)	0.001
Depression	29 189 (30.1)	9 074 469 (24.4)	0.127	29 187 (30.1)	29 420 (30.3)	−0.005
Sleep disorder	14 097 (14.5)	4 318 473 (11.6)	0.086	14 094 (14.5)	14 054 (14.5)	0.001
Concurrent medications, *n* (%)						
Short-acting beta-agonist	75 090 (77.4)	18 153 217 (48.8)	0.619	75 085 (77.4)	75 494 (77.8)	−0.001
Long-acting beta-agonist	69 609 (71.7)	10 828 920 (29.1)	0.941	69 604 (71.7)	70 134 (72.3)	−0.012
Muscarinic antagonist	18 780 (19.4)	4 824 024 (13)	0.174	18 780 (19.4)	18 808 (19.4)	−0.001
Inhaled corticosteroid	84 287 (86.8)	19 156 597 (51.5)	0.828	84 282 (86.8)	84 977 (87.6)	−0.021
Oral corticosteroid	36 772 (37.9)	4 615 102 (12.4)	0.614	36 767 (37.9)	36 459 (37.6)	0.007
Xanthine-derived bronchodilator	4549 (4.7)	800 864 (2.2)	0.014	4548 (4.7)	4485 (4.6)	0.003
Antihistamine	22 180 (22.9)	4 012 828 (10.8)	0.327	22 178 (22.9)	21 918 (22.6)	0.006

LTRA, leukotriene receptor antagonist; SMD, standardized mean difference; IQR, interquartile range; IMD, Index of Multiple Deprivation; BMI, body mass index.

### Risk of Parkinson’s disease

**Figure 2:**
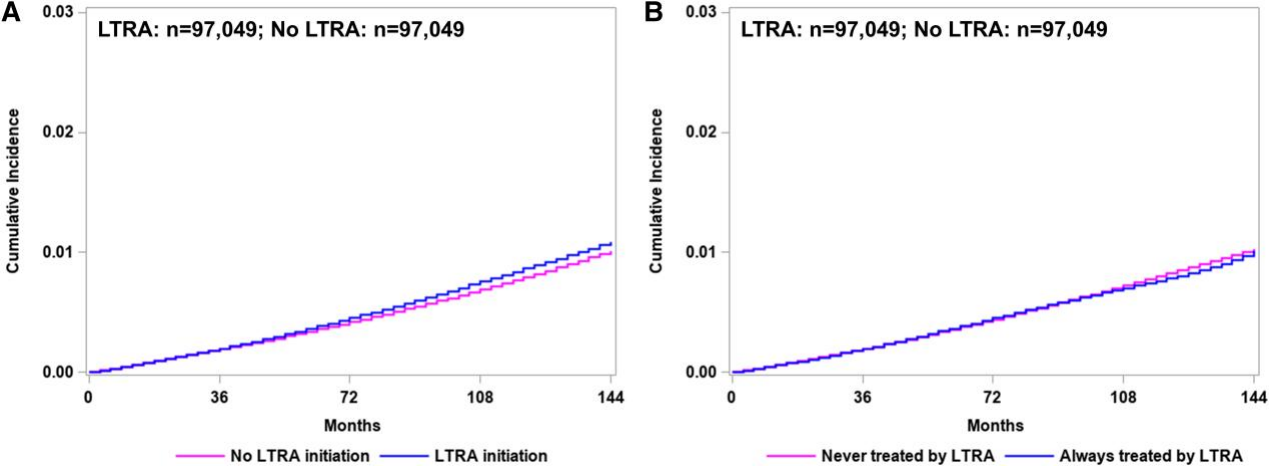
**Cumulative incidence for Parkinson’s disease with leukotriene receptor antagonist (LTRA) treatment and no LTRA treatment among patients with asthma.** (**A**) intention-to-treat effect, 10-year risk ratio: 1.09 (95% CI, 0.94–1.26); (**B**) per protocol effect, 10-year risk ratio: 0.95 (95% CI, 0.75–1.16). The number of patients (*n*) included in each analysis under each treatment group was labelled in each panel. Cumulative incidence curves were constructed using pooled logistic regression models to estimate discrete-time hazards at 3-month intervals. Each data point represents the estimated cumulative risk at the corresponding time point. 95% CIs were calculated using nonparametric bootstrapping with 300 resamplings. No formal test statistic or *P*-value is reported due to the use of nonparametric bootstrap-based inference.

Using the primary definition for Parkinson’s disease (a diagnosis of Parkinson’s disease AND at least two prescriptions of anti-parkinsonian drugs), there were 573 participants on LTRA initiation and 537 propensity score matched participants with no LTRA initiation who developed Parkinson’s disease. The median follow-up for the entire cohort was 5.9 [IQR 3.2–10.2] years in the LTRA group and 5.7 [IQR 3.2–10.0] years in the no LTRA group. Supplementary Fig. 1 shows the distribution of follow-up time by treatment groups. In the intention-to-treat analysis, no difference in the risk of Parkinson’s disease between two treatment arms was observed (5-year RR, 1.06 (95% CI, 0.89–1.25); 10-year RR, 1.09 (95% CI, 0.94–1.26)) (Table 2, Fig. 2). Similar results were observed in the per protocol analysis with dose–response models (5-year RR, 1.03 (95% CI, 0.85–1.22); 10-year RR, 0.95 (95% CI, 0.75–1.16)). Using alternative definitions for Parkinson’s disease, similar results were obtained (Table 2, Supplementary Fig. 2).

**Table 2:** Estimated absolute risks and risk ratios for Parkinson’s disease comparing LTRA treatment with no LTRA treatment

		Intention-to-treat				Per protocol with cumulative dose model			
	No. of events/No. patients	5-year absolute risk (%) (95% CI)	5-year risk ratio (95% CI)	10-year absolute risk (%) (95% CI)	10-year risk ratio (95% CI)	5-year absolute risk (%) (95% CI)	5-year risk ratio (95% CI)	10-year absolute risk (%) (95% CI)	10-year risk ratio (95% CI)
Primary definition									
LTRA	573/ 97 049	0.36 (0.32 to 0.41)	1.06 (0.89 to 1.25)	0.87 (0.79 to 0.96)	1.09 (0.94 to 1.26)	0.37 (0.30 to 0.43)	1.03 (0.85 to 1.22)	0.78 (0.64 to 0.94)	0.95 (0.75 to 1.16)
No LTRA	537/ 97 049	0.34 (0.31 to 0.38)	Reference	0.79 (0.72 to 0.88)	Reference	0.36 (0.32 to 0.39)	Reference	0.83 (0.76 to 0.90)	Reference
Parkinson’s disease by GP record									
LTRA	615/ 97 049	0.38 (0.34 to 0.42)	1.13 (0.96 to 1.33)	0.92 (0.83 to 1.02)	1.12 (0.99 to 1.27)	0.40 (0.34 to 0.47)	1.12 (0.93 to 1.33)	0.84 (0.68 to 1.00)	0.98 (0.79 to 1.19)
No LTRA	567/ 97 049	0.34 (0.30 to 0.38)	Reference	0.82 (0.75 to 0.90)	Reference	0.36 (0.32 to 0.39)	Reference	0.86 (0.78 to 0.93)	Reference
Parkinson’s disease by hospital admission									
LTRA	530/ 97 049	0.26 (0.23 to 0.30)	1.04 (0.83 to 1.23)	0.82 (0.74 to 0.91)	1.11 (0.96 to 1.29)	0.29 (0.23 to 0.35)	1.16 (0.90 to 1.48)	0.80 (0.63 to 0.96)	1.05 (0.82 to 1.30)
No LTRA	489/ 97 049	0.25 (0.23 to 0.28)	Reference	0.73 (0.66 to 0.82)	Reference	0.25 (0.22 to 0.28)	Reference	0.77 (0.70 to 0.84)	Reference
Parkinson’s disease by GP record or a hospital admission									
LTRA	793/ 97 049	0.47 (0.42 to 0.52)	1.08 (0.92 to 1.23)	1.19 (1.10 to 1.30)	1.12 (0.99 to 1.25)	0.50 (0.43 to 0.58)	1.14 (0.95 to 1.33)	1.16 (0.98 to 1.33)	1.06 (0.89 to 1.24)
No LTRA	731/ 97 049	0.43 (0.39 to 0.48)	Reference	1.07 (0.98 to 1.16)	Reference	0.44 (0.41 to 0.48)	Reference	1.10 (1.03 to 11.8)	Reference

LTRA, leukotriene receptor antagonist; CI, confidence interval; GP, general practice.

### Risk of neuropsychiatric events, sleep disorders, and all-cause mortality

In the analysis of neuropsychiatric events, we observed an increased risk of new-onset depression and new-onset sleep disorder, which is consistent in both the intention-to-treat and per protocol analysis. For depression, in the intention-to-treat analysis, the 5-year RR is 1.13 (95% CI, 1.07–1.18), NNH = 154 (95% CI, 112–270); and the 10-year RR is 1.12 (95% CI, 1.07–1.16), NNH = 93 (95% CI, 70–156); in the per protocol analysis, the 5-year RR is 1.19 (95% CI, 1.12–1.26), NNH = 105 (95% CI, 77–159); and the 10-year RR is 1.15 (95% CI, 1.08–1.22), NNH = 75 (95% CI, 52–133). For sleep disorder, in the intention-to-treat analysis, the 5-year RR is 1.14 (95% CI, 1.10–1.19), NNH = 137 (95% CI, 106–204); and the 10-year RR is 1.14 (95% CI, 1.11–1.19), NNH = 77 (95% CI, 58–104); in the per protocol analysis, the 5-year RR is 1.18 (95% CI, 1.11–1.24), NNH = 112 (95% CI, 85–179); and the 10-year RR is 1.12 (95% CI, 1.06–1.19), NNH = 88 (95% CI, 57–189). An increased risk for anxiety was observed in the intention-to-treat analysis (5-year RR, 1.06 (95% CI, 1.02–1.11); 10-year RR, 1.08 (95% CI, 1.03–1.12)) but there was no difference in the per protocol analysis. No increased risk for psychosis was observed (Table 3, Supplementary Fig. 3).

**Table 3:** Estimated absolute risks and risk ratios for neuropsychiatric events and all-cause mortality comparing LTRA treatment with no LTRA treatment

		Intention-to-treat				Per protocol with cumulative dose model			
	No. of events/ No. patients	5-year absolute risk (%) (95% CI)	5-year risk ratio (95% CI)	10-year absolute risk (%) (95% CI)	10-year risk ratio (95% CI)	5-year absolute risk (%) (95% CI)	5-year risk ratio (95% CI)	10-year absolute risk (%) (95% CI)	10-year risk ratio (95% CI)
Anxiety									
LTRA	6052/ 72 356	6.26 (6.07 to 6.46)	1.06 (1.02 to 1.11)	11.44 (11.11 to 11.81)	1.08 (1.03 to 1.12)	5.83 (5.56 to 6.15)	0.97 (0.92 to 1.03)	10.45 (9.91 to 11.00)	0.97 (0.92 to 1.03)
No LTRA	5595/ 71 806	5.89 (5.70 to 6.06)	Reference	10.62 (10.29 to 10.89)	Reference	5.99 (5.82 to 6.13)	Reference	10.73 (10.45 to 10.99)	Reference
Depression									
LTRA	4890/ 67 546	5.63 (5.43 to 5.82)	1.13 (1.07 to 1.18)	9.84 (9.54 to 10.19)	1.12 (1.07 to 1.16)	5.99 (5.67 to 6.29)	1.19 (1.12 to 1.26)	10.17 (9.54 to 10.69)	1.15 (1.08 to 1.22)
No LTRA	4309/ 66 663	5.00 (4.84 to 5.14)	Reference	8.80 (9.53 to 9.03)	Reference	5.02 (4.87 to 5.16)	Reference	8.81 (8.56 to 9.05)	Reference
Psychosis									
LTRA	1572/ 94 774	0.86 (0.81 to 0.93)	0.88 (0.81 to 0.97)	2.20 (2.06 to 2.36)	0.90 (0.83 to 0.98)	0.95 (0.85 to 1.06)	1.00 (0.87 to 1.14)	2.74 (2.42 to 3.04)	1.13 (0.99 to 1.27)
No LTRA	1706/ 93 826	0.98 (0.91 to 1.04)	Reference	2.45 (2.32 to 2.56)	Reference	0.95 (0.89 to 1.01)	Reference	2.42 (2.29 to 2.55)	Reference
Sleep disorders									
LTRA	6161/ 82 555	5.70 (5.52 to 5.88)	1.14 (1.10 to 1.19)	10.40 (10.13 to 10.74)	1.14 (1.11 to 1.19)	6.00 (5.69 to 6.22)	1.18 (1.11 to 1.24)	10.35 (9.76 to 11.00)	1.12 (1.06 to 1.19)
No LTRA	5306/ 81 822	4.98 (4.83 to 5.14)	Reference	9.10 (8.83 to 9.37)	Reference	5.07 (4.83 to 5.21)	Reference	9.22 (8.99 to 9.43)	Reference
All-cause mortality									
LTRA	13 583/ 97 049	7.47 (7.31 to 7.66)	0.84 (0.81 to 0.87)	17.76 (17.44 to 18.10)	0.94 (0.92 to 0.96)	7.41 (7.14 to 7.70)	0.86 (0.82 to 0.89)	18.01 (17.44 to 18.59)	0.94 (0.91 to 0.98)
No LTRA	14 551/ 97 049	8.91 (8.72 to 9.10)	Reference	18.96 (18.63 to 19.26)	Reference	8.67 (8.50 to 8.83)	Reference	19.08 (18.77 to 19.36)	Reference
Composite of Parkinson’s disease and all-cause mortality									
LTRA	13 995/ 97 049	8.16 (7.99 to 8.35)	0.89 (0.86 to 0.91)	18.69 (18.39 to 19.04)	0.95 (0.93 to 0.97)	8.50 (8.21 to 8.81)	0.93 (0.90 to 0.98)	20.00 (19.41 to 20.64)	1.00 (0.97 to 1.04)
No LTRA	14 928/ 97 049	9.22 (9.03 to 9.41)	Reference	19.66 (19.31 to 19.99)	Reference	9.09 (8.92 to 9.26)	Reference	19.91 (19.58 to 20.22)	Reference

LTRA, leukotriene receptor antagonist; CI, confidence interval.

We found a lower risk of all-cause mortality with LTRA treatment. The risk reduction was observed early in the follow-up. The 5-year RR was 0.84 (95% CI, 0.81–0.97) in the intention-to-treat analysis, NNT = 69 (95% CI, 60–88); and 0.86 (95% CI, 0.82–0.89) in the per protocol analysis NNT = 81 (95% CI, 63–111). While the 10-year estimate was 0.94 (95% CI, 0.92–0.96) in the intention-to-treat analysis, NNT = 84 (95% CI, 63–132), and 0.94 (95% CI, 0.91–0.98) in the per protocol analysis, NNT = 96 (95% CI, 59–250). When analyzing Parkinson’s disease and mortality as a composite outcome, there was a small risk difference in the intention-to-treat analysis (10-year RR, 0.95 (95% CI, 0.93–0.97)), but not in the per protocol analysis (10-year RR, 1.00 (95% CI, 0.97–1.04)) (Table 3, Supplementary Fig. 4).

### Sensitivity analysis

The results are consistent when we excluded LTRA re-initiators from the analysis, repeated the sequential trial emulation on the scale of calendar months, or require a minimum of 5-year record history in the database (Supplementary Tables 5–7). When we applied a 3-year lead time in ascertaining Parkinson’s diagnosis, the RRs ranged from 0.88 to 1.13 for varied definitions of Parkinson’s disease in the intention-to-treat and per protocol analyses and the results remained statistical nonsignificant (Supplementary Table 8). For the exploratory outcomes on anosmia, constipation, dizziness, and urinary incontinence (the autonomic and sensory presentations associated with Parkinson’s disease), the estimates were largely consistent with those for Parkinson’s disease in the primary analysis. The exceptions were anosmia with a 10-year RR of 1.32 in the intention-to-treat analysis and 1.19 in the per protocol analysis, and urinary incontinence in the per protocol analysis where the risk with LTRA treatment was higher at 10-years (RR, 1.23) (Supplementary Table 9). In the age- and sex-stratified analysis, the relative risk of Parkinson’s disease associated with LTRA treatment compared with no LTRA appeared higher in women than men (*P*-value for interaction = 0.02 in intention-to-treat analysis, and 0.03 in per protocol analysis). LTRA treatment is associated with a higher risk of Parkinson’s disease among women. No interaction was observed for age (Supplementary Table 10).

## Discussion

We explicitly emulated a hypothetical target trial of LTRA treatment and the risk of being diagnosed with Parkinson’s disease among asthma patients aged 50–84 years. Overall, we did not find evidence for a lower risk of Parkinson’s disease with LTRA treatment compared with no LTRA treatment, with up to 12 years of treatment, with consideration of mortality as a competing risk event for being diagnosed with Parkinson’s disease. Our findings also suggest potential higher risks of neuropsychiatric events with LTRA treatment in this population, especially for sleep disorders and depression, which are consistent with the recent MHRA warnings.^12^

Following the target trial emulation framework, our study defined LTRA treatment as a clearly defined and clinically realistic intervention. Specifically, we estimated the average treatment effect of the ‘initiation and continued use of LTRA medications’ on Parkinson’s disease, rather than examining the association between the ‘previous or current LTRA treatment status’ and Parkinson’s disease, as evaluated in the previous study.^9^ This is because a clinician can only realistically manage a patient by initiating a new treatment, but not by altering their treatment history. The sequential trial emulation approach used in this study, though initially appearing complex, is crucial for accurately emulating the target trial and thus match for the baseline risks and avoid bias from inappropriate study designs, and obtain clinically relevant effect estimates. This method allowed us to start patient follow-up at the point of eligibility assessment and treatment assignment, effectively mitigating common biases in observational studies, such as selection bias and immortal time bias.^26^ The difference in study design and analytical approach may account for the discrepancy between our findings and those in the previous report.^9^ In particular, the study by Liu *et al*. found an association between a lower risk of Parkinson’s disease diagnosis and prior high exposure to montelukast. To further investigate, we conducted a per protocol analysis using a dose–response model to assess the effect of sustained LTRA treatment on Parkinson’s disease. Utilizing high-quality electronic health records from the UK, we addressed several acknowledged limitations of the previous study, including controlling asthma severity, smoking status, and extending the follow-up period. Our results do not support a protective effect of LTRA treatment on the risk of incident Parkinson’s disease diagnosis, and no dose–response relationship with the risk of Parkinson’s disease was observed. However, we observed a higher relative risk of Parkinson’s disease with LTRA treatment in female patients. Further studies may confirm this finding.

The concerns about potential neuropsychiatric effects associated with LTRA are also highly relevant in the assessment of their potential for managing Parkinson’s disease. Neuropsychiatric symptoms such as depression and anxiety, alongside sleep disturbances, are well-established as prodromal symptoms, risk factors, and clinical presentations of Parkinson’s disease.^13,27^ Therefore, understanding the neuropsychiatric profile of LTRA is crucial in considering their suitability for repurposing in Parkinson’s disease management. The risks of neuropsychiatric events associated with LTRA treatment have been investigated in previous observational studies, but only two studies (both using Korean cohorts) included older adults, and their findings are conflicting.^28^ One case–control study reported an odds ratio of 1.67^29^ for the neuropsychiatric events overall, while the cohort study reported a hazard ratio of 1.01.^30^ However, since the time zero (index date) in these two studies is not clearly or appropriately defined as in a target trial emulation study, their estimates may be affected by potential prevalent user bias or immortal time bias. Our study, using a rigorous framework for observational analysis, is therefore valuable in confirming the neuropsychiatric risks associated with LTRA, particularly in middle-age and older patients, where evidence is lacking. Any therapeutic intervention involving LTRA should take these effects into account, as worsening these symptoms could potentially reduce the overall benefit in managing allergic conditions or neurodegenerative disorders like Parkinson’s disease.

In 2024, over 100 active clinical trials are evaluating potential therapies for Parkinson's disease, with nearly 40% focusing on repurposed treatments.^4,31^ This strategy is both efficient and attractive for drug development. The International Linked Clinical Trials (iLCT) programme, running for over a decade, has been leveraging the repurposing approach to accelerate the development of new therapies for Parkinson’s disease.^32^ Epidemiology data plays a crucial role in the iLCT process, serving as contributory evidence in the selection of drug candidates for formal clinical evaluation. These data can provide valuable real-world insights on drug safety, usage patterns, and off-label benefits, helping to de-risk clinical trials. However, an epidemiological study can be misleading or difficult to interpret if its design or analysis is inappropriate or biased. Explicitly emulating a target trial in epidemiological analysis, which helps prevent common biases in such studies, is needed to enhance the drug repurposing process. Overall, our current study does not support the use of LTRA for Parkinson’s disease prevention. However, we have not explored all potential avenues for repurposing LTRA in Parkinson’s. For instance, due to limitations in routinely collected data, we were unable to examine the effects of higher daily doses or alternative formulations not yet used clinically, or LTRA initiation after Parkinson’s disease diagnosis. These aspects could be addressed in future prospective studies, although the neuropsychiatric risks may discourage such efforts.

Our study is the first study applying the robust target trial emulation framework to investigate the effect of LTRA on the risk of Parkinson’s disease and neuropsychiatric events. Using a sequential trial emulation approach, we minimised the risk of common bias in observational analyses.^26,33^ Leveraging high-quality electronic health records, we were able to adjust for several key confounders that have impacted other studies, such as smoking and asthma severity.^34,35^ We provided various estimates on the treatment effect, including the effect of treatment initiation and sustained treatment, using absolute risks and cumulative incidence curves, allowing a comprehensive interpretation of our findings.

There are limitations to our study. Firstly, our study aimed to answer a causal question by explicitly emulating a target trial, but the causal interpretation relies on an assumption of no residual confounding. This is unverifiable, and the residual confounding is unlikely to be completely ruled out given the observational nature of our study. Although the measured baseline characteristics are similar between two treatment groups, some unmeasured variables such as physical exercise or caffeine intake may still be imbalanced. Secondly, the complex aetiology of Parkinson’s disease may affect the validity of the clinical diagnosis of Parkinson’s disease.^36^ However, we employed several case definitions with varying stringency to capture Parkinson’s disease diagnosis, as well as explored the risk of prodromal symptoms of Parkinson’s disease. The results are highly consistent.^37^ Of note, the primary definition has been validated in the database with 90% specificity.^38^ Thirdly, we relied on the prescription records to assign the LTRA treatment status to patients, but we did not know if the patients redeemed or consumed the prescribed medications as directed. Therefore, misclassification of treatment is possible. Lastly, the median follow-up of approximately 6 years may be insufficient to fully capture Parkinson’s disease onset, which may have a prodromal period over 10 years. Rather than reflecting true prevention, our findings may better represent phenoconversion of prodromal Parkinson’s disease among older patients with relative high incidence of Parkinson’s disease.^1,39^ In the sensitivity analysis, we applied a 3-year lead period to that should reflect most diagnostic delays. Furthermore, our analysis of prodromal symptoms yielded consistent results, reinforcing our conclusions.

In summary, our study found no meaningful difference in the risk of Parkinson’s disease diagnosis with LTRA treatment, but did identify a higher risk of depression and sleep disorders in older patients with asthma. These findings do not support repurposing the current LTRA treatment strategy for asthma to manage Parkinson’s disease. Reduction in Parkinson’s disease risk is not necessarily equivalent to slowing disease progression after onset hence future studies might still explore LTRA at varying doses, formulations, and in patients at different stages or risk levels of Parkinson’s disease to more fully assess their potential. Neuropsychiatric risks must be carefully considered when evaluating any therapeutic role for LTRA in Parkinson’s disease management.

## Supplementary Material

fcaf340_Supplementary_Data

## Data Availability

This study is based on data from the CPRD, obtained under license from the UK MHRA. The data are not publicly available and can only be accessed by approved researchers who have obtained the necessary permissions from CPRD. Access to CPRD data requires protocol submission and approval by the Independent Scientific Advisory Committee (ISAC). The authors do not have permission to share the data. The statistical codes are available at https://github.com/ChengshengJu/LTRA-and-PD.
