# Supplementary material for: Effect of leukotriene receptor antagonist use on the future risk of Parkinson’s disease in older patients with asthma
Source: Brain Commun. 2025 Sep 10;7(5):fcaf340. doi: 10.1093/braincomms/fcaf340 (PMC12455039; doi:10.1093/braincomms/fcaf340)
Supplement: fcaf340_Supplementary_Data [file fcaf340_supplementary_data.pdf]

Effect of leukotriene receptor antagonist use on the future risk of Parkinson's disease in older patients with asthma: a longitudinal cohort study

Supplementary materials

Contents

Supplementary Table 1. Specifications of the target pragmatic trial and observational trial emulation....3

Supplementary Table 2. List of diagnostic codes for study outcomes. 6

Supplementary Table 3. Description of the number of eligible patients, number of Parkinson's disease cases, and mean follow-up in each emulated trial, before and after matching....49

Supplementary Table 4. Full baseline characteristics of patients who initiated LTRA treatment and did not initiate LTRA treatment pooling all emulated trials, before and after matching (pooled emulated trials). 56

Supplementary Figure 1. Histograms showing the distribution of follow-up time by treatment groups....60

Supplementary Figure 2. Cumulative incidence of Parkinson's disease with alternative definitions with leukotriene receptor antagonist (LTRA) treatment and no LTRA treatment among patients with asthma....61

Supplementary Figure 3. Cumulative incidence of neuropsychiatric events and sleep disorders with leukotriene receptor antagonist (LTRA) treatment and no LTRA treatment among patients with asthma....64

Supplementary Figure 4. Cumulative incidence of mortality with leukotriene receptor antagonist (LTRA) treatment and no LTRA treatment among patients with asthma....65

Supplementary Table 5. Sensitivity analysis: point estimates of the absolute risks and risk ratios for Parkinson's disease comparing LTRA treatment versus no LTRA treatment, excluding reinitiators of LTRA and their matched pairs. 66

Supplementary Table 6. Sensitivity analysis: point estimates of the absolute risks and risk ratios for Parkinson's disease comparing LTRA treatment versus no LTRA treatment, sequential trials by calendar time....67

Supplementary Table 7. Sensitivity analysis: point estimates of the absolute risks and risk ratios absolute risks and risk ratios for Parkinson's disease comparing LTRA treatment versus no LTRA treatment, with at least five year record history. 68

Supplementary Table 8. Sensitivity analysis: point estimates of the absolute risks and risk ratios for Parkinson's disease comparing LTRA treatment versus no LTRA treatment, with a 3-year lead time. 69

Supplementary Table 9. Sensitivity analysis: point estimates of the absolute risks and risk ratios for anosmia, constipation, dizziness, and urinary incontinence (autonomic and sensory presentations associated with Parkinson's) comparing LTRA treatment versus no LTRA treatment. 70

Supplementary Table 10. Sensitivity analysis: point estimates of the absolute risks and risk ratios for Parkinson's disease comparing LTRA treatment versus no LTRA treatment, stratified by age groups and sex. 71

Supplementary Table 1. Specifications of the target pragmatic trial and observational trial emulation.

Sections	Component	Target trial specification	Target trial emulation
Causal Estimand	Eligibility criteria	Between January 2000 to December 2020, eligible patients are required to be 50 to 84 years old and have a history of asthma diagnosis; other criteria include no history of Parkinson's disease or secondary Parkinsonism, no history of dopaminergic anti-parkinsonian treatment, no LTRA treatment within the past 365 days.	Same as the target trial.<br>Patients are additionally required to have at least one year of up-to-standard record history with the current GP practice in the database.
	Treatment strategies	(1) Initiation and sustained treatment of LTRA since baseline.<br>(2) No initiation of LTRA treatment over follow-up.	Same as the target trial.
	Treatment assignment	Patients are randomly assigned to a strategy at baseline and will be aware of the strategy they have been assigned.	Patients are assigned to the treatment strategy that their data are compatible with at the baseline, e.g., patients are assigned to the LTRA treatment arm if they actually initiated LTRA treatment at baseline. Randomisation is emulated via propensity score (PS) matching technique ensure balanced baseline characteristics between patient cohorts.
	Outcomes	Primary outcome is Parkinson's disease.<br>Secondary outcomes include anxiety, sleep disorders, depression, and psychosis; and all-cause mortality	Same as the target trial.<br>Various definitions of Parkinson's disease are used:<br>• Having a GP record or a hospital admission due to Parkinson's disease AND at least two prescriptions of anti-parkinsonian drugs (levodopa, dopamine receptor agonists, or monoamine-oxidase-B inhibitors).<br>• Having a GP record of Parkinson's disease.<br>• Having a hospital admission due to Parkinson's disease.<br><br>Having a GP record or a hospital admission due to Parkinson's disease.

	Follow-up	For each patient, the follow-up starts at baseline (i.e., the entry point of the trial) and ends at the Parkinson's disease diagnosis or other censoring events, including secondary Parkinsonism, death, drop out from the study, whichever occurs first.	For each patient, the follow-up starts at baseline (i.e., the entry point of the trial) and ends at the Parkinson's disease diagnosis or other censoring events, including death, transfer out of practice, end of data collection for CPRD, whichever occurs first.<br>We will emulate multiple sequential nested trials recruiting patients aged from 50 to 84 at 3-month age gap. That is, the first trial emulation includes patient who are eligible at the age 50 month 1 to month 3; the second trial includes patients aged 50 month 4 to month 6; the third trial includes patients aged 50 month 7 to month 9 … There will be 140 trials in total.<br>The baseline for each trial is defined as the date of treatment assignment.
	Causal contrasts	Intention-to-treat effect – the effect of treatment assignment.<br>Per protocol effect – the effect of adhering to the assigned treatment.	Observational analogue of intention-to-treat effect – the effect of treatment initiation.<br>Observational analogue of per protocol effect – cumulative dose-response model-based effect of always treated versus never treated.
Assumptions	Intention-to-treat effect	Conditional exchangeability can be achieved by randomised treatment assignment.<br>Loss to follow-up is non-informative.<br><br>To handle the competing risk from pre-Parkinson's disease death, we estimate the controlled direct effect, in which we assumed a hypothetical scenario that no death can occur during follow-up, and we assume no confounding between death and Parkinson's disease.<br>In a sensitivity analysis, we estimate the effect on composite outcome of death and Parkinson's disease.	Same as the target trial, except that randomization is emulated via propensity score matching.<br>The following baseline covariates are used for the estimation of propensity score:<br>sex, duration of asthma, number of hospitalisations in the past year, number of asthma-related hospitalisations in the past year, calendar year of cohort entry (, Index of IMD score, smoking status, BMI categories, eosinophilia in the past year, frailty, allergic rhinitis, atopic dermatitis, alcohol-related disorder, cancer, chronic kidney disease, chronic obstructive pulmonary disease, dementia, diabetes, gout, liver disease, hypertension, myocardial infarction, stroke, depression, sleep disorder, epilepsy, psychosis, head injury, fall, fracture, lower

			respiratory tract infection, influenza infection, short-acting beta-agonists, long-acting beta-agonists, muscarinic antagonists, inhaled corticosteroids, oral corticosteroids, xanthine-derived bronchodilators, antihistamines, low-dose aspirin, calcium-channel blockers, statins, anxiolytics and sedatives, antidepressants, antipsychotics, metformin, incretin-based antidiabetic drugs, insulin.
	Per-protocol effect	Conditional exchangeability can be achieved by randomised treatment assignment.<br>Loss to follow-up is non-informative.<br>Treatment deviation is non-informative.<br><br>To handle the competing risk from pre-Parkinson's disease death, we estimate the controlled direct effect, in which we assumed a hypothetical scenario that no death can occur during follow-up, and we assume no confounding between death and Parkinson's disease.<br>In a sensitivity analysis, we estimate the effect on composite outcome of death and Parkinson's disease.	Same as the target trial, except that randomization is emulated via propensity score matching. The same model as the intention-to-treat analysis is used.<br>An observational analogue of the per-protocol effect is estimated using a dose-response that extrapolates the effect of always being treated by LTRA versus never being treated by LTRA.
Estimator	-	Intention-to-treat analysis: no adjustment of confounding is required as confounding does not exist in the intention-to-treat analysis after randomisation.<br>Per protocol analysis: patients are censored when they deviate from the assigned treatment strategy over follow-up.	Both the intention-to-treat analysis and per protocol analysis are conducted with sequential emulation of target trials to improve statistical efficiency. Pooled data from all trial emulations are used to estimate the overall treatment effect.

Supplementary Table 2. List of diagnostic codes for study outcomes.

Coding system	Code	Description	Outcome
Read	297A.00	O/E - Parkinsonian tremor	Parkinson’s disease
Read	F12z.00	Parkinson's disease NOS	Parkinson’s disease
Read	2994.11	O/E - Parkinson gait	Parkinson’s disease
Read	F120.00	Paralysis agitans	Parkinson’s disease
Read	2987.11	O/E - Parkinson posture	Parkinson’s disease
Read	F12..00	Parkinson's disease	Parkinson’s disease
Read	2987.00	O/E -Parkinson flexion posture	Parkinson’s disease
Read	2994.00	O/E-festination-Parkinson gait	Parkinson’s disease
Read	F130300	Parkinsonism with orthostatic hypotension	Parkinson’s disease
Read	Eu02300	[X]Dementia in Parkinson's disease	Parkinson’s disease
Read	F11x900	Cerebral degeneration in Parkinson's disease	Parkinson’s disease
ICD-10	G20	Parkinson's disease	Parkinson’s disease
SNOMED-CT	163672003	O/E - Parkinsonian tremor	Parkinson’s disease
SNOMED-CT	163681009	O/E -Parkinson flexion posture	Parkinson’s disease
SNOMED-CT	425390006	Dementia in Parkinsons disease	Parkinson’s disease
SNOMED-CT	49049000	Parkinson's disease NOS	Parkinson’s disease
SNOMED-CT	718685006	Orthostatic hypotension co-occurrent and due to Parkinson's disease	Parkinson’s disease
SNOMED-CT	49049000	Idiopathic Parkinson's disease	Parkinson’s disease
SNOMED-CT	268964003	O/E-festination-Parkinson gait	Parkinson’s disease
SNOMED-CT	341551000000108	Cerebral degeneration in Parkinson's disease	Parkinson’s disease
SNOMED-CT	425390006	Dementia in Parkinsons disease	Parkinson’s disease
SNOMED-CT	49049000	Idiopathic Parkinson's disease	Parkinson’s disease
Read	Eu41100	[X]Generalized anxiety disorder	Anxiety
Read	E202D00	Fear of death	Anxiety
Read	Eu40213	[X]Claustrophobia	Anxiety
Read	Eu40100	[X]Social phobias	Anxiety
Read	146G.00	H/O: agoraphobia	Anxiety
Read	1B1V.00	C/O - panic attack	Anxiety
Read	Eu41200	[X]Mixed anxiety and depressive disorder	Anxiety
Read	Eu40300	[X]Needle phobia	Anxiety
Read	Eu40214	[X]Simple phobia	Anxiety
Read	E202200	Agoraphobia without mention of panic attacks	Anxiety
Read	E202z00	Phobic disorder NOS	Anxiety
Read	E20z.00	Neurotic disorder NOS	Anxiety
Read	Eu40012	[X]Panic disorder with agoraphobia	Anxiety
Read	E202B00	Cancer phobia	Anxiety
Read	Eu34114	[X]Persistant anxiety depression	Anxiety
Read	285..00	Neurotic condition, insight present	Anxiety
Read	E202300	Social phobia, fear of eating in public	Anxiety
Read	E202.11	Social phobic disorders	Anxiety

Read	Eu40011	[X]Agoraphobia without history of panic disorder	Anxiety
Read	E202800	Claustrophobia	Anxiety
Read	E200400	Chronic anxiety	Anxiety
Read	Eu40212	[X]Animal phobias	Anxiety
Read	E202500	Social phobia, fear of public washing	Anxiety
Read	1Bb..00	Specific fear	Anxiety
Read	225J.00	O/E - panic attack	Anxiety
Read	E202.00	Phobic disorders	Anxiety
Read	E202000	Phobia unspecified	Anxiety
Read	E202C00	Dental phobia	Anxiety
Read	Eu41z00	[X]Anxiety disorder, unspecified	Anxiety
Read	Eu41y00	[X]Other specified anxiety disorders	Anxiety
Read	Eu41z11	[X]Anxiety NOS	Anxiety
Read	Eu40000	[X]Agoraphobia	Anxiety
Read	Z481.00	Phobia counselling	Anxiety
Read	225K.00	O/E - fearful mood	Anxiety
Read	Eu40y00	[X]Other phobic anxiety disorders	Anxiety
Read	E202600	Acrophobia	Anxiety
Read	Eu41y11	[X]Anxiety hysteria	Anxiety
Read	8HHp.00	Referral for guided self-help for anxiety	Anxiety
Read	E202700	Animal phobia	Anxiety
Read	E201B00	Compensation neurosis	Anxiety
Read	E202100	Agoraphobia with panic attacks	Anxiety
Read	E202900	Fear of crowds	Anxiety
Read	E202400	Social phobia, fear of public speaking	Anxiety
Read	Eu40z00	[X]Phobic anxiety disorder, unspecified	Anxiety
Read	1466.00	H/O: anxiety state	Anxiety
Read	Eu41112	[X]Anxiety reaction	Anxiety
Read	E20y100	Writer's cramp neurosis	Anxiety
Read	E20y200	Other occupational neurosis	Anxiety
Read	E200100	Panic disorder	Anxiety
Read	Eu41012	[X]Panic state	Anxiety
Read	E202A00	Fear of flying	Anxiety
Read	E20y.00	Other neurotic disorders	Anxiety
Read	Eu40112	[X]Social neurosis	Anxiety
Read	E20yz00	Other neurotic disorder NOS	Anxiety
Read	Eu41300	[X]Other mixed anxiety disorders	Anxiety
Read	E200z00	Anxiety state NOS	Anxiety
Read	E200111	Panic attack	Anxiety
Read	E200500	Recurrent anxiety	Anxiety
Read	E200200	Generalised anxiety disorder	Anxiety
Read	Eu41113	[X]Anxiety state	Anxiety
Read	ZV11200	[V]Personal history of neurosis	Anxiety

Read	E20..00	Neurotic disorders	Anxiety
Read	286..00	Poor insight into neurotic condition	Anxiety
Read	9N54.00	Encounter for fear	Anxiety
Read	1B1H.11	Fear	Anxiety
Read	Eu41.00	[X]Other anxiety disorders	Anxiety
Read	E202E00	Fear of pregnancy	Anxiety
Read	8G52.00	Antiphobic therapy	Anxiety
Read	E200.00	Anxiety states	Anxiety
Read	Eu41011	[X]Panic attack	Anxiety
Read	E200300	Anxiety with depression	Anxiety
Read	Eu40z12	[X]Phobic state NOS	Anxiety
Read	Eu40211	[X]Acrophobia	Anxiety
Read	E200000	Anxiety state unspecified	Anxiety
Read	E20y300	Psychasthenic neurosis	Anxiety
Read	Eu40z11	[X]Phobia NOS	Anxiety
Read	Eu34113	[X]Neurotic depression	Anxiety
Read	Eu41211	[X]Mild anxiety depression	Anxiety
Read	Z4L1.00	Anxiety counselling	Anxiety
Read	Eu41000	[X]Panic disorder [episodic paroxysmal anxiety]	Anxiety
Read	Eu34111	[X]Depressive neurosis	Anxiety
Read	8G94.00	Anxiety management training	Anxiety
Read	Eu40.00	[X]Phobic anxiety disorders	Anxiety
Read	Eu41111	[X]Anxiety neurosis	Anxiety
Read	Eu40200	[X]Specific (isolated) phobias	Anxiety
Read	E202.12	Phobic anxiety	Anxiety
SNOMED-CT	16265951000119109	Simple phobia	Anxiety
SNOMED-CT	16265951000119109	Combat fatigue	Anxiety
SNOMED-CT	16265951000119109	O/E - anxious	Anxiety
SNOMED-CT	16265951000119109	Anxiety state unspecified	Anxiety
SNOMED-CT	16265951000119109	Chronic anxiety	Anxiety
SNOMED-CT	16265951000119109	Recurrent anxiety	Anxiety
SNOMED-CT	16265951000119109	Anxiety state	Anxiety
SNOMED-CT	16265951000119109	Agoraphobia with panic attacks	Anxiety
SNOMED-CT	16265951000119109	Agoraphobia without history of panic disorder	Anxiety
SNOMED-CT	16265951000119109	Social phobia, fear of eating in public	Anxiety
SNOMED-CT	16265951000119109	Social phobia, fear of public speaking	Anxiety
SNOMED-CT	16265951000119109	Social phobia, fear of public washing	Anxiety
SNOMED-CT	16265951000119109	Anancastic neurosis	Anxiety
SNOMED-CT	16265951000119109	Compulsive neurosis	Anxiety
SNOMED-CT	16265951000119109	Obsessional neurosis	Anxiety
SNOMED-CT	16265951000119109	Obsessive-compulsive disorder NOS	Anxiety
SNOMED-CT	16265951000119109	Psychogenic rumination	Anxiety
SNOMED-CT	16265951000119109	Acute panic state due to acute stress reaction	Anxiety

SNOMED-CT	16265951000119109	Acute fugue state due to acute stress reaction	Anxiety
SNOMED-CT	16265951000119109	Acute stupor state due to acute stress reaction	Anxiety
SNOMED-CT	16265951000119109	Other acute stress reactions	Anxiety
SNOMED-CT	16265951000119109	Acute situational disturbance	Anxiety
SNOMED-CT	16265951000119109	Other acute stress reaction NOS	Anxiety
SNOMED-CT	16265951000119109	Other post-traumatic stress disorder	Anxiety
SNOMED-CT	16265951000119109	Childhood and adolescent fearfulness disturbance	Anxiety
SNOMED-CT	16265951000119109	[X]Phobic anxiety disorders	Anxiety
SNOMED-CT	16265951000119109	[X]Other phobic anxiety disorders	Anxiety
SNOMED-CT	16265951000119109	[X]Phobic anxiety disorder, unspecified	Anxiety
SNOMED-CT	16265951000119109	[X]Other anxiety disorders	Anxiety
SNOMED-CT	16265951000119109	[X]Panic disorder [episodic paroxysmal anxiety]	Anxiety
SNOMED-CT	16265951000119109	[X]Other mixed anxiety disorders	Anxiety
SNOMED-CT	16265951000119109	[X]Anxiety disorder, unspecified	Anxiety
SNOMED-CT	16265951000119109	[X]Mixed obsessional thoughts and acts	Anxiety
SNOMED-CT	16265951000119109	[X]Other obsessive-compulsive disorders	Anxiety
SNOMED-CT	16265951000119109	[X]Obsessive-compulsive disorder, unspecified	Anxiety
SNOMED-CT	16265951000119109	Separation anxiety disorder of childhood	Anxiety
SNOMED-CT	16265951000119109	Anxiety state	Anxiety
SNOMED-CT	16265951000119109	Panic attack	Anxiety
SNOMED-CT	16265951000119109	Stage fright	Anxiety
SNOMED-CT	16265951000119109	Examination fear	Anxiety
SNOMED-CT	16265951000119109	Phobic disorder	Anxiety
SNOMED-CT	16265951000119109	Acute stress reaction NOS	Anxiety
SNOMED-CT	16265951000119109	[X]Other specified anxiety disorders	Anxiety
SNOMED-CT	16265951000119109	Anxious mother	Anxiety
SNOMED-CT	16265951000119109	Maternal concern	Anxiety
SNOMED-CT	16265951000119109	Separation anxiety disorder	Anxiety
SNOMED-CT	16265951000119109	Occupation-related stress disorder	Anxiety
SNOMED-CT	16265951000119109	[X]Other physical and mental strain related to work	Anxiety
SNOMED-CT	16265951000119109	Generalised anxiety disorder	Anxiety
SNOMED-CT	16265951000119109	Acute stress reaction	Anxiety
SNOMED-CT	16265951000119109	Panic disorder	Anxiety
SNOMED-CT	16265951000119109	Adjustment reaction with anxious mood	Anxiety
SNOMED-CT	16265951000119109	C/O - panic attack	Anxiety
SNOMED-CT	16265951000119109	Anxiousness	Anxiety
SNOMED-CT	16265951000119109	O/E - panic attack	Anxiety
SNOMED-CT	16265951000119109	Stress reaction causing mixed disturbance of emotion and conduct	Anxiety
SNOMED-CT	16265951000119109	Social phobic disorders	Anxiety

SNOMED-CT	16265951000119109	Phobic disorders	Anxiety
SNOMED-CT	16265951000119109	Phobic anxiety disorder	Anxiety
SNOMED-CT	16265951000119109	Obsessive-compulsive disorder	Anxiety
SNOMED-CT	16265951000119109	[X]Acrophobia	Anxiety
SNOMED-CT	16265951000119109	[X]Acute crisis reaction	Anxiety
SNOMED-CT	16265951000119109	[X]Acute reaction to stress	Anxiety
SNOMED-CT	16265951000119109	[X]Acute stress reaction	Anxiety
SNOMED-CT	16265951000119109	Agoraphobia	Anxiety
SNOMED-CT	16265951000119109	[X]Agoraphobia without history of panic disorder	Anxiety
SNOMED-CT	16265951000119109	[X]Anankastic neurosis	Anxiety
SNOMED-CT	16265951000119109	[X]Anthropophobia	Anxiety
SNOMED-CT	16265951000119109	[X]Anxiety hysteria	Anxiety
SNOMED-CT	16265951000119109	Anxiety neurosis	Anxiety
SNOMED-CT	16265951000119109	[X]Anxiety NOS	Anxiety
SNOMED-CT	16265951000119109	[X]Anxiety reaction	Anxiety
SNOMED-CT	16265951000119109	[X]Anxiety state	Anxiety
SNOMED-CT	16265951000119109	Anxious personality disorder	Anxiety
SNOMED-CT	16265951000119109	[X]Avoidant disorder childhood	Anxiety
SNOMED-CT	16265951000119109	[X]Childhood overanxious disorder	Anxiety
SNOMED-CT	16265951000119109	[X]Claustrophobia	Anxiety
SNOMED-CT	16265951000119109	[X]Combat fatigue	Anxiety
SNOMED-CT	16265951000119109	[X]Crisis state	Anxiety
SNOMED-CT	16265951000119109	[X]Generalized anxiety disorder	Anxiety
SNOMED-CT	16265951000119109	[X]Mild anxiety depression	Anxiety
SNOMED-CT	16265951000119109	[X]Mixed anxiety and depressive disorder	Anxiety
SNOMED-CT	16265951000119109	[X]Obsessive - compulsive disorder	Anxiety
SNOMED-CT	16265951000119109	[X]Obsessive-compulsive neurosis	Anxiety
SNOMED-CT	16265951000119109	Organic anxiety disorder	Anxiety
SNOMED-CT	16265951000119109	[X]Panic attack	Anxiety
SNOMED-CT	16265951000119109	[X]Panic disorder with agoraphobia	Anxiety
SNOMED-CT	16265951000119109	[X]Panic state	Anxiety
SNOMED-CT	16265951000119109	[X]Persistant anxiety depression	Anxiety
SNOMED-CT	16265951000119109	Phobic disorder	Anxiety
SNOMED-CT	16265951000119109	[X]Phobic anxiety disorder of childhood	Anxiety
SNOMED-CT	16265951000119109	[X]Phobic state NOS	Anxiety
SNOMED-CT	16265951000119109	Post-traumatic stress disorder	Anxiety
SNOMED-CT	16265951000119109	[X]Psychic shock	Anxiety
SNOMED-CT	16265951000119109	[X]Simple phobia	Anxiety
SNOMED-CT	16265951000119109	Childhood social anxiety disorder	Anxiety
SNOMED-CT	16265951000119109	[X]Social neurosis	Anxiety
SNOMED-CT	16265951000119109	Social phobia	Anxiety
SNOMED-CT	16265951000119109	Specific phobia	Anxiety
SNOMED-CT	16265951000119109	[X]Traumatic neurosis	Anxiety

SNOMED-CT	16265951000119109	Acute post-trauma stress state	Anxiety
SNOMED-CT	16265951000119109	Anxiety disorder	Anxiety
SNOMED-CT	16265951000119109	Mixed anxiety and depressive disorder	Anxiety
SNOMED-CT	16265951000119109	Anxiousness - symptom	Anxiety
SNOMED-CT	16265951000119109	Anxious	Anxiety
SNOMED-CT	16265951000119109	Childhood and adolescent overanxiousness disturbance	Anxiety
SNOMED-CT	16265951000119109	Disturbance anxiety and fearfulness childhood/adolescent NOS	Anxiety
SNOMED-CT	16265951000119109	Disturbance of anxiety and fearfulness in childhood and adolescence	Anxiety
SNOMED-CT	16265951000119109	Phobic state	Anxiety
SNOMED-CT	16265951000119109	Agoraphobia	Anxiety
SNOMED-CT	16265951000119109	Needle phobia	Anxiety
SNOMED-CT	16265951000119109	Acute posttraumatic stress disorder following military combat	Anxiety
SNOMED-CT	16265951000119109	Chronic post-traumatic stress disorder following military combat	Anxiety
SNOMED-CT	16265951000119109	Delayed posttraumatic stress disorder following military combat	Anxiety
SNOMED-CT	16265951000119109	Panic disorder without agoraphobia	Anxiety
SNOMED-CT	16265951000119109	Anxiety about breathlessness	Anxiety
SNOMED-CT	16265951000119109	Panic disorder with agoraphobia AND severe panic attacks	Anxiety
SNOMED-CT	16265951000119109	Panic disorder with agoraphobia AND moderate panic attacks	Anxiety
SNOMED-CT	16265951000119109	Work-related stress disorder	Anxiety
SNOMED-CT	16265951000119109	Anticipatory anxiety, mild	Anxiety
SNOMED-CT	16265951000119109	Overanxious disorder of childhood	Anxiety
SNOMED-CT	16265951000119109	Generalized anxiety disorder	Anxiety
SNOMED-CT	16265951000119109	GAD - Generalized anxiety disorder	Anxiety
SNOMED-CT	16265951000119109	GAD - Generalised anxiety disorder	Anxiety
SNOMED-CT	16265951000119109	Social anxiety disorder	Anxiety
SNOMED-CT	16265951000119109	Anticipatory anxiety	Anxiety
SNOMED-CT	16265951000119109	Panic disorder with agoraphobia	Anxiety
SNOMED-CT	16265951000119109	Anticipatory anxiety, moderate	Anxiety
SNOMED-CT	16265951000119109	Cannabis-induced anxiety disorder	Anxiety
SNOMED-CT	16265951000119109	Adjustment disorder with anxious mood	Anxiety
SNOMED-CT	16265951000119109	Adjustment disorder with anxiety	Anxiety
SNOMED-CT	16265951000119109	Posttraumatic stress disorder	Anxiety
SNOMED-CT	16265951000119109	PTSD - Post-traumatic stress disorder	Anxiety
SNOMED-CT	16265951000119109	Post-traumatic stress syndrome	Anxiety
SNOMED-CT	16265951000119109	Anxiety	Anxiety
SNOMED-CT	16265951000119109	Feeling anxious	Anxiety
SNOMED-CT	16265951000119109	Anxiety reaction	Anxiety
SNOMED-CT	16265951000119109	Panic disorder without agoraphobia with panic attacks in partial remission	Anxiety
SNOMED-CT	16265951000119109	Moderate anxiety	Anxiety

SNOMED-CT	16265951000119109	Avoidant disorder of childhood	Anxiety
SNOMED-CT	16265951000119109	Childhood avoidant disorder	Anxiety
SNOMED-CT	16265951000119109	Panic disorder without agoraphobia with moderate panic attacks	Anxiety
SNOMED-CT	16265951000119109	Acute stress disorder	Anxiety
SNOMED-CT	16265951000119109	Acute crisis reaction	Anxiety
SNOMED-CT	16265951000119109	Acute reaction to stress	Anxiety
SNOMED-CT	16265951000119109	Anxiety hyperventilation	Anxiety
SNOMED-CT	16265951000119109	Phobia of going out	Anxiety
SNOMED-CT	16265951000119109	Mild anxiety	Anxiety
SNOMED-CT	16265951000119109	Panic disorder without agoraphobia with mild panic attacks	Anxiety
SNOMED-CT	16265951000119109	Panic	Anxiety
SNOMED-CT	16265951000119109	Severe anxiety (panic)	Anxiety
SNOMED-CT	16265951000119109	Free-floating anxiety	Anxiety
SNOMED-CT	16265951000119109	Shyness disorder of childhood	Anxiety
SNOMED-CT	16265951000119109	Anxiety about blushing	Anxiety
SNOMED-CT	16265951000119109	Panic disorder with agoraphobia AND mild panic attacks	Anxiety
SNOMED-CT	16265951000119109	Dream anxiety disorder	Anxiety
SNOMED-CT	16265951000119109	Nightmare disorder	Anxiety
SNOMED-CT	16265951000119109	Separation anxiety	Anxiety
SNOMED-CT	16265951000119109	Anankastic neurosis	Anxiety
SNOMED-CT	16265951000119109	OCD - Obsessive-compulsive disorder	Anxiety
SNOMED-CT	16265951000119109	Obsessive compulsive disorder	Anxiety
SNOMED-CT	16265951000119109	Transient situational disturbance	Anxiety
SNOMED-CT	16265951000119109	Childhood phobic anxiety disorder	Anxiety
SNOMED-CT	16265951000119109	Anxiety about treatment	Anxiety
SNOMED-CT	16265951000119109	Anxiety about loss of control	Anxiety
SNOMED-CT	16265951000119109	Anxiety about resuming sexual relations	Anxiety
SNOMED-CT	16265951000119109	Worried about not coping with baby	Anxiety
SNOMED-CT	16265951000119109	Worried about being a bad father	Anxiety
SNOMED-CT	16265951000119109	Worried about being a bad mother	Anxiety
SNOMED-CT	16265951000119109	Anxiety about not coping with parenthood	Anxiety
SNOMED-CT	16265951000119109	Anxiety about making mistakes	Anxiety
SNOMED-CT	16265951000119109	Anxiety about altered body image	Anxiety
SNOMED-CT	16265951000119109	Situational panic attack	Anxiety
SNOMED-CT	16265951000119109	Non-situational panic attack	Anxiety
SNOMED-CT	16265951000119109	Anxiety depression	Anxiety
SNOMED-CT	16265951000119109	Anxiety hysteria	Anxiety
SNOMED-CT	16265951000119109	Anxiety and fear	Anxiety
SNOMED-CT	16265951000119109	Anxiety about body function or health	Anxiety
SNOMED-CT	16265951000119109	Anxiety about health	Anxiety
SNOMED-CT	16265951000119109	Anxiety about behaviour or performance	Anxiety

SNOMED-CT	16265951000119109	Anxiety about social functioning	Anxiety
SNOMED-CT	16265951000119109	Anxiety about losing control of bowels	Anxiety
SNOMED-CT	16265951000119109	Anxiety about choking	Anxiety
SNOMED-CT	16265951000119109	Anxiety about swallowing	Anxiety
SNOMED-CT	16265951000119109	Anxiety about collapsing	Anxiety
SNOMED-CT	16265951000119109	Anxiety about shaking	Anxiety
SNOMED-CT	16265951000119109	Anxiety about sweating	Anxiety
SNOMED-CT	16265951000119109	Anxiety about dying	Anxiety
SNOMED-CT	16265951000119109	Anxiety about becoming fat	Anxiety
SNOMED-CT	16265951000119109	Anxiety about fainting	Anxiety
SNOMED-CT	16265951000119109	Anxiety about having a heart attack	Anxiety
SNOMED-CT	16265951000119109	Anxiety about appearing ridiculous	Anxiety
SNOMED-CT	16265951000119109	Anxiety about saying the wrong thing	Anxiety
SNOMED-CT	16265951000119109	Performance anxiety	Anxiety
SNOMED-CT	16265951000119109	Level of anxiety	Anxiety
SNOMED-CT	16265951000119109	Parental anxiety	Anxiety
SNOMED-CT	16265951000119109	Anxiety attack	Anxiety
SNOMED-CT	16265951000119109	Chronic post-traumatic stress disorder	Anxiety
SNOMED-CT	16265951000119109	Episodic paroxysmal anxiety disorder	Anxiety
SNOMED-CT	16265951000119109	Complaining of panic attack	Anxiety
SNOMED-CT	16265951000119109	Complex posttraumatic stress disorder	Anxiety
SNOMED-CT	16265951000119109	Anxiety about lethargy	Anxiety
SNOMED-CT	16265951000119109	Stranger anxiety	Anxiety
SNOMED-CT	16265951000119109	Mild major depressive disorder co-occurrent with anxiety single episode	Anxiety
SNOMED-CT	16265951000119109	Moderate major depressive disorder co-occurrent with anxiety single episode	Anxiety
SNOMED-CT	16265951000119109	Severe major depressive disorder co-occurrent with anxiety single episode	Anxiety
SNOMED-CT	16265951000119109	Recurrent mild major depressive disorder co-occurrent with anxiety	Anxiety
SNOMED-CT	16265951000119109	Recurrent severe major depressive disorder co-occurrent with anxiety	Anxiety
SNOMED-CT	16265951000119109	Recurrent moderate major depressive disorder co-occurrent with anxiety	Anxiety
SNOMED-CT	16265951000119109	Recurrent major depressive disorder co-occurrent with anxiety in full remission	Anxiety
SNOMED-CT	16265951000119109	Recurrent major depressive disorder in partial remission co-occurrent with anxiety	Anxiety
Read	Eu32900	[X]Single major depr ep, severe with psych, psych in remiss	Depression
Read	Eu32A00	[X]Recurr major depr ep, severe with psych, psych in remiss	Depression
Read	Eu34112	[X]Depressive personality disorder	Depression
Read	E135.00	Agitated depression	Depression
Read	E112.00	Single major depressive episode	Depression
Read	Eu32400	[X]Mild depression	Depression
Read	Eu32y11	[X]Atypical depression	Depression

Read	E118.00	Seasonal affective disorder	Depression
Read	Eu25100	[X]Schizoaffective disorder, depressive type	Depression
Read	Eu33212	[X]Major depression, recurrent without psychotic symptoms	Depression
Read	Eu33211	[X]Endogenous depression without psychotic symptoms	Depression
Read	Eu32000	[X]Mild depressive episode	Depression
Read	Eu41200	[X]Mixed anxiety and depressive disorder	Depression
Read	Eu32300	[X]Severe depressive episode with psychotic symptoms	Depression
Read	9H91.00	Depression medication review	Depression
Read	9H90.00	Depression annual review	Depression
Read	E113200	Recurrent major depressive episodes, moderate	Depression
Read	E113.00	Recurrent major depressive episode	Depression
Read	E112200	Single major depressive episode, moderate	Depression
Read	E112300	Single major depressive episode, severe, without psychosis	Depression
Read	Eu34114	[X]Persistant anxiety depression	Depression
Read	E112100	Single major depressive episode, mild	Depression
Read	E291.00	Prolonged depressive reaction	Depression
Read	Eu33315	[X]Recurrent severe episodes of psychotic depression	Depression
Read	E130.11	Psychotic reactive depression	Depression
Read	Eu32.12	[X]Single episode of psychogenic depression	Depression
Read	212S.00	Depression resolved	Depression
Read	Eu33.12	[X]Recurrent episodes of psychogenic depression	Depression
Read	Eu20400	[X]Post-schizophrenic depression	Depression
Read	E002100	Senile dementia with depression	Depression
Read	Eu33400	[X]Recurrent depressive disorder, currently in remission	Depression
Read	Eu32212	[X]Single episode major depression w'out psychotic symptoms	Depression
Read	Eu33311	[X]Endogenous depression with psychotic symptoms	Depression
Read	Eu32313	[X]Single episode of psychotic depression	Depression
Read	Eu32311	[X]Single episode of major depression and psychotic symptoms	Depression
Read	E113400	Recurrent major depressive episodes, severe, with psychosis	Depression
Read	E113z00	Recurrent major depressive episode NOS	Depression
Read	E11..12	Depressive psychoses	Depression
Read	E113300	Recurrent major depressive episodes, severe, no psychosis	Depression
Read	1465.00	H/O: depression	Depression
Read	E11y200	Atypical depressive disorder	Depression
Read	E001300	Presenile dementia with depression	Depression

Read	Eu32z13	[X]Prolonged single episode of reactive depression	Depression
Read	Eu33312	[X]Manic-depress psychosis,depressed type+psychotic symptoms	Depression
Read	Eu33.14	[X]Seasonal depressive disorder	Depression
Read	Eu32314	[X]Single episode of reactive depressive psychosis	Depression
Read	E113100	Recurrent major depressive episodes, mild	Depression
Read	Eu33213	[X]Manic-depress psychosis,depressd,no psychotic symptoms	Depression
Read	Eu33100	[X]Recurrent depressive disorder, current episode moderate	Depression
Read	Eu32z00	[X]Depressive episode, unspecified	Depression
Read	Eu33000	[X]Recurrent depressive disorder, current episode mild	Depression
Read	9H92.00	Depression interim review	Depression
Read	8CAa.00	Patient given advice about management of depression	Depression
Read	9k4..00	Depression - enhanced services administration	Depression
Read	Eu33314	[X]Recurr severe episodes/psychogenic depressive psychosis	Depression
Read	E112400	Single major depressive episode, severe, with psychosis	Depression
Read	E2B..00	Depressive disorder NEC	Depression
Read	8HHq.00	Referral for guided self-help for depression	Depression
Read	Eu32z12	[X]Depressive disorder NOS	Depression
Read	Eu33.00	[X]Recurrent depressive disorder	Depression
Read	Eu33313	[X]Recurr severe episodes/major depression+psychotic symptom	Depression
Read	Eu33200	[X]Recurr depress disorder cur epi severe without psyc sympt	Depression
Read	E112000	Single major depressive episode, unspecified	Depression
Read	Eu25111	[X]Schizoaffective psychosis, depressive type	Depression
Read	E113000	Recurrent major depressive episodes, unspecified	Depression
Read	Eu33z11	[X]Monopolar depression NOS	Depression
Read	Eu33316	[X]Recurrent severe episodes/reactive depressive psychosis	Depression
Read	Eu25112	[X]Schizophreniform psychosis, depressive type	Depression
Read	Eu32211	[X]Single episode agitated depressn w'out psychotic symptoms	Depression
Read	9HA0.00	On depression register	Depression
Read	E2B1.00	Chronic depression	Depression
Read	E004300	Arteriosclerotic dementia with depression	Depression
Read	E112500	Single major depressive episode, partial or unspec remission	Depression
Read	Eu33z00	[X]Recurrent depressive disorder, unspecified	Depression

Read	8BK0.00	Depression management programme	Depression
Read	Eu32.00	[X]Depressive episode	Depression
Read	Eu33300	[X]Recurrent depress disorder cur epi severe with psyc symp	Depression
Read	Eu33y00	[X]Other recurrent depressive disorders	Depression
Read	9Ov..00	Depression monitoring administration	Depression
Read	Eu32312	[X]Single episode of psychogenic depressive psychosis	Depression
Read	Eu32z11	[X]Depression NOS	Depression
Read	E113600	Recurrent major depressive episodes, in full remission	Depression
Read	E113500	Recurrent major depressive episodes,partial/unspec remission	Depression
Read	Eu32y12	[X]Single episode of masked depression NOS	Depression
Read	E112600	Single major depressive episode, in full remission	Depression
Read	E112.11	Agitated depression	Depression
Read	Eu32213	[X]Single episode vital depression w'out psychotic symptoms	Depression
Read	E112.14	Endogenous depression	Depression
Read	Eu32z14	[X] Reactive depression NOS	Depression
Read	E113700	Recurrent depression	Depression
Read	9k40.00	Depression - enhanced service completed	Depression
Read	E112.12	Endogenous depression first episode	Depression
Read	E200300	Anxiety with depression	Depression
Read	Eu32y00	[X]Other depressive episodes	Depression
Read	E113.11	Endogenous depression - recurrent	Depression
Read	E112.13	Endogenous depression first episode	Depression
Read	E112z00	Single major depressive episode NOS	Depression
Read	9Ov0.00	Depression monitoring first letter	Depression
Read	9Ov1.00	Depression monitoring second letter	Depression
Read	Eu33214	[X]Vital depression, recurrent without psychotic symptoms	Depression
Read	Eu32.13	[X]Single episode of reactive depression	Depression
Read	Eu34113	[X]Neurotic depression	Depression
Read	Eu41211	[X]Mild anxiety depression	Depression
Read	Eu34100	[X]Dysthymia	Depression
Read	E130.00	Reactive depressive psychosis	Depression
Read	Eu34111	[X]Depressive neurosis	Depression
Read	9Ov4.00	Depression monitoring telephone invite	Depression
Read	Eu33.15	[X]SAD - Seasonal affective disorder	Depression
Read	Eu33.11	[X]Recurrent episodes of depressive reaction	Depression
Read	9Ov3.00	Depression monitoring verbal invite	Depression
Read	Eu33.13	[X]Recurrent episodes of reactive depression	Depression
Read	Eu32.11	[X]Single episode of depressive reaction	Depression

Read	9Ov2.00	Depression monitoring third letter	Depression
Read	E11z200	Masked depression	Depression
Read	Eu32100	[X]Moderate depressive episode	Depression
Read	Eu32200	[X]Severe depressive episode without psychotic symptoms	Depression
Read	9kQ..00	On full dose long term treatment depression - enh serv admin	Depression
Read	Eu32600	[X]Major depression, moderately severe	Depression
Read	Eu32500	[X]Major depression, mild	Depression
Read	Eu32700	[X]Major depression, severe without psychotic symptoms	Depression
Read	Eu32800	[X]Major depression, severe with psychotic symptoms	Depression
SNOMED-CT	83458005	Agitated depression	Depression
SNOMED-CT	161469008	H/O: depression	Depression
SNOMED-CT	191455000	Presenile dementia with depression	Depression
SNOMED-CT	191459006	Senile dementia with depression	Depression
SNOMED-CT	191466007	Arteriosclerotic dementia with depression	Depression
SNOMED-CT	36923009	Single major depressive episode, unspecified	Depression
SNOMED-CT	79298009	Mild major depression, single episode	Depression
SNOMED-CT	15639000	Moderate major depression, single episode	Depression
SNOMED-CT	191604000	Single major depressive episode, severe, with psychosis	Depression
SNOMED-CT	19527009	Single episode of major depression in full remission	Depression
SNOMED-CT	36923009	Single major depressive episode NOS	Depression
SNOMED-CT	268621008	Recurrent major depressive episodes, unspecified	Depression
SNOMED-CT	191610000	Recurrent major depressive episodes, mild	Depression
SNOMED-CT	191611001	Recurrent major depressive episodes, moderate	Depression
SNOMED-CT	191613003	Recurrent major depressive episodes, severe, with psychosis	Depression
SNOMED-CT	46244001	Recurrent major depression in full remission	Depression
SNOMED-CT	191616006	Recurrent depression	Depression
SNOMED-CT	268621008	Recurrent major depressive episode NOS	Depression
SNOMED-CT	191659001	Atypical depressive disorder	Depression
SNOMED-CT	191676002	Reactive depressive psychosis	Depression
SNOMED-CT	191676002	Psychotic reactive depression	Depression
SNOMED-CT	35489007	Depressive disorder	Depression
SNOMED-CT	192080009	Chronic depression	Depression
SNOMED-CT	310495003	Mild depression	Depression
SNOMED-CT	310496002	[X]Moderate depressive episode	Depression
SNOMED-CT	310495003	[X]Recurrent depressive disorder, current episode mild	Depression

SNOMED-CT	310496002	[X]Recurrent depressive disorder, current episode moderate	Depression
SNOMED-CT	698957003	[X]Recurrent depressive disorder, currently in remission	Depression
SNOMED-CT	191616006	[X]Other recurrent depressive disorders	Depression
SNOMED-CT	231504006	[X]Other mixed anxiety disorders	Depression
SNOMED-CT	231499006	Endogenous depression first episode	Depression
SNOMED-CT	231500002	Masked depression	Depression
SNOMED-CT	247803002	Seasonal affective disorder	Depression
SNOMED-CT	36923009	Major depression, single episode	Depression
SNOMED-CT	310497006	[X]Severe depressive episode without psychotic symptoms	Depression
SNOMED-CT	191604000	[X]Severe depressive episode with psychotic symptoms	Depression
SNOMED-CT	35489007	[X]Other depressive episodes	Depression
SNOMED-CT	35489007	[X]Depressive episode, unspecified	Depression
SNOMED-CT	191616006	[X]Recurrent depressive disorder	Depression
SNOMED-CT	191616006	[X]Recurrent depressive disorder, unspecified	Depression
SNOMED-CT	274948002	Endogenous depression - recurrent	Depression
SNOMED-CT	300706003	Endogenous depression	Depression
SNOMED-CT	401174001	Depression management programme	Depression
SNOMED-CT	413169006	On depression register	Depression
SNOMED-CT	415044007	Patient given advice about management of depression	Depression
SNOMED-CT	413973005	Depression interim review	Depression
SNOMED-CT	413974004	Depression medication review	Depression
SNOMED-CT	413972000	Depression annual review	Depression
SNOMED-CT	70747007	Major depression single episode, in partial remission	Depression
SNOMED-CT	251000119105	Severe major depression, single episode	Depression
SNOMED-CT	268621008	Recurrent major depressive episodes	Depression
SNOMED-CT	764611000000100	Recurrent major depressive episodes, severe	Depression
SNOMED-CT	764691000000109	Recurrent major depressive episodes,partial/unspec remission	Depression
SNOMED-CT	192049004	Prolonged depressive adjustment reaction	Depression
SNOMED-CT	310495003	[X]Mild depression	Depression
SNOMED-CT	310497006	Severe depression	Depression
SNOMED-CT	166291000000108	Depression enhanced services administration	Depression
SNOMED-CT	166481000000107	Depression enhanced service completed	Depression
SNOMED-CT	196381000000100	Depression resolved	Depression
SNOMED-CT	199111000000100	Referral for guided self-help for depression	Depression
SNOMED-CT	87414006	Reactive depression	Depression
SNOMED-CT	191659001	[X]Atypical depression	Depression

SNOMED-CT	765176007	Psychosis and severe depression co-occurrent and due to bipolar affective disorder	Depression
SNOMED-CT	35489007	Depression	Depression
SNOMED-CT	35489007	[X]Depressive disorder NOS	Depression
SNOMED-CT	35489007	Depressive episode	Depression
SNOMED-CT	78667006	[X]Depressive neurosis	Depression
SNOMED-CT	1084061000000106	Depressive personality disorder	Depression
SNOMED-CT	78667006	Dysthymia	Depression
SNOMED-CT	73867007	[X]Endogenous depression with psychotic symptoms	Depression
SNOMED-CT	300706003	[X]Endogenous depression without psychotic symptoms	Depression
SNOMED-CT	268621008	[X]Major depression, recurrent without psychotic symptoms	Depression
SNOMED-CT	36474008	[X] Manic-depressive psychosis, depressed type without psychotic symptoms	Depression
SNOMED-CT	765176007	[X]Manic-depress psychosis,depressed type+psychotic symptoms	Depression
SNOMED-CT	231504006	[X]Mild anxiety depression	Depression
SNOMED-CT	231504006	[X]Mixed anxiety and depressive disorder	Depression
SNOMED-CT	35489007	[X]Monopolar depression NOS	Depression
SNOMED-CT	78667006	[X]Neurasthenia	Depression
SNOMED-CT	78667006	Depressive neurosis	Depression
SNOMED-CT	713831000000108	Depression monitoring administration	Depression
SNOMED-CT	717211000000107	Depression monitoring first letter	Depression
SNOMED-CT	716681000000100	Depression monitoring second letter	Depression
SNOMED-CT	716961000000102	Depression monitoring third letter	Depression
SNOMED-CT	717261000000109	Depression monitoring verbal invitation	Depression
SNOMED-CT	716421000000103	Depression monitoring telephone invitation	Depression
SNOMED-CT	231504006	[X]Persistant anxiety depression	Depression
SNOMED-CT	231485007	[X]Post-schizophrenic depression	Depression
SNOMED-CT	87414006	[X]Prolonged single episode of reactive depression	Depression
SNOMED-CT	310497006	[X]Recurr depress disorder cur epi severe without psyc sympt	Depression
SNOMED-CT	28475009	[X]Recurr severe episodes/major depression+psychotic symptom	Depression
SNOMED-CT	191613003	[X]Recurr severe episodes/psychogenic depressive psychosis	Depression
SNOMED-CT	28475009	[X]Recurrent depress disorder cur epi severe with psyc symp	Depression
SNOMED-CT	191616006	[X]Recurrent episodes of depressive reaction	Depression
SNOMED-CT	191616006	[X]Recurrent episodes of psychogenic depression	Depression
SNOMED-CT	191616006	[X]Recurrent episodes of reactive depression	Depression
SNOMED-CT	191613003	[X]Recurrent severe episodes of psychotic depression	Depression

SNOMED-CT	1086471000000103	Recurrent reactive depressive episodes, severe, with psychosis	Depression
SNOMED-CT	247803002	SAD - Seasonal affective disorder	Depression
SNOMED-CT	84760002	Schizoaffective disorder, depressive type	Depression
SNOMED-CT	84760002	[X]Schizoaffective psychosis, depressive type	Depression
SNOMED-CT	84760002	[X]Schizophreniform psychosis, depressive type	Depression
SNOMED-CT	247803002	[X]Seasonal depressive disorder	Depression
SNOMED-CT	310497006	[X] Single episode agitated depression without psychotic symptoms	Depression
SNOMED-CT	87414006	[X]Single episode of depressive reaction	Depression
SNOMED-CT	191604000	[X]Single episode of major depression and psychotic symptoms	Depression
SNOMED-CT	231500002	[X]Single episode of masked depression NOS	Depression
SNOMED-CT	87414006	[X]Single episode of psychogenic depression	Depression
SNOMED-CT	191676002	[X]Single episode of psychogenic depressive psychosis	Depression
SNOMED-CT	191604000	[X]Single episode of psychotic depression	Depression
SNOMED-CT	87414006	[X]Single episode of reactive depression	Depression
SNOMED-CT	191676002	[X]Single episode of reactive depressive psychosis	Depression
SNOMED-CT	310497006	[X]Single episode vital depression w'out psychotic symptoms	Depression
SNOMED-CT	310497006	[X]Vital depression, recurrent without psychotic symptoms	Depression
SNOMED-CT	83458005	Agitated depression	Depression
SNOMED-CT	231504006	Mixed anxiety and depressive disorder	Depression
SNOMED-CT	765176007	Bipolar affect disord, now depressed, severe with psychosis	Depression
SNOMED-CT	78667006	Depressive personality disorder	Depression
SNOMED-CT	35489007	Depressive psychoses	Depression
SNOMED-CT	231499006	Endogenous depression first episode	Depression
SNOMED-CT	87414006	Reactive depression (situational)	Depression
SNOMED-CT	78667006	Nervous exhaustion	Depression
SNOMED-CT	78667006	Neurasthenia	Depression
SNOMED-CT	361761000000106	On full dose long term treatment for depression	Depression
SNOMED-CT	87414006	Reactive (neurotic) depression	Depression
SNOMED-CT	78667006	Depressive personality	Depression
SNOMED-CT	430421000000104	Mild depression	Depression
SNOMED-CT	465441000000108	Moderate depression	Depression
SNOMED-CT	397701000000102	Severe depression	Depression
SNOMED-CT	361761000000106	On full dose long term treatment depression - enh serv admin	Depression
SNOMED-CT	832007	Moderate major depression	Depression
SNOMED-CT	73867007	Severe major depression with psychotic features	Depression

SNOMED-CT	87512008	Mild major depression	Depression
SNOMED-CT	75084000	Severe major depression without psychotic features	Depression
SNOMED-CT	755321000000106	[X]Single major depressive episode, severe, with psychosis, psychosis in remission	Depression
SNOMED-CT	755331000000108	[X]Recurrent major depressive episodes, severe, with psychosis, psychosis in remission	Depression
SNOMED-CT	28475009	Severe recurrent major depression with psychotic features	Depression
SNOMED-CT	35489007	Depressive illness	Depression
SNOMED-CT	35489007	Depressed	Depression
SNOMED-CT	36474008	Severe recurrent major depression without psychotic features	Depression
SNOMED-CT	36923009	Major depressive disorder, single episode	Depression
SNOMED-CT	40379007	Mild recurrent major depression	Depression
SNOMED-CT	73867007	Psychotic depression	Depression
SNOMED-CT	161469008	History of depressive disorder	Depression
SNOMED-CT	161469008	History of depression	Depression
SNOMED-CT	231485007	Post-schizophrenic depression	Depression
SNOMED-CT	231504006	Anxiety depression	Depression
SNOMED-CT	310496002	Moderate depression	Depression
SNOMED-CT	698957003	Depressive disorder in remission	Depression
SNOMED-CT	755321000000106	Single major depressive episode, severe, with psychosis, psychosis in remission	Depression
SNOMED-CT	755331000000108	Recurrent major depressive episodes, severe, with psychosis, psychosis in remission	Depression
SNOMED-CT	764691000000109	Recurrent major depressive episodes, in partial remission	Depression
SNOMED-CT	764711000000106	Single major depressive episode, in remission	Depression
Read	E105000	Unspecified latent schizophrenia	Psychosis
Read	E102500	Catatonic schizophrenia in remission	Psychosis
Read	E105z00	Latent schizophrenia NOS	Psychosis
Read	Eu1A500	[X]Mental behav disord due crack cocaine: psychotic disorder	Psychosis
Read	Eu14700	[X]Men & beh dis due cocaine: resid & late-onset psychot dis	Psychosis
Read	E03y000	Organic delusional syndrome	Psychosis
Read	E107z00	Schizo-affective schizophrenia NOS	Psychosis
Read	E11y000	Unspecified manic-depressive psychoses	Psychosis
Read	Eu23200	[X]Acute schizophrenia-like psychotic disorder	Psychosis
Read	Eu24.13	[X]Induced psychotic disorder	Psychosis
Read	R001400	[D]Visual hallucinations	Psychosis
Read	R001000	[D]Hallucinations, auditory	Psychosis
Read	Eu10500	[X]Mental & behav dis due to use alcohol: psychotic disorder	Psychosis

Read	E021000	Drug-induced paranoid state	Psychosis
Read	E12z.00	Paranoid psychosis NOS	Psychosis
Read	E115.11	Manic-depressive - now depressed	Psychosis
Read	E110100	Single manic episode, mild	Psychosis
Read	E120.00	Simple paranoid state	Psychosis
Read	E010.12	Delirium tremens	Psychosis
Read	E117.00	Unspecified bipolar affective disorder	Psychosis
Read	E103.00	Paranoid schizophrenia	Psychosis
Read	E122.00	Paraphrenia	Psychosis
Read	E133.00	Acute paranoid reaction	Psychosis
Read	E00y.00	Other senile and presenile organic psychoses	Psychosis
Read	E100000	Unspecified schizophrenia	Psychosis
Read	E115000	Bipolar affective disorder, currently depressed, unspecified	Psychosis
Read	E010.00	Alcohol withdrawal delirium	Psychosis
Read	E13y.00	Other reactive psychoses	Psychosis
Read	E114300	Bipolar affect disord, currently manic, severe, no psychosis	Psychosis
Read	E1y..00	Other specified non-organic psychoses	Psychosis
Read	Eu20000	[X]Paranoid schizophrenia	Psychosis
Read	E031.00	Subacute confusional state	Psychosis
Read	Eu10411	[X]Delirium tremens, alcohol induced	Psychosis
Read	E114.11	Manic-depressive - now manic	Psychosis
Read	Eu10514	[X]Alcoholic psychosis NOS	Psychosis
Read	E130.11	Psychotic reactive depression	Psychosis
Read	1BH..11	Delusion	Psychosis
Read	Eu20y13	[X]Schizophrenifrm psychos NOS	Psychosis
Read	E002000	Senile dementia with paranoia	Psychosis
Read	E110.11	Hypomanic psychoses	Psychosis
Read	1BH..00	Delusions	Psychosis
Read	R001z00	[D]Hallucinations NOS	Psychosis
Read	E111000	Recurrent manic episodes, unspecified	Psychosis
Read	E021100	Drug-induced hallucinosis	Psychosis
Read	E110000	Single manic episode, unspecified	Psychosis
Read	Eu20211	[X]Catatonic stupor	Psychosis
Read	Eu20400	[X]Post-schizophrenic depression	Psychosis
Read	E03y300	Unspecified puerperal psychosis	Psychosis
Read	E107.00	Schizo-affective schizophrenia	Psychosis
Read	Eu23012	[X]Cycloid psychosis	Psychosis
Read	Eu23100	[X]Acute polymorphic psychot disord with symp of schizophren	Psychosis
Read	Eu04.14	[X]Acute / subacute organic reaction	Psychosis
Read	Eu05200	[X]Organic delusional [schizophrenia-like] disorder	Psychosis
Read	Eu04.15	[X]Acute / subacute psycho-organic reaction	Psychosis

Read	E13y000	Psychogenic stupor	Psychosis
Read	E1z..00	Non-organic psychosis NOS	Psychosis
Read	E010.11	DTs - delirium tremens	Psychosis
Read	E030.11	Delirium - acute organic	Psychosis
Read	1BH3.00	Paranoid ideation	Psychosis
Read	E13y100	Brief reactive psychosis	Psychosis
Read	E100100	Subchronic schizophrenia	Psychosis
Read	E04y.00	Other specified chronic organic psychoses	Psychosis
Read	E031400	Subacute confusional state, of cerebrovascular origin	Psychosis
Read	E031.11	Delirium - subacute organic	Psychosis
Read	Eu20511	[X]Chronic undifferentiated schizophrenia	Psychosis
Read	E113400	Recurrent major depressive episodes, severe, with psychosis	Psychosis
Read	E117600	Unspecified bipolar affective disorder, in full remission	Psychosis
Read	E14y000	Atypical childhood psychoses	Psychosis
Read	E134.00	Psychogenic paranoid psychosis	Psychosis
Read	Eu04.13	[X]Acute / subacute infective psychosis	Psychosis
Read	E03y200	Organic affective syndrome	Psychosis
Read	R001.00	[D]Hallucinations	Psychosis
Read	Eu19500	[X]Ment/behav dis mlti drug use/oth psyc sbs: psychotc dis	Psychosis
Read	E110200	Single manic episode, moderate	Psychosis
Read	E116100	Mixed bipolar affective disorder, mild	Psychosis
Read	Eu23.00	[X]Acute and transient psychotic disorders	Psychosis
Read	E030000	Acute confusional state, post traumatic	Psychosis
Read	Eu04.00	[X]Delirium, not induced by alcohol+other psychoactive subs	Psychosis
Read	E013.00	Alcohol withdrawal hallucinosis	Psychosis
Read	E030400	Acute confusional state, of cerebrovascular origin	Psychosis
Read	R001200	[D]Hallucinations, olfactory	Psychosis
Read	Eu05000	[X]Organic hallucinosis	Psychosis
Read	E102.00	Catatonic schizophrenia	Psychosis
Read	E02z.00	Drug psychosis NOS	Psychosis
Read	E13yz00	Other reactive psychoses NOS	Psychosis
Read	Eu23112	[X]Cycloid psychosis with symptoms of schizophrenia	Psychosis
Read	E111.00	Recurrent manic episodes	Psychosis
Read	E021z00	Drug-induced paranoia or hallucinatory state NOS	Psychosis
Read	E11y200	Atypical depressive disorder	Psychosis
Read	Eu11700	[X]Men & beh dis due opioids: resid & late-onset psychot dis	Psychosis
Read	E111200	Recurrent manic episodes, moderate	Psychosis
Read	Eu23312	[X]Psychogenic paranoid psychosis	Psychosis

Read	E115200	Bipolar affective disorder, currently depressed, moderate	Psychosis
Read	E117z00	Unspecified bipolar affective disorder, NOS	Psychosis
Read	E02yz00	Other drug psychoses NOS	Psychosis
Read	E00z.00	Senile or presenile psychoses NOS	Psychosis
Read	Eu23z12	[X]Reactive psychosis	Psychosis
Read	E02y000	Drug-induced delirium	Psychosis
Read	E131.00	Acute hysterical psychosis	Psychosis
Read	E001200	Presenile dementia with paranoia	Psychosis
Read	Eu10513	[X]Alcoholic paranoia	Psychosis
Read	E015.00	Alcoholic paranoia	Psychosis
Read	E101.00	Hebephrenic schizophrenia	Psychosis
Read	E116.00	Mixed bipolar affective disorder	Psychosis
Read	E103200	Chronic paranoid schizophrenia	Psychosis
Read	E12yz00	Other paranoid states NOS	Psychosis
Read	Eu20214	[X]Schizophrenic flexibilatis cerea	Psychosis
Read	E116000	Mixed bipolar affective disorder, unspecified	Psychosis
Read	E12y.00	Other paranoid states	Psychosis
Read	E141.11	Heller's syndrome	Psychosis
Read	Eu23z11	[X]Brief reactive psychosis NOS	Psychosis
Read	E13..00	Other nonorganic psychoses	Psychosis
Read	E112400	Single major depressive episode, severe, with psychosis	Psychosis
Read	E100.00	Simple schizophrenia	Psychosis
Read	E111400	Recurrent manic episodes, severe, with psychosis	Psychosis
Read	1BH1.00	Grandiose delusions	Psychosis
Read	E10y000	Atypical schizophrenia	Psychosis
Read	E103000	Unspecified paranoid schizophrenia	Psychosis
Read	E11zz00	Other affective psychosis NOS	Psychosis
Read	E11yz00	Other and unspecified manic-depressive psychoses NOS	Psychosis
Read	Eu23z00	[X]Acute and transient psychotic disorder, unspecified	Psychosis
Read	Eu20.00	[X]Schizophrenia	Psychosis
Read	E030.12	Toxic confusional state	Psychosis
Read	Eu20z00	[X]Schizophrenia, unspecified	Psychosis
Read	Eu16711	[X]Post hallucinogen perception disorder	Psychosis
Read	E115300	Bipolar affect disord, now depressed, severe, no psychosis	Psychosis
Read	E115100	Bipolar affective disorder, currently depressed, mild	Psychosis
Read	E114000	Bipolar affective disorder, currently manic, unspecified	Psychosis
Read	Eu20600	[X]Simple schizophrenia	Psychosis
Read	Eu20213	[X]Schizophrenic catatonia	Psychosis

Read	E114100	Bipolar affective disorder, currently manic, mild	Psychosis
Read	E103500	Paranoid schizophrenia in remission	Psychosis
Read	E110z00	Manic disorder, single episode NOS	Psychosis
Read	Eu23000	[X]Acute polymorphic psychot disord without symp of schizoph	Psychosis
Read	E0z..00	Organic psychoses NOS	Psychosis
Read	E003.00	Senile dementia with delirium	Psychosis
Read	E114.00	Bipolar affective disorder, currently manic	Psychosis
Read	E110.00	Manic disorder, single episode	Psychosis
Read	E111600	Recurrent manic episodes, in full remission	Psychosis
Read	E115z00	Bipolar affective disorder, currently depressed, NOS	Psychosis
Read	E14z.11	Childhood schizophrenia NOS	Psychosis
Read	E106.00	Residual schizophrenia	Psychosis
Read	Eu12500	[X]Mental & behav dis due to cannabinoids: psychotic disordr	Psychosis
Read	E031300	Subacute confusional state, of metabolic origin	Psychosis
Read	E121.00	Chronic paranoid psychosis	Psychosis
Read	E10y.00	Other schizophrenia	Psychosis
Read	Eu04.11	[X]Acute / subacute brain syndrome	Psychosis
Read	E04z.00	Chronic organic psychosis NOS	Psychosis
Read	E100200	Chronic schizophrenic	Psychosis
Read	E030.00	Acute confusional state	Psychosis
Read	E002z00	Senile dementia with depressive or paranoid features NOS	Psychosis
Read	E141100	Residual disintegrative psychoses	Psychosis
Read	E030z00	Acute confusional state NOS	Psychosis
Read	E11z.00	Other and unspecified affective psychoses	Psychosis
Read	E12..00	Paranoid states	Psychosis
Read	E110300	Single manic episode, severe without mention of psychosis	Psychosis
Read	Eu20100	[X]Hebephrenic schizophrenia	Psychosis
Read	1BH0.00	Delusion of persecution	Psychosis
Read	E107200	Chronic schizo-affective schizophrenia	Psychosis
Read	Eu13400	[X]Men & beh dis due seds/hypns: withdrwl state wth delirium	Psychosis
Read	Eu23300	[X]Other acute predominantly delusional psychotic disorders	Psychosis
Read	E100400	Acute exacerbation of chronic schizophrenia	Psychosis
Read	Eu23y00	[X]Other acute and transient psychotic disorders	Psychosis
Read	E011000	Korsakov's alcoholic psychosis	Psychosis
Read	E021.00	Drug-induced paranoia or hallucinatory states	Psychosis
Read	E111z00	Recurrent manic episode NOS	Psychosis
Read	E111100	Recurrent manic episodes, mild	Psychosis

Read	E114200	Bipolar affective disorder, currently manic, moderate	Psychosis
Read	E115.00	Bipolar affective disorder, currently depressed	Psychosis
Read	E03y100	Organic hallucinosis syndrome	Psychosis
Read	Eu24.12	[X]Induced paranoid disorder	Psychosis
Read	E101z00	Hebephrenic schizophrenia NOS	Psychosis
Read	Eu20y00	[X]Other schizophrenia	Psychosis
Read	E001100	Presenile dementia with delirium	Psychosis
Read	Eu14500	[X]Mental & behav dis due to use cocaine: psychotic disorder	Psychosis
Read	E10yz00	Other schizophrenia NOS	Psychosis
Read	E117000	Unspecified bipolar affective disorder, unspecified	Psychosis
Read	Eu15500	[X]Mental/behav dis oth stims inc caffeine: psychotic dis	Psychosis
Read	Eu23011	[X]Bouffee delirante	Psychosis
Read	Eu20011	[X]Paraphrenic schizophrenia	Psychosis
Read	E110400	Single manic episode, severe, with psychosis	Psychosis
Read	E030100	Acute confusional state, of infective origin	Psychosis
Read	E123.11	Folie a deux	Psychosis
Read	Eu11500	[X]Mental & behav dis due to use opioids: psychotic disorder	Psychosis
Read	Eu24.00	[X]Induced delusional disorder	Psychosis
Read	E103300	Acute exacerbation of subchronic paranoid schizophrenia	Psychosis
Read	E00y.11	Presbyophrenic psychosis	Psychosis
Read	Eu04y00	[X]Other delirium	Psychosis
Read	E14z.00	Child psychosis NOS	Psychosis
Read	E103400	Acute exacerbation of chronic paranoid schizophrenia	Psychosis
Read	Eu04100	[X]Delirium superimposed on dementia	Psychosis
Read	E100z00	Simple schizophrenia NOS	Psychosis
Read	Eu04z00	[X]Delirium, unspecified	Psychosis
Read	Eu20111	[X]Disorganised schizophrenia	Psychosis
Read	R001100	[D]Hallucinations, gustatory	Psychosis
Read	E116400	Mixed bipolar affective disorder, severe, with psychosis	Psychosis
Read	E11z000	Unspecified affective psychoses NOS	Psychosis
Read	Eu16500	[X]Mental & behav dis due to hallucinogens: psychotic disord	Psychosis
Read	E116600	Mixed bipolar affective disorder, in full remission	Psychosis
Read	E03..00	Transient organic psychoses	Psychosis
Read	E004200	Arteriosclerotic dementia with paranoia	Psychosis
Read	1BH2.00	Ideas of reference	Psychosis
Read	E031z00	Subacute confusional state NOS	Psychosis
Read	E114400	Bipolar affect disord, currently manic,severe with psychosis	Psychosis

Read	E141.00	Disintegrative psychosis	Psychosis
Read	E107500	Schizo-affective schizophrenia in remission	Psychosis
Read	E004100	Arteriosclerotic dementia with delirium	Psychosis
Read	Eu19700	[X]Men/beh dis mlt drg use/oth subs: resid/late psychot dis	Psychosis
Read	E115600	Bipolar affective disorder, now depressed, in full remission	Psychosis
Read	Eu12700	[X]Mnt/bh dis due cannabinds: resid & late-onset psychot dis	Psychosis
Read	E104.00	Acute schizophrenic episode	Psychosis
Read	E114z00	Bipolar affective disorder, currently manic, NOS	Psychosis
Read	E03z.00	Transient organic psychoses NOS	Psychosis
Read	E100300	Acute exacerbation of subchronic schizophrenia	Psychosis
Read	E100500	Schizophrenia in remission	Psychosis
Read	E102000	Unspecified catatonic schizophrenia	Psychosis
Read	E107000	Unspecified schizo-affective schizophrenia	Psychosis
Read	E111500	Recurrent manic episodes, partial or unspecified remission	Psychosis
Read	E107300	Acute exacerbation subchronic schizo-affective schizophrenia	Psychosis
Read	E114500	Bipolar affect disord,currently manic, part/unspec remission	Psychosis
Read	Eu23211	[X]Brief schizophreniform disorder	Psychosis
Read	Eu15700	[X]Mnt/bh dis oth stm inc caffne resid/late-onset psycht dis	Psychosis
Read	E03yz00	Other transient organic psychoses NOS	Psychosis
Read	Eu20300	[X]Undifferentiated schizophrenia	Psychosis
Read	E11y.00	Other and unspecified manic-depressive psychoses	Psychosis
Read	E107100	Subchronic schizo-affective schizophrenia	Psychosis
Read	E031000	Subacute confusional state, post traumatic	Psychosis
Read	Eu20200	[X]Catatonic schizophrenia	Psychosis
Read	Eu10700	[X]Men & behav dis due alcoh: resid & late-onset psychot dis	Psychosis
Read	E04..00	Other chronic organic psychoses	Psychosis
Read	Eu18400	[X]Men & beh dis vol solvents: withdrawal state wth delirium	Psychosis
Read	E123.00	Shared paranoid disorder	Psychosis
Read	E116200	Mixed bipolar affective disorder, moderate	Psychosis
Read	E116300	Mixed bipolar affective disorder, severe, without psychosis	Psychosis
Read	E107400	Acute exacerbation of chronic schizo-affective schizophrenia	Psychosis
Read	E116z00	Mixed bipolar affective disorder, NOS	Psychosis
Read	E116500	Mixed bipolar affective disorder, partial/unspec remission	Psychosis
Read	E117100	Unspecified bipolar affective disorder, mild	Psychosis

Read	E115400	Bipolar affect disord, now depressed, severe with psychosis	Psychosis
Read	E114600	Bipolar affective disorder, currently manic, full remission	Psychosis
Read	E102z00	Catatonic schizophrenia NOS	Psychosis
Read	Eu10400	[X]Men & behav dis due alcohl: withdrawl state with delirium	Psychosis
Read	R001300	[D]Hallucinations, tactile	Psychosis
Read	Eu20500	[X]Residual schizophrenia	Psychosis
Read	Eu20212	[X]Schizophrenic catalepsy	Psychosis
Read	Eu10511	[X]Alcoholic hallucinosis	Psychosis
Read	E111300	Recurrent manic episodes, severe without mention psychosis	Psychosis
Read	Eu10512	[X]Alcoholic jealousy	Psychosis
Read	Eu19400	[X]Mnt/bh dis mlti drg use/oth psy sbs: wthdr state + dlrium	Psychosis
Read	E105.00	Latent schizophrenia	Psychosis
Read	E101000	Unspecified hebephrenic schizophrenia	Psychosis
Read	E0y..00	Other specified organic psychoses	Psychosis
Read	E14yz00	Other childhood psychoses NOS	Psychosis
Read	E12y000	Paranoia querulans	Psychosis
Read	E01z.00	Alcoholic psychosis NOS	Psychosis
Read	E101500	Hebephrenic schizophrenia in remission	Psychosis
Read	E01yz00	Other alcoholic psychosis NOS	Psychosis
Read	Eu04000	[X]Delirium not superimposed on dementia, so described	Psychosis
Read	E03y.00	Other transient organic psychoses	Psychosis
Read	E117400	Unspecified bipolar affective disorder,severe with psychosis	Psychosis
Read	E117200	Unspecified bipolar affective disorder, moderate	Psychosis
Read	Eu13500	[X]Mental & behav dis due to seds/hypntcs: psychotic disordr	Psychosis
Read	E14y100	Borderline psychosis of childhood	Psychosis
Read	E031100	Subacute confusional state, of infective origin	Psychosis
Read	E110600	Single manic episode in full remission	Psychosis
Read	E11y300	Other mixed manic-depressive psychoses	Psychosis
Read	E030300	Acute confusional state, of metabolic origin	Psychosis
Read	E117500	Unspecified bipolar affect disord, partial/unspec remission	Psychosis
Read	Eu23212	[X]Brief schizophrenifrm psych	Psychosis
Read	E11y100	Atypical manic disorder	Psychosis
Read	E14y.00	Other childhood psychoses	Psychosis
Read	E115500	Bipolar affect disord, now depressed, part/unspec remission	Psychosis
Read	E100.11	Schizophrenia simplex	Psychosis
Read	E117300	Unspecified bipolar affective disorder, severe, no psychosis	Psychosis

Read	Eu04.12	[X]Acute / subacute confusional state, nonalcoholic	Psychosis
Read	E10z.00	Schizophrenia NOS	Psychosis
Read	E130.00	Reactive depressive psychosis	Psychosis
Read	E10..00	Schizophrenic disorders	Psychosis
Read	Eu20311	[X]Atypical schizophrenia	Psychosis
Read	E103z00	Paranoid schizophrenia NOS	Psychosis
Read	E10y.11	Cenesthopathic schizophrenia	Psychosis
Read	E104.11	Oneirophrenia	Psychosis
Read	Eu20y12	[X]Schizophreniform disord NOS	Psychosis
Read	E030200	Acute confusional state, of endocrine origin	Psychosis
Read	E105200	Chronic latent schizophrenia	Psychosis
Read	Eu23214	[X]Schizophrenic reaction	Psychosis
Read	E105500	Latent schizophrenia in remission	Psychosis
Read	Eu11400	[X]Men & behav dis due opioid: withdrawl state with delirium	Psychosis
Read	E101400	Acute exacerbation of chronic hebephrenic schizophrenia	Psychosis
Read	Eu18500	[X]Mental & behav dis due to vol solvents: psychotic disordr	Psychosis
Read	E107.11	Cyclic schizophrenia	Psychosis
Read	E10y100	Coenesthopathic schizophrenia	Psychosis
Read	E102100	Subchronic catatonic schizophrenia	Psychosis
SNOMED-CT	216004	Delusion of persecution	Psychosis
SNOMED-CT	2073000	Delusion	Psychosis
SNOMED-CT	4926007	Schizophrenia in remission	Psychosis
SNOMED-CT	5464005	Brief reactive psychosis	Psychosis
SNOMED-CT	8635005	Alcohol withdrawal delirium	Psychosis
SNOMED-CT	8635005	Delirium tremens	Psychosis
SNOMED-CT	16990005	Subchronic schizophrenia	Psychosis
SNOMED-CT	26025008	Residual schizophrenia	Psychosis
SNOMED-CT	26472000	Paraphrenia	Psychosis
SNOMED-CT	31658008	Chronic paranoid schizophrenia	Psychosis
SNOMED-CT	42868002	Subchronic catatonic schizophrenia	Psychosis
SNOMED-CT	63181006	Paranoid schizophrenia in remission	Psychosis
SNOMED-CT	64905009	Paranoid schizophrenia	Psychosis
SNOMED-CT	71961003	Heller's syndrome	Psychosis
SNOMED-CT	71961003	Disintegrative psychosis	Psychosis
SNOMED-CT	111483008	Catatonic schizophrenia in remission	Psychosis
SNOMED-CT	191452002	Presenile dementia with delirium	Psychosis
SNOMED-CT	191454001	Presenile dementia with paranoia	Psychosis
SNOMED-CT	191457008	Senile dementia with depressive or paranoid features	Psychosis
SNOMED-CT	191458003	Senile dementia with paranoia	Psychosis
SNOMED-CT	191457008	Senile dementia with depressive or paranoid features NOS	Psychosis
SNOMED-CT	191461002	Senile dementia with delirium	Psychosis

SNOMED-CT	191464005	Arteriosclerotic dementia with delirium	Psychosis
SNOMED-CT	191465006	Arteriosclerotic dementia with paranoia	Psychosis
SNOMED-CT	268612007	Senile or presenile psychoses NOS	Psychosis
SNOMED-CT	191476005	Alcohol withdrawal hallucinosis	Psychosis
SNOMED-CT	191478006	Alcoholic paranoia	Psychosis
SNOMED-CT	42344001	Other alcoholic psychosis	Psychosis
SNOMED-CT	42344001	Other alcoholic psychosis NOS	Psychosis
SNOMED-CT	42344001	Alcoholic psychosis NOS	Psychosis
SNOMED-CT	191484009	Drug-induced paranoia or hallucinatory states	Psychosis
SNOMED-CT	191485005	Drug-induced paranoid state	Psychosis
SNOMED-CT	191486006	Drug-induced hallucinosis	Psychosis
SNOMED-CT	191484009	Drug-induced paranoia or hallucinatory state NOS	Psychosis
SNOMED-CT	191483003	Other drug psychoses	Psychosis
SNOMED-CT	191492000	Drug-induced delirium	Psychosis
SNOMED-CT	191483003	Other drug psychoses NOS	Psychosis
SNOMED-CT	191483003	Drug psychosis NOS	Psychosis
SNOMED-CT	191499009	Transient organic psychoses	Psychosis
SNOMED-CT	191502008	Acute confusional state, of infective origin	Psychosis
SNOMED-CT	191503003	Acute confusional state, of endocrine origin	Psychosis
SNOMED-CT	191504009	Acute confusional state, of metabolic origin	Psychosis
SNOMED-CT	191505005	Acute confusional state, of cerebrovascular origin	Psychosis
SNOMED-CT	2776000	Acute confusional state NOS	Psychosis
SNOMED-CT	191507002	Subacute confusional state	Psychosis
SNOMED-CT	191509004	Subacute confusional state, of infective origin	Psychosis
SNOMED-CT	191511008	Subacute confusional state, of metabolic origin	Psychosis
SNOMED-CT	191512001	Subacute confusional state, of cerebrovascular origin	Psychosis
SNOMED-CT	191507002	Subacute confusional state NOS	Psychosis
SNOMED-CT	191499009	Other transient organic psychoses	Psychosis
SNOMED-CT	18260003	Postpartum psychosis	Psychosis
SNOMED-CT	191499009	Other transient organic psychoses NOS	Psychosis
SNOMED-CT	191499009	Transient organic psychoses NOS	Psychosis
SNOMED-CT	191447007	Other chronic organic psychoses	Psychosis
SNOMED-CT	191447007	Other specified chronic organic psychoses	Psychosis
SNOMED-CT	191447007	Chronic organic psychosis NOS	Psychosis
SNOMED-CT	191447007	Other specified organic psychoses	Psychosis
SNOMED-CT	191447007	Organic psychoses NOS	Psychosis
SNOMED-CT	191525009	Non-organic psychoses	Psychosis
SNOMED-CT	191526005	Schizophrenic disorders	Psychosis
SNOMED-CT	191527001	Schizophrenia simplex	Psychosis

SNOMED-CT	191527001	Simple schizophrenia	Psychosis
SNOMED-CT	58214004	Unspecified schizophrenia	Psychosis
SNOMED-CT	111482003	Acute exacerbation of subchronic schizophrenia	Psychosis
SNOMED-CT	191531007	Acute exacerbation of chronic schizophrenia	Psychosis
SNOMED-CT	191527001	Simple schizophrenia NOS	Psychosis
SNOMED-CT	35252006	Unspecified hebephrenic schizophrenia	Psychosis
SNOMED-CT	191539009	Acute exacerbation of chronic hebephrenic schizophrenia	Psychosis
SNOMED-CT	31373002	Hebephrenic schizophrenia in remission	Psychosis
SNOMED-CT	35252006	Hebephrenic schizophrenia NOS	Psychosis
SNOMED-CT	191542003	Catatonic schizophrenia	Psychosis
SNOMED-CT	191542003	[X]Schizophrenic flexibilatis cerea	Psychosis
SNOMED-CT	191542003	Unspecified catatonic schizophrenia	Psychosis
SNOMED-CT	191542003	Catatonic schizophrenia NOS	Psychosis
SNOMED-CT	64905009	Unspecified paranoid schizophrenia	Psychosis
SNOMED-CT	191554003	Acute exacerbation of subchronic paranoid schizophrenia	Psychosis
SNOMED-CT	191555002	Acute exacerbation of chronic paranoid schizophrenia	Psychosis
SNOMED-CT	64905009	Paranoid schizophrenia NOS	Psychosis
SNOMED-CT	191559008	Latent schizophrenia	Psychosis
SNOMED-CT	191559008	Unspecified latent schizophrenia	Psychosis
SNOMED-CT	191562006	Chronic latent schizophrenia	Psychosis
SNOMED-CT	191565008	Latent schizophrenia in remission	Psychosis
SNOMED-CT	191559008	Latent schizophrenia NOS	Psychosis
SNOMED-CT	191567000	Cyclic schizophrenia	Psychosis
SNOMED-CT	191577003	Coenesthopathic schizophrenia	Psychosis
SNOMED-CT	191577003	Cenesthopathic schizophrenia	Psychosis
SNOMED-CT	58214004	Other schizophrenia NOS	Psychosis
SNOMED-CT	58214004	Schizophrenia	Psychosis
SNOMED-CT	268619003	Single manic episode, unspecified	Psychosis
SNOMED-CT	191583000	Single manic episode, mild	Psychosis
SNOMED-CT	191584006	Single manic episode, moderate	Psychosis
SNOMED-CT	764641000000104	Single manic episode, severe	Psychosis
SNOMED-CT	191586008	Single manic episode, severe, with psychosis	Psychosis
SNOMED-CT	191588009	Single manic episode in full remission	Psychosis
SNOMED-CT	268619003	Manic disorder, single episode NOS	Psychosis
SNOMED-CT	191590005	Recurrent manic episodes	Psychosis
SNOMED-CT	191590005	Recurrent manic episodes, unspecified	Psychosis
SNOMED-CT	191592002	Recurrent manic episodes, mild	Psychosis
SNOMED-CT	191593007	Recurrent manic episodes, moderate	Psychosis
SNOMED-CT	191595000	Recurrent manic episodes, severe, with psychosis	Psychosis
SNOMED-CT	191597008	Recurrent manic episodes, in full remission	Psychosis

SNOMED-CT	191590005	Recurrent manic episode NOS	Psychosis
SNOMED-CT	191604000	Single major depressive episode, severe, with psychosis	Psychosis
SNOMED-CT	191613003	Recurrent major depressive episodes, severe, with psychosis	Psychosis
SNOMED-CT	191618007	Manic-depressive - now manic	Psychosis
SNOMED-CT	191618007	Bipolar affective disorder, currently manic, unspecified	Psychosis
SNOMED-CT	191620005	Bipolar affective disorder, currently manic, mild	Psychosis
SNOMED-CT	191621009	Bipolar affective disorder, currently manic, moderate	Psychosis
SNOMED-CT	191618007	Bipolar affective disorder, currently manic, NOS	Psychosis
SNOMED-CT	191627008	Manic-depressive - now depressed	Psychosis
SNOMED-CT	191627008	Bipolar affective disorder, currently depressed, unspecified	Psychosis
SNOMED-CT	191629006	Bipolar affective disorder, currently depressed, mild	Psychosis
SNOMED-CT	191630001	Bipolar affective disorder, currently depressed, moderate	Psychosis
SNOMED-CT	191627008	Bipolar affective disorder, currently depressed, NOS	Psychosis
SNOMED-CT	191636007	Mixed bipolar affective disorder	Psychosis
SNOMED-CT	191636007	Mixed bipolar affective disorder, unspecified	Psychosis
SNOMED-CT	191638008	Mixed bipolar affective disorder, mild	Psychosis
SNOMED-CT	191639000	Mixed bipolar affective disorder, moderate	Psychosis
SNOMED-CT	191641004	Mixed bipolar affective disorder, severe, with psychosis	Psychosis
SNOMED-CT	191643001	Mixed bipolar affective disorder, in full remission	Psychosis
SNOMED-CT	191636007	Mixed bipolar affective disorder, NOS	Psychosis
SNOMED-CT	13746004	Unspecified bipolar affective disorder	Psychosis
SNOMED-CT	13746004	Unspecified bipolar affective disorder, unspecified	Psychosis
SNOMED-CT	13313007	Mild bipolar disorder	Psychosis
SNOMED-CT	79584002	Moderate bipolar disorder	Psychosis
SNOMED-CT	41836007	Bipolar disorder in full remission	Psychosis
SNOMED-CT	13746004	Unspecified bipolar affective disorder, NOS	Psychosis
SNOMED-CT	13746004	Other and unspecified manic-depressive psychoses	Psychosis
SNOMED-CT	13746004	Unspecified manic-depressive psychoses	Psychosis
SNOMED-CT	191658009	Atypical manic disorder	Psychosis
SNOMED-CT	191659001	Atypical depressive disorder	Psychosis
SNOMED-CT	16506000	Mixed bipolar I disorder	Psychosis
SNOMED-CT	13746004	Other and unspecified manic-depressive psychoses NOS	Psychosis
SNOMED-CT	441704009	Affective psychosis	Psychosis
SNOMED-CT	441704009	Unspecified affective psychoses NOS	Psychosis
SNOMED-CT	441704009	Other affective psychosis NOS	Psychosis

SNOMED-CT	191668004	Simple paranoid state	Psychosis
SNOMED-CT	191670008	Folie a deux	Psychosis
SNOMED-CT	191670008	Shared paranoid disorder	Psychosis
SNOMED-CT	191667009	Other paranoid states	Psychosis
SNOMED-CT	191672000	Paranoia querulans	Psychosis
SNOMED-CT	191667009	Other paranoid states NOS	Psychosis
SNOMED-CT	191667009	Paranoid psychosis	Psychosis
SNOMED-CT	191676002	Reactive depressive psychosis	Psychosis
SNOMED-CT	191676002	Psychotic reactive depression	Psychosis
SNOMED-CT	191677006	Acute hysterical psychosis	Psychosis
SNOMED-CT	191680007	Psychogenic paranoid psychosis	Psychosis
SNOMED-CT	231437006	Other reactive psychoses	Psychosis
SNOMED-CT	191683009	Psychogenic stupor	Psychosis
SNOMED-CT	231437006	Other reactive psychoses NOS	Psychosis
SNOMED-CT	191687005	Psychoses with origin in childhood	Psychosis
SNOMED-CT	191693002	Residual disintegrative psychoses	Psychosis
SNOMED-CT	71961003	Disintegrative psychosis NOS	Psychosis
SNOMED-CT	191687005	Other childhood psychoses	Psychosis
SNOMED-CT	191696005	Atypical childhood psychoses	Psychosis
SNOMED-CT	191697001	Borderline psychosis of childhood	Psychosis
SNOMED-CT	191687005	Other childhood psychoses NOS	Psychosis
SNOMED-CT	191525009	Other specified non-organic psychoses	Psychosis
SNOMED-CT	111479008	[X]Organic, including symptomatic, mental disorders	Psychosis
SNOMED-CT	2776000	[X]Delirium not superimposed on dementia, so described	Psychosis
SNOMED-CT	2776000	[X]Delirium superimposed on dementia	Psychosis
SNOMED-CT	2776000	Delirium	Psychosis
SNOMED-CT	46975003	Cocaine-induced organic mental disorder	Psychosis
SNOMED-CT	111484002	Undifferentiated schizophrenia	Psychosis
SNOMED-CT	58214004	[X]Schizophrenia, unspecified	Psychosis
SNOMED-CT	231489001	[X]Other acute and transient psychotic disorders	Psychosis
SNOMED-CT	85248005	Bipolar disorder in remission	Psychosis
SNOMED-CT	13746004	[X]Bipolar affective disorder, unspecified	Psychosis
SNOMED-CT	386782008	Disturbance in affect	Psychosis
SNOMED-CT	386782008	[X]Other persistent mood affective disorders	Psychosis
SNOMED-CT	386782008	[X]Persistent mood affective disorder, unspecified	Psychosis
SNOMED-CT	386810004	[X]Phobic anxiety disorders	Psychosis
SNOMED-CT	386810004	[X]Other phobic anxiety disorders	Psychosis
SNOMED-CT	386810004	[X]Phobic anxiety disorder, unspecified	Psychosis
SNOMED-CT	7011001	[D]Hallucinations	Psychosis
SNOMED-CT	45150006	Auditory hallucinations	Psychosis
SNOMED-CT	29139005	Gustatory hallucinations	Psychosis

SNOMED-CT	39672001	Olfactory hallucinations	Psychosis
SNOMED-CT	66609003	Tactile hallucinations	Psychosis
SNOMED-CT	64269007	[D]Visual hallucinations	Psychosis
SNOMED-CT	7011001	[D]Hallucinations NOS	Psychosis
SNOMED-CT	231439009	[D]Toxic confusional state	Psychosis
SNOMED-CT	7011001	[X]Other hallucinations	Psychosis
SNOMED-CT	231437006	Reactive psychoses	Psychosis
SNOMED-CT	231438001	Presbyophrenic psychosis	Psychosis
SNOMED-CT	231439009	Toxic confusional state	Psychosis
SNOMED-CT	191687005	Childhood schizophrenia NOS	Psychosis
SNOMED-CT	247667002	Grandiose delusions	Psychosis
SNOMED-CT	268612007	Senile and presenile organic psychotic conditions	Psychosis
SNOMED-CT	268612007	Other senile and presenile organic psychoses	Psychosis
SNOMED-CT	268617001	Acute schizophrenic episode	Psychosis
SNOMED-CT	58214004	Other schizophrenia	Psychosis
SNOMED-CT	268619003	Manic disorder, single episode	Psychosis
SNOMED-CT	268622001	Chronic paranoid psychosis	Psychosis
SNOMED-CT	268624000	Acute paranoid reaction	Psychosis
SNOMED-CT	191525009	Non-organic psychosis NOS	Psychosis
SNOMED-CT	191687005	Child psychosis NOS	Psychosis
SNOMED-CT	386810004	Phobic disorder	Psychosis
SNOMED-CT	2776000	[X]Other delirium	Psychosis
SNOMED-CT	111479008	Organic mental disorder	Psychosis
SNOMED-CT	58214004	[X]Other schizophrenia	Psychosis
SNOMED-CT	231489001	[X]Other acute predominantly delusional psychotic disorders	Psychosis
SNOMED-CT	231489001	[X]Acute and transient psychotic disorder, unspecified	Psychosis
SNOMED-CT	191525009	[X]Unspecified nonorganic psychosis	Psychosis
SNOMED-CT	268619003	[X]Manic episode, unspecified	Psychosis
SNOMED-CT	13746004	[X]Other bipolar affective disorders	Psychosis
SNOMED-CT	191604000	[X]Severe depressive episode with psychotic symptoms	Psychosis
SNOMED-CT	386782008	[X]Other recurrent mood affective disorders	Psychosis
SNOMED-CT	2073000	Delusions	Psychosis
SNOMED-CT	2776000	Acute confusional state	Psychosis
SNOMED-CT	41189006	Ideas of reference	Psychosis
SNOMED-CT	64269007	Visual hallucinations	Psychosis
SNOMED-CT	69482004	Wernicke-Korsakov syndrome	Psychosis
SNOMED-CT	7011001	Hallucinations	Psychosis
SNOMED-CT	8635005	DTs - delirium tremens	Psychosis
SNOMED-CT	111484002	Atypical schizophrenia	Psychosis
SNOMED-CT	83746006	Chronic schizophrenic	Psychosis
SNOMED-CT	111479008	[X]Organic psychosyndrome	Psychosis

SNOMED-CT	417233008	Paranoid ideation	Psychosis
SNOMED-CT	762325009	Psychotic disorder caused by stimulant	Psychosis
SNOMED-CT	111479008	Other specific mental disorder post-organic brain damage	Psychosis
SNOMED-CT	191525009	Other nonorganic psychoses	Psychosis
SNOMED-CT	23645006	Organic affective syndrome	Psychosis
SNOMED-CT	5510009	Organic delusional syndrome	Psychosis
SNOMED-CT	45912004	Organic hallucinosis syndrome	Psychosis
SNOMED-CT	191447007	Organic psychotic condition	Psychosis
SNOMED-CT	191567000	Unspecified schizo-affective schizophrenia	Psychosis
SNOMED-CT	5703000	Bipolar disorder in partial remission	Psychosis
SNOMED-CT	53049002	Severe bipolar disorder without psychotic features	Psychosis
SNOMED-CT	4441000	Severe bipolar disorder with psychotic features	Psychosis
SNOMED-CT	191508007	Subacute confusional state, post traumatic	Psychosis
SNOMED-CT	191569002	Subchronic schizo-affective schizophrenia	Psychosis
SNOMED-CT	191567000	Schizoaffective schizophrenia	Psychosis
SNOMED-CT	191574005	Schizoaffective schizophrenia in remission	Psychosis
SNOMED-CT	191567000	Schizo-affective schizophrenia NOS	Psychosis
SNOMED-CT	268622001	Sander's disease	Psychosis
SNOMED-CT	26025008	Restzustand - schizophrenia	Psychosis
SNOMED-CT	764671000000105	Recurrent manic episodes, in partial remission	Psychosis
SNOMED-CT	764621000000106	Recurrent manic episodes, severe	Psychosis
SNOMED-CT	48500005	Delusional disorder	Psychosis
SNOMED-CT	191667009	[X]Paranoid psychosis	Psychosis
SNOMED-CT	417233008	[X]Paranoia	Psychosis
SNOMED-CT	191525009	Non-organic psychosis	Psychosis
SNOMED-CT	386810004	Phobic disorders	Psychosis
SNOMED-CT	386810004	Phobic anxiety disorder	Psychosis
SNOMED-CT	191667009	Paranoid disorder	Psychosis
SNOMED-CT	268612007	[X] Presenile psychosis NOS	Psychosis
SNOMED-CT	191457008	[X] Senile dementia, depressed or paranoid type	Psychosis
SNOMED-CT	268612007	[X] Senile psychosis NOS	Psychosis
SNOMED-CT	2776000	[X]Acute / subacute brain syndrome	Psychosis
SNOMED-CT	111479008	[X]Acute / subacute confusional state, nonalcoholic	Psychosis
SNOMED-CT	191502008	[X]Acute / subacute infective psychosis	Psychosis
SNOMED-CT	2776000	Acute organic reaction	Psychosis
SNOMED-CT	111479008	[X]Acute / subacute psycho-organic reaction	Psychosis
SNOMED-CT	231489001	Acute transient psychotic disorder	Psychosis
SNOMED-CT	712850003	Acute polymorphic psychotic disorder co-occurrent with symptoms of schizophrenia	Psychosis

SNOMED-CT	712824002	Acute polymorphic psychotic disorder without symptoms of schizophrenia	Psychosis
SNOMED-CT	278853003	Acute schizophrenia-like psychotic disorder	Psychosis
SNOMED-CT	7052005	Alcoholic hallucinosis	Psychosis
SNOMED-CT	42344001	[X]Alcoholic jealousy	Psychosis
SNOMED-CT	42344001	[X]Alcoholic paranoia	Psychosis
SNOMED-CT	42344001	[X]Alcoholic psychosis NOS	Psychosis
SNOMED-CT	191659001	[X]Atypical depression	Psychosis
SNOMED-CT	111484002	[X]Atypical schizophrenia	Psychosis
SNOMED-CT	61403008	Severe depressed bipolar I disorder without psychotic features	Psychosis
SNOMED-CT	765176007	Psychosis and severe depression co-occurrent and due to bipolar affective disorder	Psychosis
SNOMED-CT	191623007	Bipolar affective disorder, currently manic, severe, with psychosis	Psychosis
SNOMED-CT	191618007	[X]Bipolar affect disorder cur epi manic wout psychotic symp	Psychosis
SNOMED-CT	191630001	[X]Bipolar affect disorder cur epi mild or moderate depressn	Psychosis
SNOMED-CT	13746004	Bipolar affective disorder	Psychosis
SNOMED-CT	192362008	[X]Bipolar affective disorder, current episode mixed	Psychosis
SNOMED-CT	268619003	[X]Bipolar disorder, single manic episode	Psychosis
SNOMED-CT	274952002	Borderline schizophrenia	Psychosis
SNOMED-CT	63204009	[X]Bouffee delirante	Psychosis
SNOMED-CT	712850003	[X]Bouffee delirante with symptoms of schizophrenia	Psychosis
SNOMED-CT	231489001	[X]Brief reactive psychosis NOS	Psychosis
SNOMED-CT	278853003	[X]Brief schizophreniform disorder	Psychosis
SNOMED-CT	278853003	[X]Brief schizophrenifrm psych	Psychosis
SNOMED-CT	191542003	[X]Catatonic schizophrenia	Psychosis
SNOMED-CT	191542003	[X]Catatonic stupor	Psychosis
SNOMED-CT	58214004	[X]Cenesthopathic schizophrenia	Psychosis
SNOMED-CT	111484002	[X]Chronic undifferentiated schizophrenia	Psychosis
SNOMED-CT	307417003	[X]Cycloid psychosis	Psychosis
SNOMED-CT	307417003	Cycloid psychosis	Psychosis
SNOMED-CT	2776000	[X]Delirium of mixed origin	Psychosis
SNOMED-CT	8635005	[X]Delirium tremens, alcohol induced	Psychosis
SNOMED-CT	111479008	[X]Delirium, not induced by alcohol+other psychoactive subs	Psychosis
SNOMED-CT	71961003	[X]Dementia infantalis	Psychosis
SNOMED-CT	35252006	[X]Disorganised schizophrenia	Psychosis
SNOMED-CT	111479008	[X]Epileptic psychosis NOS	Psychosis
SNOMED-CT	191670008	[X]Folie a deux	Psychosis
SNOMED-CT	35252006	[X]Hebephrenic schizophrenia	Psychosis
SNOMED-CT	71961003	[X]Heller's syndrome	Psychosis
SNOMED-CT	231496004	Hypomania	Psychosis

SNOMED-CT	61831009	Induced delusional disorder	Psychosis
SNOMED-CT	61831009	[X]Induced paranoid disorder	Psychosis
SNOMED-CT	61831009	[X]Induced psychotic disorder	Psychosis
SNOMED-CT	69482004	Korsakoff's psychosis	Psychosis
SNOMED-CT	191559008	[X]Latent schizophrenia	Psychosis
SNOMED-CT	191559008	[X]Latent schizophrenic reaction	Psychosis
SNOMED-CT	13746004	[X]Manic-depressive reaction	Psychosis
SNOMED-CT	268619003	[X]Mania NOS	Psychosis
SNOMED-CT	268619003	[X]Manic episode	Psychosis
SNOMED-CT	765176007	[X]Manic-depress psychosis,depressed type+psychotic symptoms	Psychosis
SNOMED-CT	13746004	Manic-depressive illness	Psychosis
SNOMED-CT	13746004	Manic-depressive psychosis	Psychosis
SNOMED-CT	91388009	[X]Men & beh dis due cocaine: resid & late-onset psychot dis	Psychosis
SNOMED-CT	91388009	[X]Mental and behavioural disorders due to use of hallucinogens: withdrawal state with delirium	Psychosis
SNOMED-CT	943071000000104	[X]Men & beh dis due opioids: resid & late-onset psychot dis	Psychosis
SNOMED-CT	91388009	[X]Men & beh dis due seds/hypns: withdrwl state wth delirium	Psychosis
SNOMED-CT	74934004	[X]Men & beh dis vol solvents: withdrawal state wth delirium	Psychosis
SNOMED-CT	42344001	[X]Men & behav dis due alcoh: resid & late-onset psychot dis	Psychosis
SNOMED-CT	8635005	[X]Men & behav dis due alcohl: withdrawl state with delirium	Psychosis
SNOMED-CT	91388009	[X]Men & behav dis due cocaine: withdrawl state wth delirium	Psychosis
SNOMED-CT	91388009	[X]Men & behav dis due opioid: withdrawl state with delirium	Psychosis
SNOMED-CT	46975003	[X]Men & behav dis due to use cocaine: oth men & behav dis	Psychosis
SNOMED-CT	91388009	[X]Men/beh dis mlt drg use/oth psy sbs: oth men & behav dis	Psychosis
SNOMED-CT	91388009	[X]Men/beh dis mlt drg use/oth subs: resid/late psychot dis	Psychosis
SNOMED-CT	91388009	[X]Men/behav dis due to use hallucinogens: oth men/behav dis	Psychosis
SNOMED-CT	91388009	[X]Men/behav dis due to use seds/hypntcs: oth men/behav disd	Psychosis
SNOMED-CT	91388009	[X]Men/behav dis due to use vol solvents: oth men/behav disd	Psychosis
SNOMED-CT	91388009	[X]Ment & behav dis due use alcohol: unsp ment & behav dis	Psychosis
SNOMED-CT	91388009	[X]Ment & behav dis due use cocaine: unsp ment & behav dis	Psychosis
SNOMED-CT	91388009	[X]Ment & behav dis due use opioids: unsp ment & behav dis	Psychosis
SNOMED-CT	91388009	Psychoactive substance abuse	Psychosis
SNOMED-CT	91388009	[X]Ment/behav dis due to use oth stims inc caff: harmful use	Psychosis
SNOMED-CT	91388009	[X]Ment/behav dis due use hallucinogens: unsp ment/behav dis	Psychosis

SNOMED-CT	91388009	[X]Ment/behav dis due use seds/hypntcs: unsp ment/behav disd	Psychosis
SNOMED-CT	91388009	[X]Ment/behav dis due use vol solvents: unsp ment/behav dis	Psychosis
SNOMED-CT	191483003	[X]Mental and behavioural disorders due to multiple drug use and use of other psychoactive substances: psychotic disorder	Psychosis
SNOMED-CT	91388009	[X]Mental & behav dis due hallucinogens: acute intoxicatn	Psychosis
SNOMED-CT	91388009	[X]Mental & behav dis due seds/hypntcs: acute intoxication	Psychosis
SNOMED-CT	943081000000102	Cannabis-induced psychosis	Psychosis
SNOMED-CT	943131000000102	[X]Mental and behavioural disorders due to use of hallucinogens: psychotic disorder	Psychosis
SNOMED-CT	943091000000100	[X]Mental and behavioural disorders due to use of sedatives or hypnotics: psychotic disorder	Psychosis
SNOMED-CT	42344001	Alcohol-induced psychosis	Psychosis
SNOMED-CT	91388009	[X]Mental & behav dis due to use cocaine: acute intoxication	Psychosis
SNOMED-CT	943101000000108	Cocaine-induced psychosis	Psychosis
SNOMED-CT	943071000000104	Opioid-induced psychosis	Psychosis
SNOMED-CT	943151000000109	Volatile inhalant-induced psychosis	Psychosis
SNOMED-CT	91388009	[X]Mental and behavioural disorders due to use of other stimulants, including caffeine	Psychosis
SNOMED-CT	91388009	[X]Mental and behavioural disorders due to use of hallucinogens: withdrawal state	Psychosis
SNOMED-CT	91388009	[X]Mental and behav dis due seds/hypntcs: withdrawal state	Psychosis
SNOMED-CT	91388009	[X]Mental and behav dis due to use cannabinoids: amnesic syn	Psychosis
SNOMED-CT	91388009	[X]Mental and behav dis due to use cocaine: amnesic syndrome	Psychosis
SNOMED-CT	91388009	[X]Mental and behav dis due to use cocaine: withdrawal state	Psychosis
SNOMED-CT	91388009	[X]Mental and behav dis due to use hallucinogens: harmfl use	Psychosis
SNOMED-CT	91388009	[X]Mental and behav dis due to use opioids: amnesic syndrome	Psychosis
SNOMED-CT	91388009	[X]Mental and behav dis due to use seds/hypntcs: amnesic syn	Psychosis
SNOMED-CT	91388009	[X]Mental and behav dis due to use seds/hypntcs: harmful use	Psychosis
SNOMED-CT	91388009	[X]Mental and behav dis due to use vol solvents: amnesic syn	Psychosis
SNOMED-CT	91388009	[X]Mental and behav dis due to vol solvents: dependence synd	Psychosis
SNOMED-CT	91388009	[X]Mental and behav dis due use hallucinogens: amnesic syndr	Psychosis
SNOMED-CT	74934004	[X]Mental and behav dis due vol solvents: withdrawal state	Psychosis
SNOMED-CT	91388009	[X]Mental and behav dis due volatile solvents: harmful use	Psychosis

SNOMED-CT	91388009	[X]Mental and behav dis mlti/oth psych sbs: dependence syndr	Psychosis
SNOMED-CT	91388009	[X]Mental and behav dis mlti/oth psychoa sbs: withdrwl state	Psychosis
SNOMED-CT	91388009	[X]Mental and behav dis oth stim inc caffein: dependnce synd	Psychosis
SNOMED-CT	91388009	[X]Mental and behav dis oth stims inc caffeine: amnesic syn	Psychosis
SNOMED-CT	91388009	[X]Mental and behavioural dis due use sedatives/hypnotics	Psychosis
SNOMED-CT	91388009	[X]Mental and behavioural disorders due to use hallucinogens	Psychosis
SNOMED-CT	91388009	[X]Mental/behav dis multi drg use/psychoac subs: acute intox	Psychosis
SNOMED-CT	762325009	[X]Mental and behavioural disorders due to use of other stimulants, including caffeine: psychotic disorder	Psychosis
SNOMED-CT	192362008	Bipolar affective disorder , current episode mixed	Psychosis
SNOMED-CT	91388009	[X]Mnt/behav dis other stimlnts inc caffeine: withdrwl state	Psychosis
SNOMED-CT	91388009	[X]Mnt/bh dis due cannabinds: resid & late-onset psychot dis	Psychosis
SNOMED-CT	91388009	[X]Mnt/bh dis due hallucngns: resid & late-onset psychot dis	Psychosis
SNOMED-CT	91388009	[X]Mnt/bh dis due seds/hypns: resid & late-onset psychot dis	Psychosis
SNOMED-CT	91388009	[X]Mnt/bh dis mlti drg use/oth psy sbs: wthdr state + dlrium	Psychosis
SNOMED-CT	91388009	[X]Mnt/bh dis oth stims inc caffne: withdr state wt delirium	Psychosis
SNOMED-CT	762325009	[X]Mental and behavioural disorders due to use of other stimulants, including caffeine: residual and late onset psychotic disorder	Psychosis
SNOMED-CT	91388009	[X]Mnt/bh dis vol solvents: resid & late-onset psychotic dis	Psychosis
SNOMED-CT	278853003	[X]Oneirophrenia	Psychosis
SNOMED-CT	111479008	[X]Organic brain syndrome NOS	Psychosis
SNOMED-CT	5510009	Organic delusional disorder	Psychosis
SNOMED-CT	45912004	Organic hallucinosis	Psychosis
SNOMED-CT	111479008	[X]Organic mental disorder NOS	Psychosis
SNOMED-CT	23645006	Organic mood disorder	Psychosis
SNOMED-CT	111479008	[X]Organic psychosis NOS	Psychosis
SNOMED-CT	111479008	[X]Oth mental disorder brain damag/dysfunction/physical disr	Psychosis
SNOMED-CT	111479008	[X]Oth organ personality behav disorders brain dis dam dysf	Psychosis
SNOMED-CT	111479008	[X]Oth sp mental disord brain damag/dysfunction/physcal disd	Psychosis
SNOMED-CT	191525009	[X]Other nonorganic psychotic disorders	Psychosis
SNOMED-CT	5510009	[X]Paranoid organic state	Psychosis
SNOMED-CT	64905009	[X]Paranoid schizophrenia	Psychosis
SNOMED-CT	191667009	[X]Paranoid state	Psychosis

SNOMED-CT	64905009	[X]Paraphrenic schizophrenia	Psychosis
SNOMED-CT	111479008	[X]Personality and behav disorder brain dis dam and dysfunct	Psychosis
SNOMED-CT	386810004	Phobic disorder	Psychosis
SNOMED-CT	386810004	[X]Phobic state NOS	Psychosis
SNOMED-CT	91388009	[X]Post hallucinogen perception disorder	Psychosis
SNOMED-CT	231485007	[X]Post-schizophrenic depression	Psychosis
SNOMED-CT	191680007	[X]Psychogenic paranoid psychosis	Psychosis
SNOMED-CT	231437006	[X]Reactive psychosis	Psychosis
SNOMED-CT	191613003	[X]Recurr severe episodes/psychogenic depressive psychosis	Psychosis
SNOMED-CT	191590005	[X]Recurrent manic episodes	Psychosis
SNOMED-CT	191613003	[X]Recurrent severe episodes of psychotic depression	Psychosis
SNOMED-CT	26025008	[X]Residual schizophrenia	Psychosis
SNOMED-CT	26025008	[X]Restzustand schizophrenic	Psychosis
SNOMED-CT	58214004	[X]Schizophrenia	Psychosis
SNOMED-CT	5510009	[X]Schizophrenia-like psychosis in epilepsy	Psychosis
SNOMED-CT	191542003	[X]Schizophrenic catalepsy	Psychosis
SNOMED-CT	191542003	[X]Schizophrenic catatonia	Psychosis
SNOMED-CT	278853003	[X]Schizophrenic reaction	Psychosis
SNOMED-CT	58214004	[X]Schizophreniform disord NOS	Psychosis
SNOMED-CT	58214004	[X]Schizophrenifrm psychos NOS	Psychosis
SNOMED-CT	41189006	[X]Sensitiver Beziehungswahn	Psychosis
SNOMED-CT	386810004	[X]Simple phobia	Psychosis
SNOMED-CT	191527001	[X]Simple schizophrenia	Psychosis
SNOMED-CT	191604000	[X]Single episode of major depression and psychotic symptoms	Psychosis
SNOMED-CT	191676002	[X]Single episode of psychogenic depressive psychosis	Psychosis
SNOMED-CT	191604000	[X]Single episode of psychotic depression	Psychosis
SNOMED-CT	191676002	[X]Single episode of reactive depressive psychosis	Psychosis
SNOMED-CT	111479008	[X]Unspec mental disorder brain damag/dysfunction/physcal dr	Psychosis
SNOMED-CT	111479008	[X]Unspec organ personality behav disorder brain dam dysfunc	Psychosis
SNOMED-CT	191501001	Acute confusional state, post traumatic	Psychosis
SNOMED-CT	191572009	Acute exacerbation of chronic schizo-affective schizophrenia	Psychosis
SNOMED-CT	191571002	Acute exacerbation subchronic schizo-affective schizophrenia	Psychosis
SNOMED-CT	69482004	Alcohol amnestic syndrome	Psychosis
SNOMED-CT	42344001	Alcohol-induced psychosis	Psychosis
SNOMED-CT	162004	Severe manic bipolar I disorder without psychotic features	Psychosis
SNOMED-CT	191623007	Bipolar affect disord, currently manic,severe with psychosis	Psychosis

SNOMED-CT	49512000	Depressed bipolar I disorder in partial remission	Psychosis
SNOMED-CT	765176007	Bipolar affect disord, now depressed, severe with psychosis	Psychosis
SNOMED-CT	61403008	Bipolar affect disord, now depressed, severe, no psychosis	Psychosis
SNOMED-CT	63249007	Manic bipolar I disorder in partial remission	Psychosis
SNOMED-CT	191627008	Bipolar affective disorder, current episode depression	Psychosis
SNOMED-CT	191618007	Bipolar affective disorder, current episode manic	Psychosis
SNOMED-CT	191625000	Bipolar affective disorder, currently manic, in full remission	Psychosis
SNOMED-CT	191634005	Bipolar affective disorder, currently depressed, in full remission	Psychosis
SNOMED-CT	13746004	Bipolar disorder	Psychosis
SNOMED-CT	63204009	Bouffee delirante	Psychosis
SNOMED-CT	191570001	Chronic schizoaffective schizophrenia	Psychosis
SNOMED-CT	2776000	Delirium - acute organic	Psychosis
SNOMED-CT	191507002	Delirium - subacute organic	Psychosis
SNOMED-CT	191483003	Drug psychosis	Psychosis
SNOMED-CT	760721000000109	Mixed bipolar affective disorder, partial/unspec remission	Psychosis
SNOMED-CT	764591000000108	Mixed bipolar affective disorder, severe	Psychosis
SNOMED-CT	69482004	Korsakov alcoholic psychosis	Psychosis
SNOMED-CT	231496004	Hypomanic psychoses	Psychosis
SNOMED-CT	35252006	Hebephrenic schizophrenia	Psychosis
SNOMED-CT	268612007	Dementia	Psychosis
SNOMED-CT	268612007	Other senile/presenile dement.	Psychosis
SNOMED-CT	268612007	Senile and presenile dementias	Psychosis
SNOMED-CT	191461002	Senile dementia-acute confused	Psychosis
SNOMED-CT	191526005	Schizophrenic psychoses NOS	Psychosis
SNOMED-CT	191526005	Schizophrenic psychoses	Psychosis
SNOMED-CT	191567000	Acute schizo affective psychosis	Psychosis
SNOMED-CT	13746004	Manic-depressive psychoses	Psychosis
SNOMED-CT	268619003	Mania/hypomania	Psychosis
SNOMED-CT	191667009	Paranoia	Psychosis
SNOMED-CT	386810004	Phobic state	Psychosis
SNOMED-CT	46975003	[X]Mental behav disorders due use crack cocaine: harmful use	Psychosis
SNOMED-CT	46975003	[X]Mental and behavioural disorders due to use of crack cocaine: psychotic disorder	Psychosis
SNOMED-CT	216004	Paranoid reaction	Psychosis
SNOMED-CT	216004	Persecutory delusion	Psychosis
SNOMED-CT	2073000	Delusional thoughts	Psychosis
SNOMED-CT	2073000	Delusional ideas	Psychosis
SNOMED-CT	2776000	Organic brain syndrome	Psychosis
SNOMED-CT	2776000	Acute psycho-organic syndrome	Psychosis

SNOMED-CT	2776000	Acute brain syndrome	Psychosis
SNOMED-CT	5464005	Brief psychotic disorder	Psychosis
SNOMED-CT	5510009	Paranoid organic state	Psychosis
SNOMED-CT	7052005	Alcohol hallucinosis	Psychosis
SNOMED-CT	18260003	Postnatal psychosis	Psychosis
SNOMED-CT	23645006	Organic affective disorder	Psychosis
SNOMED-CT	35252006	Disorganised schizophrenia	Psychosis
SNOMED-CT	61831009	Induced psychotic disorder	Psychosis
SNOMED-CT	61831009	Induced psychosis	Psychosis
SNOMED-CT	61831009	Induced paranoid disorder	Psychosis
SNOMED-CT	63204009	Bouffée délirante	Psychosis
SNOMED-CT	64269007	Seeing things	Psychosis
SNOMED-CT	69482004	Wernicke-Korsakoff syndrome	Psychosis
SNOMED-CT	69482004	Korsakoff psychosis	Psychosis
SNOMED-CT	69482004	Korsakov syndrome - alcoholic	Psychosis
SNOMED-CT	69482004	Korsakov psychosis	Psychosis
SNOMED-CT	69482004	Amnesic syndrome due to alcohol	Psychosis
SNOMED-CT	69482004	Alcoholic amnestic syndrome	Psychosis
SNOMED-CT	71961003	Childhood disintegrative disorder	Psychosis
SNOMED-CT	83746006	Chronic schizophrenia	Psychosis
SNOMED-CT	85248005	Bipolar disorder, in remission	Psychosis
SNOMED-CT	91388009	Disorder due to psychoactive substance use	Psychosis
SNOMED-CT	91388009	Psychoactive substance misuse disorder	Psychosis
SNOMED-CT	191461002	Senile delirium	Psychosis
SNOMED-CT	191483003	Drug-induced psychosis	Psychosis
SNOMED-CT	191483003	Drug induced psychosis	Psychosis
SNOMED-CT	191492000	Drug induced delirium	Psychosis
SNOMED-CT	191501001	Acute confusional state, post-traumatic	Psychosis
SNOMED-CT	191507002	Subacute delirium	Psychosis
SNOMED-CT	191508007	Subacute confusional state, post-traumatic	Psychosis
SNOMED-CT	191539009	Acute exacerbation of chronic disorganised schizophrenia	Psychosis
SNOMED-CT	191571002	Acute exacerbation of subchronic schizoaffective schizophrenia	Psychosis
SNOMED-CT	191572009	Acute exacerbation of chronic schizoaffective schizophrenia	Psychosis
SNOMED-CT	191667009	Paranoia	Psychosis
SNOMED-CT	191687005	Psychosis with origin in childhood	Psychosis
SNOMED-CT	192362008	Bipolar affective disorder, current episode mixed	Psychosis
SNOMED-CT	231437006	Reactive psychosis	Psychosis
SNOMED-CT	231485007	Post-schizophrenic depression	Psychosis
SNOMED-CT	274953007	Acute polymorphic psychotic disorder	Psychosis
SNOMED-CT	712850003	Acute polymorphic psychotic disorder with symptoms of schizophrenia	Psychosis

SNOMED-CT	760721000000109	Mixed bipolar affective disorder, in partial remission	Psychosis
SNOMED-CT	74934004	Psychoactive substance-induced withdrawal syndrome	Psychosis
Read	8G99.00	Sleep restriction therapy	Sleep disorders
Read	R005.11	[D]Insomnia - symptom	Sleep disorders
Read	Fy01.00	Disorders of excessive somnolence	Sleep disorders
Read	38D0.00	Pittsburgh sleep quality index	Sleep disorders
Read	1B6C.00	Excessive somnolence	Sleep disorders
Read	9Ngt.00	On melatonin for sleep disorder	Sleep disorders
Read	E274311	Hypersomnia NOS	Sleep disorders
Read	Fy06.00	Kleine-Levin syndrome	Sleep disorders
Read	E274700	Somnambulism - sleep walking	Sleep disorders
Read	8Q0..00	Sleep management	Sleep disorders
Read	R005000	[D]Sleep disturbance, unspecified	Sleep disorders
Read	K5A2100	Menopausal sleeplessness	Sleep disorders
Read	R005z00	[D]Sleep dysfunction NOS	Sleep disorders
Read	E274100	Transient insomnia	Sleep disorders
Read	E274200	Persistent insomnia	Sleep disorders
Read	E274000	Unspecified non-organic sleep disorder	Sleep disorders
Read	E274A00	Sleep drunkenness	Sleep disorders
Read	Eu51511	[X]Dream anxiety disorder	Sleep disorders
Read	E274D11	Restless sleep	Sleep disorders
Read	Eu51z11	[X]Emotional sleep disorder NOS	Sleep disorders
Read	1B1B.00	Cannot sleep - insomnia	Sleep disorders
Read	Z1M..00	Sleep and rest interventions	Sleep disorders
Read	Eu51z00	[X]Nonorganic sleep disorder, unspecified	Sleep disorders
Read	Fy0..00	Sleep disorders	Sleep disorders
Read	Eu51200	[X]Nonorganic disorder of the sleep-wake schedule	Sleep disorders
Read	Eu51.00	[X]Nonorganic sleep disorders	Sleep disorders
Read	1BX1.00	Excessive sleep	Sleep disorders
Read	E274400	Persistent hypersomnia	Sleep disorders
Read	E274.12	Insomnia due to nonorganic sleep disorder	Sleep disorders
Read	R005400	[D]Hypersomnia NOS	Sleep disorders
Read	E274z00	Non-organic sleep disorder NOS	Sleep disorders
Read	SL7z.11	Sleeping drug poisoning	Sleep disorders
Read	Eu51000	[X]Nonorganic insomnia	Sleep disorders
Read	R005.12	[D]Sleep rhythm problems	Sleep disorders
Read	1B1B000	Initial insomnia	Sleep disorders
Read	E274300	Transient hypersomnia	Sleep disorders
Read	TJ7z000	Adverse reaction to sleeping pill NOS	Sleep disorders
Read	E274B00	Repeated rapid eye movement sleep interruptions	Sleep disorders
Read	E274111	Insomnia NOS	Sleep disorders

Read	R005900	[D]Sleep dysfunction with arousal disturbance	Sleep disorders
Read	Eu51100	[X]Nonorganic hypersomnia	Sleep disorders
Read	1BX0.00	Delayed onset of sleep	Sleep disorders
Read	E274y00	Other non-organic sleep disorder	Sleep disorders
Read	1B1B.11	C/O - insomnia	Sleep disorders
Read	R000100	[D]Somnolence	Sleep disorders
Read	1B1B200	Late insomnia	Sleep disorders
Read	U1A2.11	[X]Accidental poisoning with sleeping tablets	Sleep disorders
Read	U607z12	[X] Adverse reaction to sleeping pill NOS	Sleep disorders
Read	E274D00	Repetitive intrusions of sleep	Sleep disorders
Read	Fyu5800	[X]Other sleep disorders	Sleep disorders
Read	R005800	[D]Sleep dysfunction with sleep stage disturbance	Sleep disorders
Read	E274C00	Other sleep stage or arousal dysfunction	Sleep disorders
Read	1B1B100	Middle insomnia	Sleep disorders
Read	E274.11	Hypersomnia of non-organic origin	Sleep disorders
Read	Fy00.00	Disorders of initiating and maintaining sleep	Sleep disorders
Read	Eu51y00	[X]Other nonorganic sleep disorders	Sleep disorders
Read	Eu51300	[X]Sleepwalking	Sleep disorders
Read	R005200	[D]Insomnia NOS	Sleep disorders
Read	E274.00	Non-organic sleep disorders	Sleep disorders
Read	R005.00	[D]Sleep disturbances	Sleep disorders
Read	1B1B.12	C/O - somnolence	Sleep disorders
Read	Fy02.00	Disorders of the sleep-wake schedule	Sleep disorders
Read	9Nk0.00	Seen in sleep clinic	Sleep disorders
Read	8HTn.00	Referral to sleep clinic	Sleep disorders
Read	38D1.00	Insomnia severity index	Sleep disorders
SNOMED-CT	12262002	Restless sleep	Sleep disorders
SNOMED-CT	59050008	Initial insomnia	Sleep disorders
SNOMED-CT	67233009	Middle insomnia	Sleep disorders
SNOMED-CT	111488004	Kleine-Levin syndrome	Sleep disorders
SNOMED-CT	162204000	Late insomnia	Sleep disorders
SNOMED-CT	270487001	Unspecified non-organic sleep disorder	Sleep disorders
SNOMED-CT	191997003	Persistent insomnia	Sleep disorders
SNOMED-CT	191999000	Persistent hypersomnia	Sleep disorders
SNOMED-CT	192003008	Sleep drunkenness	Sleep disorders
SNOMED-CT	192004002	Repeated rapid eye movement sleep interruptions	Sleep disorders
SNOMED-CT	39898005	Other sleep stage or arousal dysfunction	Sleep disorders
SNOMED-CT	270487001	Non-organic sleep disorder NOS	Sleep disorders
SNOMED-CT	270487001	[X]Other nonorganic sleep disorders	Sleep disorders
SNOMED-CT	270487001	[X]Nonorganic sleep disorder, unspecified	Sleep disorders

SNOMED-CT	194437008	Disorders of initiating and maintaining sleep	Sleep disorders
SNOMED-CT	194439006	Disorders of excessive somnolence	Sleep disorders
SNOMED-CT	39898005	[X]Other sleep disorders	Sleep disorders
SNOMED-CT	198437004	Menopausal sleeplessness	Sleep disorders
SNOMED-CT	271782001	Drowsiness	Sleep disorders
SNOMED-CT	271782001	Somnolence	Sleep disorders
SNOMED-CT	44186003	Sleep disturbance	Sleep disorders
SNOMED-CT	44186003	[D]Sleep disturbance, unspecified	Sleep disorders
SNOMED-CT	193462001	[D]Insomnia NOS	Sleep disorders
SNOMED-CT	77692006	Hypersomnia	Sleep disorders
SNOMED-CT	441877007	Sleep dysfunction with sleep stage disturbance	Sleep disorders
SNOMED-CT	442176004	Sleep dysfunction with arousal disturbance	Sleep disorders
SNOMED-CT	39898005	[D]Sleep dysfunction NOS	Sleep disorders
SNOMED-CT	85337000	Other sedative and hypnotic poisoning	Sleep disorders
SNOMED-CT	292331008	Adverse reaction to mixed sedatives NEC	Sleep disorders
SNOMED-CT	292331008	Adverse reaction to other sedatives and hypnotics	Sleep disorders
SNOMED-CT	292331008	Adverse reaction to other sedatives and hypnotics NOS	Sleep disorders
SNOMED-CT	292331008	Sedative adverse reaction	Sleep disorders
SNOMED-CT	292331008	Adverse reaction to sleeping pill NOS	Sleep disorders
SNOMED-CT	292331008	Adverse reaction to sedative NOS	Sleep disorders
SNOMED-CT	292331008	Adverse reaction to sedative or hypnotic NOS	Sleep disorders
SNOMED-CT	292331008	Adverse reaction to other tranquillisers	Sleep disorders
SNOMED-CT	292331008	Adverse reaction to tranquillisers NOS	Sleep disorders
SNOMED-CT	361149008	[X]Accidental poisoning with sleeping tablets	Sleep disorders
SNOMED-CT	193462001	Insomnia NOS	Sleep disorders
SNOMED-CT	77692006	Hypersomnia NOS	Sleep disorders
SNOMED-CT	230488004	Hypersomnia of non-organic origin	Sleep disorders
SNOMED-CT	268652009	Transient insomnia	Sleep disorders
SNOMED-CT	268653004	Transient hypersomnia	Sleep disorders
SNOMED-CT	268654005	Repetitive intrusions of sleep	Sleep disorders
SNOMED-CT	270487001	Other non-organic sleep disorder	Sleep disorders
SNOMED-CT	85337000	Poisoning by sedative AND/OR hypnotic	Sleep disorders
SNOMED-CT	85337000	Sedative and hypnotic drug poisoning NOS	Sleep disorders
SNOMED-CT	271794005	Disorders of the sleep-wake schedule	Sleep disorders
SNOMED-CT	272025006	Complaining of insomnia	Sleep disorders
SNOMED-CT	272026007	C/O - somnolence	Sleep disorders
SNOMED-CT	271794005	Disorder of sleep-wake cycle	Sleep disorders
SNOMED-CT	372947007	Excessive somnolence	Sleep disorders
SNOMED-CT	3745000	[D]Sleep rhythm problems	Sleep disorders

SNOMED-CT	193462001	[D]Insomnia - symptom	Sleep disorders
SNOMED-CT	77692006	Excessive sleep	Sleep disorders
SNOMED-CT	395069004	Sleep management	Sleep disorders
SNOMED-CT	401161007	Delayed onset of sleep	Sleep disorders
SNOMED-CT	428889007	Seen in sleep clinic	Sleep disorders
SNOMED-CT	39898005	Sleep disorder	Sleep disorders
SNOMED-CT	85337000	Sleeping drug poisoning	Sleep disorders
SNOMED-CT	80495009	Sleepwalking	Sleep disorders
SNOMED-CT	85337000	Sedative poisoning	Sleep disorders
SNOMED-CT	193462001	Insomnia	Sleep disorders
SNOMED-CT	270487001	Non-organic sleep disorder	Sleep disorders
SNOMED-CT	292331008	[X] Adverse reaction to mixed sedatives NEC	Sleep disorders
SNOMED-CT	292331008	[X] Adverse reaction to other major tranquilliser	Sleep disorders
SNOMED-CT	292331008	[X] Adverse reaction to other sedatives and hypnotics	Sleep disorders
SNOMED-CT	292331008	[X] Adverse reaction to other sedatives and hypnotics NOS	Sleep disorders
SNOMED-CT	292331008	[X] Adverse reaction to other tranquillisers	Sleep disorders
SNOMED-CT	292331008	[X] Adverse reaction to sedative NOS	Sleep disorders
SNOMED-CT	292331008	[X] Adverse reaction to sedative or hypnotic NOS	Sleep disorders
SNOMED-CT	292331008	[X] Adverse reaction to sleeping pill NOS	Sleep disorders
SNOMED-CT	292331008	[X] Adverse reaction to tranquillisers NOS	Sleep disorders
SNOMED-CT	361149008	Hypnotic intoxication	Sleep disorders
SNOMED-CT	419145002	[X]Dream anxiety disorder	Sleep disorders
SNOMED-CT	270487001	[X]Emotional sleep disorder NOS	Sleep disorders
SNOMED-CT	85337000	[X]Intentional self poisoning by and exposure to sedative hypnotics, occurrence at industrial and construction area	Sleep disorders
SNOMED-CT	85337000	[X]Intentional self poisoning by and exposure to sedative hypnotics, occurrence at sports and athletics area	Sleep disorders
SNOMED-CT	85337000	[X]Intentional self poisoning by and exposure to sedative hypnotics, occurrence at other specified place	Sleep disorders
SNOMED-CT	85337000	[X]Intentional self poisoning by and exposure to sedative hypnotics, occurrence at school, other institution and public administrative area	Sleep disorders
SNOMED-CT	85337000	[X]Intentional self poisoning by and exposure to sedative hypnotics, occurrence at home	Sleep disorders
SNOMED-CT	85337000	[X]Intentional self poisoning by and exposure to sedative hypnotics, occurrence on farm	Sleep disorders
SNOMED-CT	85337000	[X]Intentional self poisoning by and exposure to sedative hypnotics, occurrence on street and highway	Sleep disorders

SNOMED-CT	85337000	[X]Intentional self poisoning by and exposure to sedative hypnotics, occurrence at trade and service area	Sleep disorders
SNOMED-CT	85337000	[X]Intentional self poisoning by and exposure to sedative hypnotics, occurrence in residential institution	Sleep disorders
SNOMED-CT	85337000	[X]Intentional self poisoning by and exposure to sedative hypnotics, occurrence at unspecified place	Sleep disorders
SNOMED-CT	419145002	[X]Nightmares	Sleep disorders
SNOMED-CT	268722008	[X]Nonorganic disorder of the sleep-wake schedule	Sleep disorders
SNOMED-CT	230488004	[X]Nonorganic hypersomnia	Sleep disorders
SNOMED-CT	192454004	Nonorganic insomnia	Sleep disorders
SNOMED-CT	270487001	[X]Nonorganic sleep disorders	Sleep disorders
SNOMED-CT	85337000	[X]Poisoning by and exposure to sedative hypnotics, occurrence at sports and athletics area, undetermined intent	Sleep disorders
SNOMED-CT	85337000	[X]Poisoning by and exposure to sedative hypnotics, occurrence at industrial and construction area, undetermined intent	Sleep disorders
SNOMED-CT	85337000	[X]Poisoning by and exposure to sedative hypnotics, occurrence at school, other institution and public administrative area, undetermined intent	Sleep disorders
SNOMED-CT	85337000	[X]Poisoning by and exposure to sedative hypnotics, occurrence at other specified place, undetermined intent	Sleep disorders
SNOMED-CT	85337000	[X]Poisoning by and exposure to sedative hypnotics, occurrence on street and highway, undetermined intent	Sleep disorders
SNOMED-CT	85337000	[X]Poisoning by and exposure to sedative hypnotics, occurrence at trade and service area, undetermined intent	Sleep disorders
SNOMED-CT	85337000	[X]Poisoning by and exposure to sedative hypnotics, occurrence in residential institution, undetermined intent	Sleep disorders
SNOMED-CT	85337000	[X]Poisoning by and exposure to sedative hypnotics, occurrence at unspecified place, undetermined intent	Sleep disorders
SNOMED-CT	85337000	[X]Poisoning by and exposure to sedative hypnotics, occurrence at home, undetermined intent	Sleep disorders
SNOMED-CT	85337000	[X]Poisoning by and exposure to sedative hypnotics, occurrence on farm, undetermined intent	Sleep disorders
SNOMED-CT	85337000	[X]Poisoning by and exposure to sedative hypnotics, undetermined intent	Sleep disorders
SNOMED-CT	268722008	[X]Psychogenic inversion of circadian rhythm	Sleep disorders
SNOMED-CT	268722008	[X]Psychogenic inversion of nyctohemeral rhythm	Sleep disorders
SNOMED-CT	268722008	[X]Psychogenic inversion of sleep rhythm	Sleep disorders
SNOMED-CT	80495009	[X]Sleepwalking	Sleep disorders

SNOMED-CT	292331008	Adverse reaction to other major tranquillisers	Sleep disorders
SNOMED-CT	271782001	Drowsiness - symptom	Sleep disorders
SNOMED-CT	438517001	Referral to sleep clinic	Sleep disorders
SNOMED-CT	440089000	Sleep restriction therapy	Sleep disorders
SNOMED-CT	419145002	Nightmares	Sleep disorders
SNOMED-CT	192454004	Insomnia due to nonorganic sleep disorder	Sleep disorders
SNOMED-CT	85337000	Hypnotic poisoning	Sleep disorders
SNOMED-CT	77692006	Hypersomnia	Sleep disorders
SNOMED-CT	111488004	Kleine-Levin syndrome	Sleep disorders
SNOMED-CT	887881000000101	On melatonin for sleep disorder	Sleep disorders
SNOMED-CT	3745000	Sleep-wake schedule disorder	Sleep disorders
SNOMED-CT	3745000	Circadian rhythm sleep disorder	Sleep disorders
SNOMED-CT	3745000	Sleep rhythm problem	Sleep disorders
SNOMED-CT	44186003	Dyssomnia	Sleep disorders
SNOMED-CT	44186003	Sleep problem	Sleep disorders
SNOMED-CT	59050008	Difficulty falling asleep	Sleep disorders
SNOMED-CT	59050008	Difficulty in sleep initiation	Sleep disorders
SNOMED-CT	59050008	Difficulty getting to sleep	Sleep disorders
SNOMED-CT	67233009	Night waking	Sleep disorders
SNOMED-CT	67233009	Broken sleep	Sleep disorders
SNOMED-CT	67233009	Interrupted sleep	Sleep disorders
SNOMED-CT	67233009	Difficulty in sleep maintenance	Sleep disorders
SNOMED-CT	77692006	Sleeps too much	Sleep disorders
SNOMED-CT	77692006	Excessive sleepiness	Sleep disorders
SNOMED-CT	80495009	Sleep walking disorder	Sleep disorders
SNOMED-CT	80495009	Somnambulism	Sleep disorders
SNOMED-CT	80495009	Sleep walking	Sleep disorders
SNOMED-CT	194439006	Disorder of excessive somnolence	Sleep disorders
SNOMED-CT	268722008	Non-organic disorder of the sleep-wake schedule	Sleep disorders
SNOMED-CT	271782001	Drowsy	Sleep disorders
SNOMED-CT	271782001	Sleepy	Sleep disorders
SNOMED-CT	271794005	Circadian dysregulation	Sleep disorders
SNOMED-CT	272026007	Complaining of somnolence	Sleep disorders

Supplementary Table 3. Description of the number of eligible patients, number of Parkinson's disease cases, and mean follow-up in each emulated trial, before and after matching.

		Before matching			After matching		
Trial	Treatment	No. of patients	No. of Parkinson's diagnosis	Mean follow-up	No. of patients	No. of Parkinson's diagnosis	Mean follow-up
1	No LTRA	378702	513	8.16	924	2	7.76
1	LTRA	924	1	7.79	924	1	7.79
2	No LTRA	379768	517	8.02	957	4	7.26
2	LTRA	957	2	7.23	957	2	7.23
3	No LTRA	381378	524	7.87	1114	1	6.96
3	LTRA	1114	1	7.16	1114	1	7.16
4	No LTRA	369806	576	8.29	1032	2	7.47
4	LTRA	1032	1	7.30	1032	1	7.30
5	No LTRA	371600	573	8.13	930	0	7.33
5	LTRA	930	2	7.38	930	2	7.38
6	No LTRA	372653	582	7.99	902	1	7.50
6	LTRA	902	2	7.60	902	2	7.60
7	No LTRA	374443	572	7.83	1030	0	7.19
7	LTRA	1030	0	6.90	1030	0	6.90
8	No LTRA	363509	640	8.27	1087	2	7.80
8	LTRA	1088	2	7.80	1087	2	7.80
9	No LTRA	365226	643	8.11	880	5	7.48
9	LTRA	880	2	7.74	880	2	7.74
10	No LTRA	366394	644	7.97	904	0	7.48
10	LTRA	904	2	7.54	904	2	7.54
11	No LTRA	367717	644	7.82	1081	0	7.19
11	LTRA	1081	0	7.10	1081	0	7.10
12	No LTRA	357297	731	8.28	1122	4	7.77
12	LTRA	1122	2	7.68	1122	2	7.68
13	No LTRA	359257	737	8.12	867	1	7.55
13	LTRA	867	3	7.73	867	3	7.73
14	No LTRA	360232	747	7.99	922	1	7.33
14	LTRA	922	0	7.33	922	0	7.33
15	No LTRA	361911	739	7.83	1023	5	6.88
15	LTRA	1023	4	7.09	1023	4	7.09
16	No LTRA	351274	814	8.28	1116	1	7.67
16	LTRA	1116	4	7.98	1116	4	7.98
17	No LTRA	353142	821	8.13	878	6	7.70
17	LTRA	878	0	7.84	878	0	7.84
18	No LTRA	354240	828	7.99	956	0	7.66
18	LTRA	956	1	7.79	956	1	7.79
19	No LTRA	355612	837	7.84	1110	2	6.93
19	LTRA	1111	1	7.27	1110	1	7.28

20	No LTRA	343139	906	8.26	1044	3	7.54
20	LTRA	1044	5	7.48	1044	5	7.48
21	No LTRA	345143	916	8.11	898	2	7.68
21	LTRA	898	2	7.49	898	2	7.49
22	No LTRA	346205	922	7.97	947	2	7.30
22	LTRA	947	1	7.33	947	1	7.33
23	No LTRA	347543	926	7.82	1047	1	6.92
23	LTRA	1047	3	7.22	1047	3	7.22
24	No LTRA	335418	1014	8.28	1037	1	7.71
24	LTRA	1037	4	7.85	1037	4	7.85
25	No LTRA	337127	1018	8.13	920	1	7.74
25	LTRA	920	0	7.57	920	0	7.57
26	No LTRA	337925	1015	7.99	942	5	7.71
26	LTRA	942	2	7.64	942	2	7.64
27	No LTRA	339248	1011	7.84	1003	3	7.03
27	LTRA	1004	3	7.38	1003	3	7.38
28	No LTRA	327074	1102	8.30	1012	3	8.08
28	LTRA	1012	4	8.02	1012	4	8.02
29	No LTRA	328823	1105	8.15	832	3	7.48
29	LTRA	832	6	7.64	832	6	7.64
30	No LTRA	329738	1105	8.01	887	0	7.47
30	LTRA	887	2	7.46	887	2	7.46
31	No LTRA	331192	1112	7.86	1013	1	6.98
31	LTRA	1013	3	7.35	1013	3	7.35
32	No LTRA	319593	1207	8.30	1006	3	7.79
32	LTRA	1006	5	8.08	1006	5	8.08
33	No LTRA	321212	1219	8.15	910	6	7.37
33	LTRA	910	6	7.29	910	6	7.29
34	No LTRA	322120	1219	8.01	871	3	7.48
34	LTRA	871	5	7.66	871	5	7.66
35	No LTRA	323496	1213	7.86	1000	7	7.04
35	LTRA	1000	7	7.07	1000	7	7.07
36	No LTRA	312171	1311	8.29	979	6	7.62
36	LTRA	979	1	7.66	979	1	7.66
37	No LTRA	313808	1323	8.13	840	3	7.57
37	LTRA	840	1	7.64	840	1	7.64
38	No LTRA	314673	1329	7.99	837	1	6.98
38	LTRA	837	6	7.28	837	6	7.28
39	No LTRA	316025	1334	7.85	918	5	7.21
39	LTRA	918	4	7.19	918	4	7.19
40	No LTRA	305005	1434	8.26	1027	4	7.64
40	LTRA	1027	8	7.65	1027	8	7.65
41	No LTRA	306590	1432	8.12	863	3	7.54
41	LTRA	863	1	7.63	863	1	7.63

42	No LTRA	307550	1437	7.98	833	4	7.62
42	LTRA	833	3	7.70	833	3	7.70
43	No LTRA	308851	1440	7.84	934	4	6.78
43	LTRA	934	4	7.24	934	4	7.24
44	No LTRA	299011	1542	8.27	953	5	7.29
44	LTRA	953	3	7.73	953	3	7.73
45	No LTRA	300562	1549	8.12	905	2	7.52
45	LTRA	906	4	7.54	905	4	7.55
46	No LTRA	301443	1559	7.99	846	7	7.31
46	LTRA	846	5	7.68	846	5	7.68
47	No LTRA	302566	1555	7.85	976	11	6.95
47	LTRA	976	6	7.28	976	6	7.28
48	No LTRA	294471	1692	8.25	969	4	7.36
48	LTRA	969	0	7.88	969	0	7.88
49	No LTRA	296103	1703	8.10	829	5	7.65
49	LTRA	829	5	7.69	829	5	7.69
50	No LTRA	297161	1705	7.96	818	6	7.64
50	LTRA	818	5	7.64	818	5	7.64
51	No LTRA	298575	1708	7.82	893	3	6.82
51	LTRA	893	6	6.97	893	6	6.97
52	No LTRA	290839	1831	8.21	956	7	7.89
52	LTRA	956	4	8.10	956	4	8.10
53	No LTRA	292046	1832	8.06	855	5	7.84
53	LTRA	855	9	7.86	855	9	7.86
54	No LTRA	292856	1819	7.93	768	1	7.48
54	LTRA	768	5	7.36	768	5	7.36
55	No LTRA	294145	1827	7.78	811	7	7.28
55	LTRA	811	1	7.14	811	1	7.14
56	No LTRA	286972	1946	8.16	931	2	7.25
56	LTRA	931	8	7.70	931	8	7.70
57	No LTRA	288047	1946	8.02	793	7	8.01
57	LTRA	793	4	7.87	793	4	7.87
58	No LTRA	288800	1941	7.89	776	5	7.30
58	LTRA	776	5	7.24	776	5	7.24
59	No LTRA	289976	1947	7.74	818	5	6.86
59	LTRA	818	3	7.01	818	3	7.01
60	No LTRA	283174	2038	8.10	988	6	7.46
60	LTRA	988	10	7.35	988	10	7.35
61	No LTRA	284225	2052	7.96	778	7	7.29
61	LTRA	778	5	7.26	778	5	7.26
62	No LTRA	284908	2046	7.82	700	6	7.20
62	LTRA	700	7	7.26	700	7	7.26
63	No LTRA	285691	2050	7.68	844	3	7.13
63	LTRA	844	3	7.21	844	3	7.21

64	No LTRA	279749	2187	8.01	902	14	7.48
64	LTRA	902	2	7.55	902	2	7.55
65	No LTRA	280752	2189	7.87	740	10	7.33
65	LTRA	740	6	7.81	740	6	7.81
66	No LTRA	281427	2189	7.73	691	4	7.42
66	LTRA	691	2	7.20	691	2	7.20
67	No LTRA	282201	2189	7.59	828	6	7.15
67	LTRA	828	5	7.16	828	5	7.16
68	No LTRA	275876	2301	7.90	879	8	7.10
68	LTRA	879	8	7.42	879	8	7.42
69	No LTRA	276932	2299	7.75	760	4	7.10
69	LTRA	760	11	7.27	760	11	7.27
70	No LTRA	277503	2287	7.61	687	8	7.50
70	LTRA	687	2	7.16	687	2	7.16
71	No LTRA	278002	2290	7.47	797	6	7.19
71	LTRA	797	4	6.97	797	4	6.97
72	No LTRA	271516	2394	7.77	865	9	7.46
72	LTRA	865	10	7.52	865	10	7.52
73	No LTRA	272361	2382	7.63	688	4	7.01
73	LTRA	688	9	7.31	688	9	7.31
74	No LTRA	272648	2363	7.49	657	4	7.01
74	LTRA	657	3	7.43	657	3	7.43
75	No LTRA	273235	2348	7.34	775	3	6.49
75	LTRA	775	6	7.00	775	6	7.00
76	No LTRA	267265	2443	7.63	856	7	7.06
76	LTRA	856	10	7.06	856	10	7.06
77	No LTRA	268063	2419	7.48	717	5	7.35
77	LTRA	717	8	7.03	717	8	7.03
78	No LTRA	268243	2399	7.34	689	3	6.77
78	LTRA	689	5	7.01	689	5	7.01
79	No LTRA	268587	2370	7.20	741	4	6.48
79	LTRA	741	3	6.60	741	3	6.60
80	No LTRA	263398	2467	7.47	778	14	7.07
80	LTRA	778	9	7.30	778	9	7.30
81	No LTRA	263748	2436	7.33	645	3	6.84
81	LTRA	645	11	6.96	645	11	6.96
82	No LTRA	263814	2405	7.19	634	10	6.40
82	LTRA	634	3	6.47	634	3	6.47
83	No LTRA	264013	2384	7.05	693	2	6.42
83	LTRA	693	3	6.65	693	3	6.65
84	No LTRA	257959	2460	7.31	751	4	6.94
84	LTRA	751	11	6.82	751	11	6.82
85	No LTRA	258202	2404	7.17	646	0	7.18
85	LTRA	646	7	7.16	646	7	7.16

86	No LTRA	258080	2388	7.03	606	7	6.64
86	LTRA	606	12	6.52	606	12	6.52
87	No LTRA	257842	2373	6.90	645	3	6.34
87	LTRA	645	2	6.26	645	2	6.26
88	No LTRA	251186	2448	7.15	745	9	6.89
88	LTRA	745	13	7.00	745	13	7.00
89	No LTRA	251427	2424	7.00	624	3	6.54
89	LTRA	624	7	6.74	624	7	6.74
90	No LTRA	251214	2393	6.87	531	4	6.71
90	LTRA	531	8	6.63	531	8	6.63
91	No LTRA	251032	2376	6.73	633	5	6.23
91	LTRA	633	6	6.30	633	6	6.30
92	No LTRA	243435	2445	6.98	638	10	7.49
92	LTRA	638	6	7.21	638	6	7.21
93	No LTRA	243492	2420	6.84	623	11	6.29
93	LTRA	623	6	6.35	623	6	6.35
94	No LTRA	242919	2389	6.70	532	5	6.45
94	LTRA	532	6	6.55	532	6	6.55
95	No LTRA	242467	2360	6.57	620	6	6.08
95	LTRA	620	3	6.11	620	3	6.11
96	No LTRA	233079	2389	6.85	642	6	6.25
96	LTRA	642	7	6.60	642	7	6.60
97	No LTRA	232861	2346	6.71	577	9	6.36
97	LTRA	577	6	6.63	577	6	6.63
98	No LTRA	232374	2293	6.57	514	5	6.43
98	LTRA	514	4	6.30	514	4	6.30
99	No LTRA	231713	2265	6.44	520	8	5.91
99	LTRA	520	4	6.19	520	4	6.19
100	No LTRA	223199	2292	6.68	596	6	5.91
100	LTRA	596	4	6.39	596	4	6.39
101	No LTRA	222649	2220	6.54	532	5	5.60
101	LTRA	532	11	6.13	532	11	6.13
102	No LTRA	221959	2187	6.41	485	4	5.97
102	LTRA	485	3	6.25	485	3	6.25
103	No LTRA	221065	2135	6.28	486	5	6.00
103	LTRA	486	2	6.11	486	2	6.11
104	No LTRA	214826	2147	6.47	538	2	6.30
104	LTRA	538	5	6.37	538	5	6.37
105	No LTRA	214302	2090	6.33	454	0	6.07
105	LTRA	454	8	6.47	454	8	6.47
106	No LTRA	213452	2044	6.20	460	4	6.01
106	LTRA	460	5	6.06	460	5	6.06
107	No LTRA	212520	2004	6.07	502	3	5.88
107	LTRA	502	7	6.02	502	7	6.02

108	No LTRA	205367	2019	6.27	507	4	6.12
108	LTRA	507	3	6.06	507	3	6.06
109	No LTRA	204753	1978	6.13	426	2	5.71
109	LTRA	426	4	6.22	426	4	6.22
110	No LTRA	203564	1923	6.00	355	2	5.76
110	LTRA	355	6	6.13	355	6	6.13
111	No LTRA	202371	1880	5.88	433	3	5.58
111	LTRA	433	5	5.36	433	5	5.36
112	No LTRA	196014	1879	6.04	441	3	5.63
112	LTRA	441	3	6.02	441	3	6.02
113	No LTRA	194907	1807	5.91	393	4	5.51
113	LTRA	393	4	5.36	393	4	5.36
114	No LTRA	193594	1722	5.78	362	3	5.61
114	LTRA	362	6	5.71	362	6	5.71
115	No LTRA	192090	1658	5.66	397	5	5.26
115	LTRA	397	1	5.25	397	1	5.25
116	No LTRA	186049	1666	5.81	433	4	5.59
116	LTRA	433	2	5.70	433	2	5.70
117	No LTRA	184785	1610	5.68	350	3	5.23
117	LTRA	350	5	5.74	350	5	5.74
118	No LTRA	183546	1557	5.55	271	2	5.24
118	LTRA	271	4	5.75	271	4	5.75
119	No LTRA	181845	1500	5.43	323	4	5.23
119	LTRA	323	5	5.27	323	5	5.27
120	No LTRA	177278	1512	5.55	375	4	5.39
120	LTRA	375	1	5.39	375	1	5.39
121	No LTRA	175748	1467	5.43	304	1	5.14
121	LTRA	304	2	5.02	304	2	5.02
122	No LTRA	174271	1420	5.31	283	3	5.26
122	LTRA	283	2	5.65	283	2	5.65
123	No LTRA	172401	1372	5.18	315	2	5.17
123	LTRA	315	2	5.39	315	2	5.39
124	No LTRA	166028	1350	5.28	319	3	5.39
124	LTRA	319	1	5.45	319	1	5.45
125	No LTRA	164320	1307	5.16	268	0	4.87
125	LTRA	268	4	4.96	268	4	4.96
126	No LTRA	162391	1252	5.04	236	2	4.72
126	LTRA	236	2	4.96	236	2	4.96
127	No LTRA	160306	1206	4.92	274	3	4.62
127	LTRA	274	2	5.08	274	2	5.08
128	No LTRA	152728	1170	5.03	299	0	4.95
128	LTRA	299	7	5.11	299	7	5.11
129	No LTRA	150836	1128	4.91	217	1	4.74
129	LTRA	217	1	4.86	217	1	4.86

130	No LTRA	148974	1085	4.79	220	2	4.83
130	LTRA	220	2	4.76	220	2	4.76
131	No LTRA	146726	1022	4.68	256	1	4.25
131	LTRA	256	2	4.93	256	2	4.93
132	No LTRA	139045	986	4.78	203	3	4.09
132	LTRA	203	1	4.84	203	1	4.84
133	No LTRA	136945	936	4.67	171	2	4.40
133	LTRA	171	2	4.80	171	2	4.80
134	No LTRA	134977	883	4.55	179	1	4.64
134	LTRA	179	0	4.63	179	0	4.63
135	No LTRA	132580	817	4.44	160	0	4.26
135	LTRA	160	1	4.45	160	1	4.45
136	No LTRA	125318	780	4.53	165	0	4.36
136	LTRA	165	1	4.67	165	1	4.67
137	No LTRA	123130	727	4.43	135	0	4.66
137	LTRA	135	2	4.12	135	2	4.12
138	No LTRA	121026	690	4.31	118	0	4.42
138	LTRA	119	1	4.85	118	1	4.87
139	No LTRA	118540	644	4.21	148	2	4.21
139	LTRA	148	1	4.27	148	1	4.27
140	No LTRA	111349	614	4.29	149	1	4.38
140	LTRA	149	1	4.12	149	1	4.12
Total	No LTRA	37171790	222620	7.36	97049	537	6.99
Total	LTRA	97054	573	7.09	97049	573	7.09

LTRA, leukotriene receptor antagonist.

Supplementary Table 4. Full baseline characteristics of patients who initiated LTRA treatment and did not initiate LTRA treatment pooling all emulated trials, before and after matching (pooled emulated trials).

	**Before matching**			**After matching**		
	**LTRA**	**No LTRA**	**SMD**	**LTRA**	**No LTRA**	**SMD**
	**N=97,054**	**N=37,171,790**		**N=97,049**	**N=97,049**	
**Age, years [IQR]**	62.5 [56-70.3]	63.8 [56.3-72.5]	-0.124	62.5 [56-70.3]	62.5 [56-70.3]	0
**Female sex, n (%)**	61,853 (63.7)	21,327,141 (57.4)	-0.130	61,849 (63.7)	61,834 (63.7)	0
**Duration since asthma diagnosis, years [IQR]**	13.7 [6.2-23.8]	12.6 [6.2-21.2]	0.093	13.7 [6.2-23.8]	14.0 [7.0-23.2]	-0.002
**No. hospitalisation within in past 180 days, n (%)**			0.025			0.001
0	79,471 (81.9)	31,884,742 (85.8)		79,468 (81.9)	79,555 (82.0)	
1	12,657 (13.0)	3,732,872 (10.0)		12,656 (13.0)	12,540 (12.9)	
2 or more	4,926 (5.1)	1,554,176 (4.2)		4,925 (5.1)	4,954 (5.1)	
**No. asthma hospitalisation in past 180 days, n (%)**			0.195			0.014
0	86,979 (89.6)	35,327,369 (95.0)		86,976 (89.6)	87,311 (90.0)	
1	7,983 (8.2)	1,482,740 (4.0)		7,982 (8.2)	7,770 (8.0)	
2 or more	2,092 (2.2)	361,681 (1.0)		2,091 (2.2)	1,968 (2.0)	
**IMD quintiles, n (%)**						
1 (most deprived)	20,095 (20.7)	8,130,533 (21.9)	-0.029	20,095 (20.7)	20,017 (20.6)	0.002
2	20,533 (21.2)	7,870,390 (21.2)	0	20,532 (21.2)	20,559 (21.2)	-0.001
3	18,666 (19.2)	7,060,718 (19)	0.006	18,666 (19.2)	18,790 (19.4)	-0.003
4	17,949 (18.5)	6,748,996 (18.2)	0.009	17,948 (18.5)	17,937 (18.5)	0
5 (least deprived)	18,257 (18.8)	6,651,835 (17.9)	0.024	18,257 (18.8)	18,179 (18.7)	0.002
**Smoking status, n (%)**						
Current smoker	11,090 (11.4)	6,184,540 (16.6)	-0.15	11,090 (11.4)	11,279 (11.6)	-0.006
Ex smoker	33,971 (35)	12,689,159 (34.1)	0.018	33,971 (35)	34,148 (35.2)	-0.004
Non-smoker	49,672 (51.2)	16,888,465 (45.4)	0.115	49,668 (51.2)	49,384 (50.9)	0.006

Unknown	2,321 (2.4)	1,409,626 (3.8)	-0.081	2,320 (2.4)	2,238 (2.3)	0.006
**BMI class, n (%)**						
Underweight (<18.5 kg/m^2)	1,200 (1.2)	667,765 (1.8)	-0.046	1,200 (1.2)	1,222 (1.3)	-0.002
Normal weight (18.5-24.9 kg/m^2)	22,857 (23.6)	9,975,694 (26.8)	-0.076	22,857 (23.6)	22,940 (23.6)	-0.002
Overweight (25.0-29.9 kg/m^2)	33,290 (34.3)	12,946,335 (34.8)	-0.011	33,287 (34.3)	33,118 (34.1)	0.004
Obese (>=30.0 kg/m^2)	35,993 (37.1)	11,376,463 (30.6)	0.137	35,991 (37.1)	36,114 (37.2)	-0.003
Unknown	3,714 (3.8)	2,205,533 (5.9)	-0.098	3,714 (3.8)	3,655 (3.8)	0.003
**Eosinophilia in past 180 days, n (%)**	4,841 (5.0)	1,023,614 (2.8)	0.116	4,840 (5.0)	4,745 (4.9)	0.005
**Electronic frailty index, n (%)**						
Fit	54,785 (56.4)	23,200,130 (62.4)	-0.122	54,782 (56.4)	54,787 (56.5)	0
Mild frailty	32,955 (34)	10,832,016 (29.1)	0.104	32,954 (34)	32,821 (33.8)	0.003
Moderate frailty	7,964 (8.2)	2,613,037 (7)	0.044	7,964 (8.2)	8,068 (8.3)	-0.004
Severe frailty	1,350 (1.4)	526,607 (1.4)	-0.002	1,349 (1.4)	1,373 (1.4)	-0.002
**Comorbidity history, n (%)**						
Allergic rhinitis	36,697 (37.8)	9,795,616 (26.4)	0.247	36,695 (37.8)	36,505 (37.6)	0.004
Atopic dermatitis	25,065 (25.8)	8,306,651 (22.3)	0.081	25,064 (25.8)	25,053 (25.8)	0
Alcohol-related disorder	455 (0.5)	182,971 (0.5)	-0.003	455 (0.5)	453 (0.5)	0
Cancer	7,846 (8.1)	3,421,846 (9.2)	-0.04	7,846 (8.1)	7,929 (8.2)	-0.003
Chronic kidney disease	6,609 (6.8)	2,692,281 (7.2)	-0.017	6,607 (6.8)	6,710 (6.9)	-0.004
Chronic obstructive pulmonary disease	16,447 (16.9)	6,514,676 (17.5)	-0.015	16,447 (16.9)	16,792 (17.3)	-0.009
Dementia	403 (0.4)	352,404 (0.9)	-0.065	403 (0.4)	407 (0.4)	-0.001
Diabetes	11,409 (11.8)	4,771,726 (12.8)	-0.033	11,407 (11.8)	11,510 (11.9)	-0.003
Gout	5,158 (5.3)	2,055,737 (5.5)	-0.01	5,158 (5.3)	5,057 (5.2)	0.005
Liver disease	2,073 (2.1)	657,359 (1.8)	0.027	2,073 (2.1)	2,086 (2.1)	-0.001
Hypertension	37,370 (38.5)	14,380,000 (38.7)	-0.004	37,368 (38.5)	37,317 (38.5)	0.001
Myocardial infarction	3,073 (3.2)	1,578,945 (4.2)	-0.057	3,073 (3.2)	3,137 (3.2)	-0.004
Stroke	534 (0.6)	280,190 (0.8)	-0.025	534 (0.6)	552 (0.6)	-0.002

Depression	29,189 (30.1)	9,074,469 (24.4)	0.127	29,187 (30.1)	29,420 (30.3)	-0.005
Sleep disorder	14,097 (14.5)	4,318,473 (11.6)	0.086	14,094 (14.5)	14,054 (14.5)	0.001
Epilepsy	1,846 (1.9)	708,096 (1.9)	0	1,845 (1.9)	1,891 (1.9)	-0.003
Psychosis	1,808 (1.9)	762,315 (2.1)	-0.014	1,808 (1.9)	1,861 (1.9)	-0.004
Head injury	3,099 (3.2)	1,048,375 (2.8)	0.022	3,098 (3.2)	3,074 (3.2)	0.001
Fall	10,913 (11.2)	3,701,985 (10)	0.042	10,912 (11.2)	10,798 (11.1)	0.004
Fracture	25,366 (26.1)	9,264,920 (24.9)	0.028	25,363 (26.1)	25,568 (26.3)	-0.005
Lower respiratory tract infection	62,521 (64.4)	19,426,887 (52.3)	0.248	62,516 (64.4)	62,216 (64.1)	0.006
Influenza infection	12,244 (12.6)	3,819,697 (10.3)	0.074	12,244 (12.6)	12,084 (12.5)	0.005
**Concurrent medications, n (%)**						
Short-acting beta-agonist	75,090 (77.4)	18,153,217 (48.8)	0.619	75,085 (77.4)	75,494 (77.8)	-0.001
Long-acting beta-agonist	69,609 (71.7)	10,828,920 (29.1)	0.941	69,604 (71.7)	70,134 (72.3)	-0.012
Muscarinic antagonist	18,780 (19.4)	4,824,024 (13)	0.174	18,780 (19.4)	18,808 (19.4)	-0.001
Inhaled corticosteroid	84,287 (86.8)	19,156,597 (51.5)	0.828	84,282 (86.8)	84,977 (87.6)	-0.021
Oral corticosteroid	36,772 (37.9)	4,615,102 (12.4)	0.614	36,767 (37.9)	36,459 (37.6)	0.007
Xanthine-derived bronchodilator	4,549 (4.7)	800,864 (2.2)	0.014	4,548 (4.7)	4,485 (4.6)	0.003
Antihistamine	22,180 (22.9)	4,012,828 (10.8)	0.327	22,178 (22.9)	21,918 (22.6)	0.006
Aspirin	11,591 (11.9)	5,515,853 (14.8)	-0.085	11,590 (11.9)	11,820 (12.2)	-0.007
Calcium channel blocker	18,902 (19.5)	6,951,515 (18.7)	0.02	18,901 (19.5)	19,070 (19.6)	-0.004
Statin	27,614 (28.5)	10,708,664 (28.8)	-0.008	27,612 (28.5)	27,726 (28.6)	-0.003
Antianxiolytic and sedative	8,513 (8.8)	2,770,137 (7.5)	0.048	8,513 (8.8)	8,597 (8.9)	-0.003
Antidepressant	24,401 (25.1)	6,937,977 (18.7)	0.157	24,400 (25.1)	24,452 (25.2)	-0.001
Antipsychotic	3,891 (4)	1,273,089 (3.4)	0.031	3,891 (4)	3,793 (3.9)	0.005
Metformin	7,473 (7.7)	3,028,263 (8.1)	-0.017	7,473 (7.7)	7,498 (7.7)	-0.001
Incretin-based drug	1,663 (1.7)	576,481 (1.6)	0.013	1,663 (1.7)	1,717 (1.8)	-0.004
Insulin	2,575 (2.7)	963,327 (2.6)	0.004	2,574 (2.7)	2,514 (2.6)	0.004
**Calendar year, n (%)**						

2000	1,890 (1.9)	1,025,889 (2.8)	-0.054	1,889 (1.9)	1,862 (1.9)	0.002
2001	1,926 (2)	1,128,062 (3)	-0.067	1,926 (2)	1,877 (1.9)	0.004
2002	2,112 (2.2)	1,229,606 (3.3)	-0.069	2,112 (2.2)	2,178 (2.2)	-0.005
2003	2,575 (2.7)	1,326,435 (3.6)	-0.053	2,575 (2.7)	2,516 (2.6)	0.004
2004	2,903 (3)	1,432,118 (3.9)	-0.047	2,903 (3)	2,900 (3)	0
2005	3,875 (4)	1,527,493 (4.1)	-0.006	3,875 (4)	3,669 (3.8)	0.011
2006	3,990 (4.1)	1,593,023 (4.3)	-0.009	3,990 (4.1)	3,915 (4)	0.004
2007	3,962 (4.1)	1,652,385 (4.4)	-0.018	3,962 (4.1)	3,916 (4)	0.002
2008	4,166 (4.3)	1,712,232 (4.6)	-0.015	4,166 (4.3)	4,092 (4.2)	0.004
2009	4,302 (4.4)	1,775,151 (4.8)	-0.016	4,302 (4.4)	4,338 (4.5)	-0.002
2010	4,417 (4.6)	1,831,929 (4.9)	-0.018	4,417 (4.6)	4,380 (4.5)	0.002
2011	4,649 (4.8)	1,887,539 (5.1)	-0.013	4,649 (4.8)	4,769 (4.9)	-0.006
2012	4,755 (4.9)	1,948,301 (5.2)	-0.016	4,755 (4.9)	4,633 (4.8)	0.006
2013	4,973 (5.1)	2,007,005 (5.4)	-0.012	4,973 (5.1)	4,923 (5.1)	0.002
2014	5,306 (5.5)	2,055,901 (5.5)	-0.003	5,306 (5.5)	5,298 (5.5)	0
2015	5,407 (5.6)	2,092,774 (5.6)	-0.003	5,407 (5.6)	5,330 (5.5)	0.003
2016	6,003 (6.2)	2,113,080 (5.7)	0.021	6,003 (6.2)	6,099 (6.3)	-0.004
2017	6,564 (6.8)	2,164,948 (5.8)	0.039	6,564 (6.8)	6,706 (6.9)	-0.006
2018	7,841 (8.1)	2,215,017 (6)	0.083	7,840 (8.1)	7,975 (8.2)	-0.005
2019	8,045 (8.3)	2,228,358 (6)	0.089	8,044 (8.3)	8,214 (8.5)	-0.006
2020	7,393 (7.6)	2,224,544 (6)	0.065	7,391 (7.6)	7,459 (7.7)	-0.003

*LTRA, leukotriene receptor antagonist; SMD, standardized mean difference; IQR, interquartile range; IMD, Index of Multiple Deprivation; BMI, body mass index.

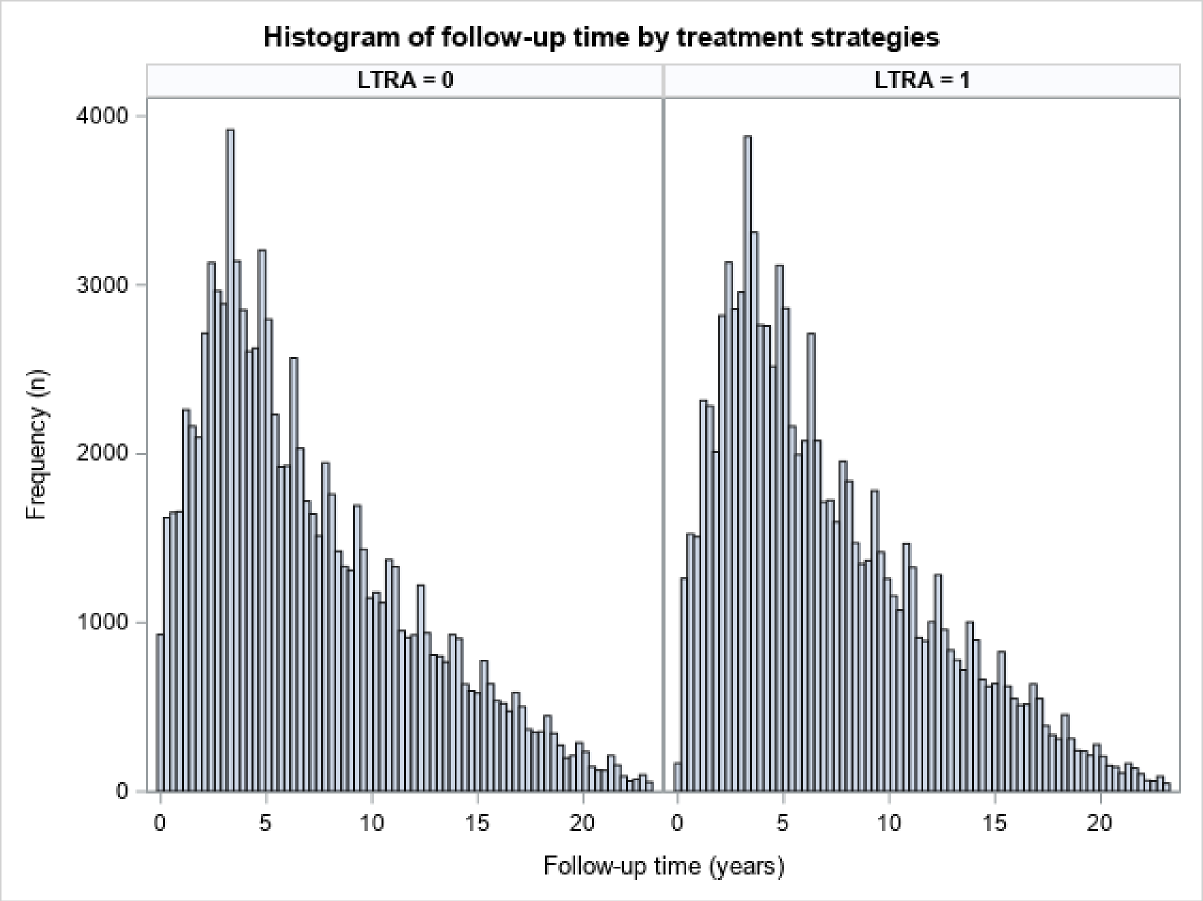

Supplementary Figure 1. Histograms showing the distribution of follow-up time by treatment groups.

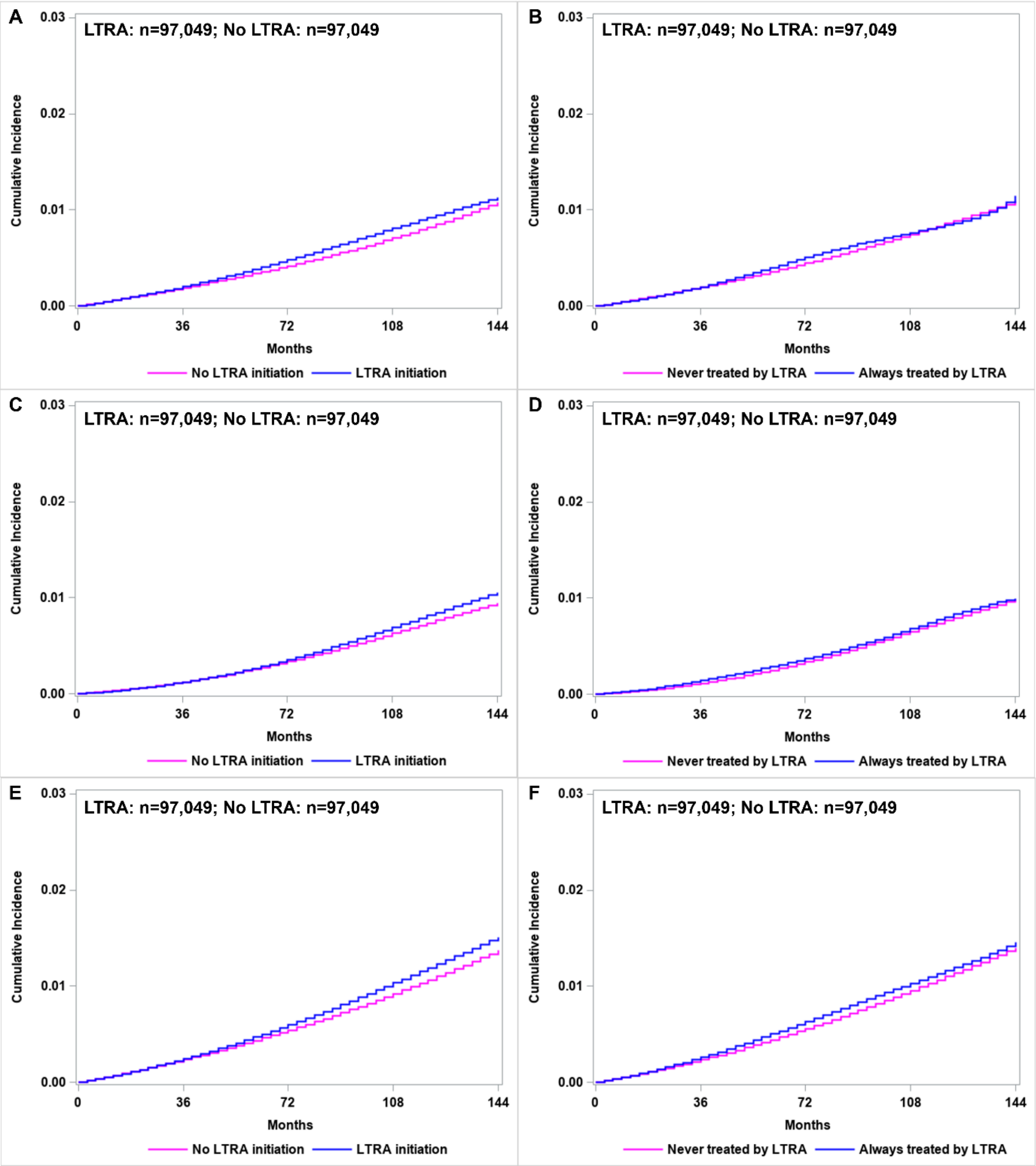

Supplementary Figure 2. Cumulative incidence of Parkinson's disease with alternative definitions with leukotriene receptor antagonist (LTRA) treatment and no LTRA treatment among patients with asthma.

(A) as having a GP record of Parkinson's disease, intention-to-treat effect, 10-year risk ratio 1.12 (95% confidence interval 0.99 to 1.27);
(B) as having a GP record of Parkinson's disease, per protocol effect, 10-year risk ratio 0.98 (95% confidence interval 0.79 to 1.19);
(C) as having a hospital admission due to Parkinson's disease, intention-to-treat effect, 10-year risk ratio 1.11 (95% confidence interval 0.96 to 1.29);
(D) as having a hospital admission due to Parkinson's disease, per protocol effect, 10-year risk ratio 1.05 (95% confidence interval 0.82 to 1.30);

(E) as having a GP record or a hospital admission due to Parkinson's disease, intention-to-treat effect, 10-year risk ratio 1.12 (95% confidence interval 0.99 to 1.25);
(F) as having a GP record or a hospital admission due to Parkinson's disease, per protocol effect, 10-year risk ratio 1.06 (95% confidence interval 0.89 to 1.24).
The number of patients (n) included in each analysis under each treatment group was labelled in each panel. Cumulative incidence curves were constructed using pooled logistic regression models to estimate discrete-time hazards at 3-month intervals. Each data point represents the estimated cumulative risk at the corresponding time point. 95% confidence intervals were calculated using non-parametric bootstrapping with 300 resamplings. No formal test statistic or p-value is reported due to the use of non-parametric bootstrap-based inference.

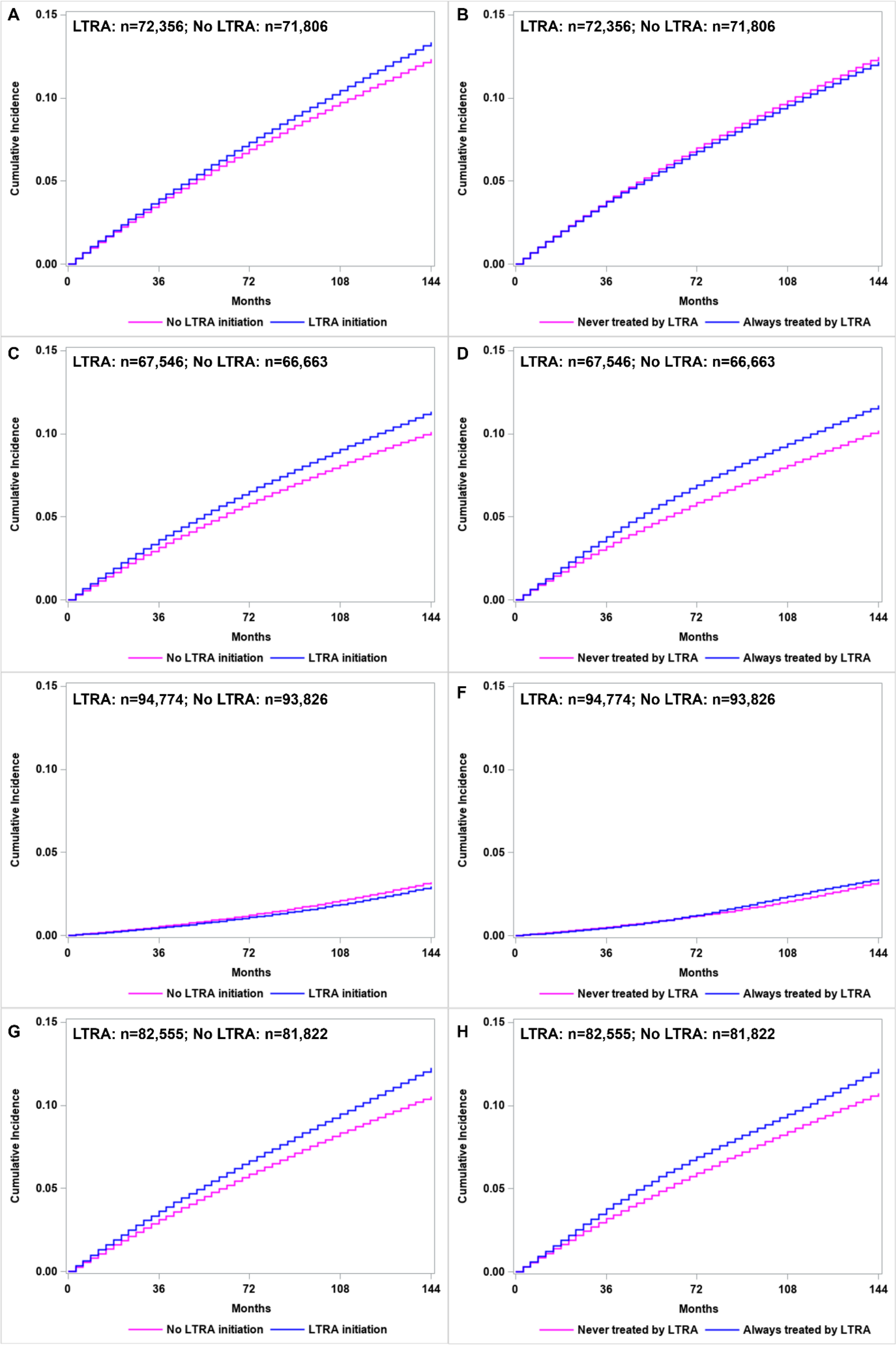
A
LTRA: n=72,356; No LTRA: n=71,806
B
LTRA: n=72,356; No LTRA: n=71,806
C
LTRA: n=67,546; No LTRA: n=66,663
D
LTRA: n=67,546; No LTRA: n=66,663
LTRA: n=94,774; No LTRA: n=93,826
F
LTRA: n=94,774; No LTRA: n=93,826
G
LTRA: n=82,555; No LTRA: n=81,822
H
LTRA: n=82,555; No LTRA: n=81,822
Cumulative Incidence
0.00
0.05
0.10
0.15
0
36
72
108
144
Months
No LTRA initiation
LTRA initiation
Never treated by LTRA
Always treated by LTRA

Supplementary Figure 3. Cumulative incidence of neuropsychiatric events and sleep disorders with leukotriene receptor antagonist (LTRA) treatment and no LTRA treatment among patients with asthma.

(A) anxiety, intention-to-treat effect, 10-year risk ratio 1.08 (95% confidence interval 1.03 to 1.12);
(B) anxiety, per protocol effect, 10-year risk ratio 0.97 (95% confidence interval 0.92 to 1.03);
(C) depression, intention-to-treat effect, 10-year risk ratio 1.12 (95% confidence interval 1.07 to 1.16);
(D) depression, per protocol effect, 10-year risk ratio 1.15 (95% confidence interval 1.08 to 1.22);
(E) psychosis, intention-to-treat effect, 10-year risk ratio 0.90 (95% confidence interval 0.83 to 0.98);
(F) psychosis, per protocol effect, 10-year risk ratio 1.13 (95% confidence interval 0.99 to 1.27);
(G) sleep disorders, intention-to-treat effect, 10-year risk ratio 1.14 (95% confidence interval 1.11 to 1.19);
(H) sleep disorders, per protocol effect, 10-year risk ratio 1.12 (95% confidence interval 1.06 to 1.19).
The number of patients (n) included in each analysis under each treatment group was labelled in each panel. Cumulative incidence curves were constructed using pooled logistic regression models to estimate discrete-time hazards at 3-month intervals. Each data point represents the estimated cumulative risk at the corresponding time point. 95% confidence intervals were calculated using non-parametric bootstrapping with 300 resamplings. No formal test statistic or p-value is reported due to the use of non-parametric bootstrap-based inference.

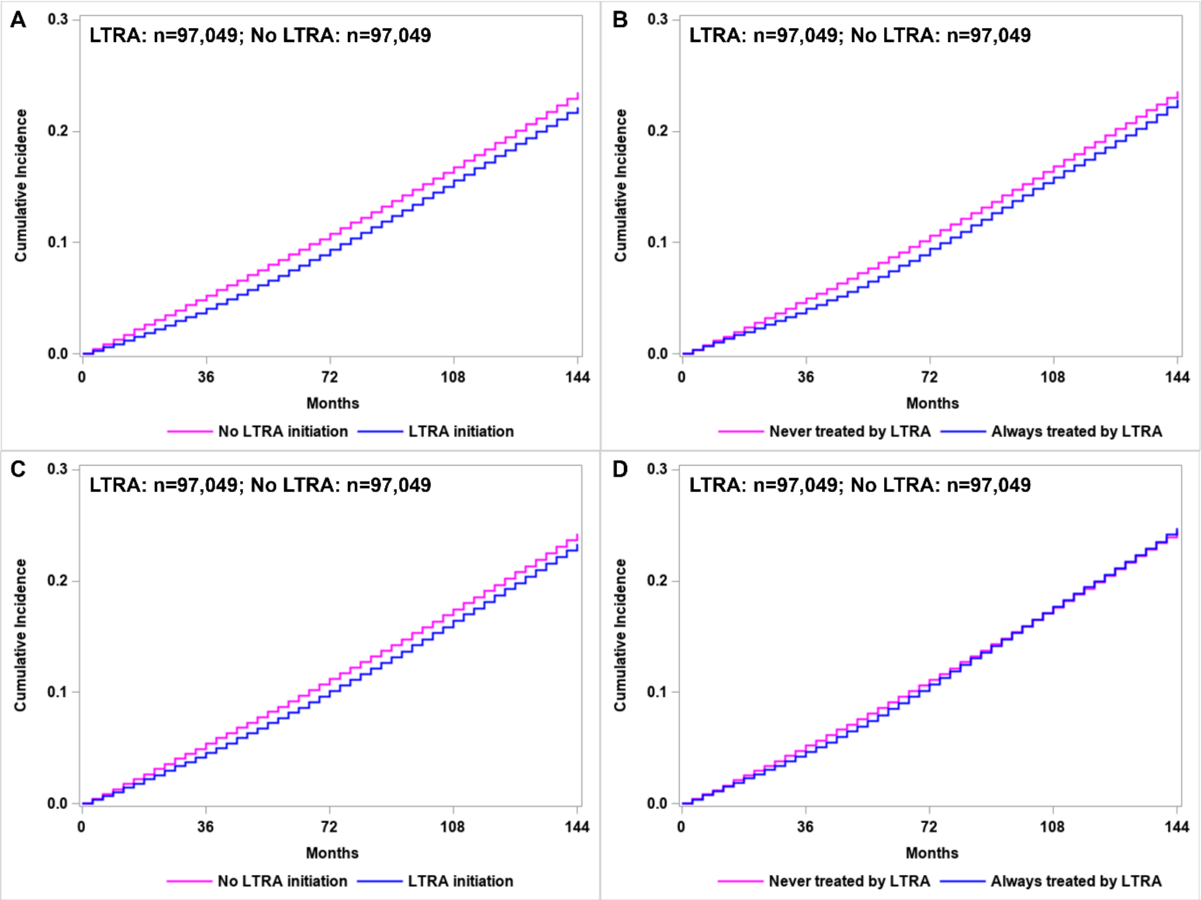

Supplementary Figure 4. Cumulative incidence of mortality with leukotriene receptor antagonist (LTRA) treatment and no LTRA treatment among patients with asthma.

(A) all-cause mortality, intention-to-treat effect, 10-year risk ratio 0.94 (95% confidence interval 0.92 to 0.96);

(B) all-cause mortality, per protocol effect, 10-year risk ratio 0.94 (95% confidence interval 0.91 to 0.98);

(C) composite of all-cause mortality and Parkinson's, intention-to-treat effect, 10-year risk ratio 0.95 (95% confidence interval 0.93 to 0.97);

(D) composite of all-cause mortality and Parkinson's, per protocol effect, 10-year risk ratio 1.00 (95% confidence interval 0.97 to 1.04).

The number of patients (n) included in each analysis under each treatment group was labelled in each panel. Cumulative incidence curves were constructed using pooled logistic regression models to estimate discrete-time hazards at 3-month intervals. Each data point represents the estimated cumulative risk at the corresponding time point. 95% confidence intervals were calculated using non-parametric bootstrapping with 300 resamplings. No formal test statistic or p-value is reported due to the use of non-parametric bootstrap-based inference.

Supplementary Table 5. Sensitivity analysis: point estimates of the absolute risks and risk ratios for Parkinson's disease comparing LTRA treatment versus no LTRA treatment, excluding reinitiators of LTRA and their matched pairs.

		Intention-to-treat				Per protocol with cumulative dose model			
	No. of events/ No. patients	5-year absolute risk (%)	5-year risk ratio	10-year absolute risk (%)	10-year risk ratio	5-year absolute risk (%)	5-year risk ratio	10-year absolute risk	10-year risk ratio
**Primary definition**									
LTRA	489/84,052	0.35	1.06	0.84	1.07	0.35	1.04	0.78	0.99
No LTRA	457/84,052	0.33	Reference	0.78	Reference	0.33	Reference	0.79	Reference
**Parkinson's disease by GP record**									
LTRA	528/84,052	0.37	1.13	0.90	1.13	0.39	1.16	0.87	1.06
No LTRA	480/84,052	0.33	Reference	0.80	Reference	0.33	Reference	0.82	Reference
**Parkinson's disease by hospital admission**									
LTRA	454/84,052	0.25	1.02	0.77	1.07	0.28	1.21	0.75	1.02
No LTRA	426/84,052	0.25	Reference	0.72	Reference	0.23	Reference	0.73	Reference
**Parkinson's disease by GP record or a hospital admission**									
LTRA	678/84,052	0.45	1.08	1.15	1.10	0.49	1.18	1.14	1.08
No LTRA	624/84,052	0.42	Reference	1.04	Reference	0.41	Reference	1.05	Reference

LTRA, leukotriene receptor antagonist; GP, general practice.

Supplementary Table 6. Sensitivity analysis: point estimates of the absolute risks and risk ratios for Parkinson's disease comparing LTRA treatment versus no LTRA treatment, sequential trials by calendar time.

		Intention-to-treat				Per protocol with cumulative dose model			
	**No. of events/ No. patients**	**5-year absolute risk (%)**	**5-year risk ratio**	**10-year absolute risk (%)**	**10-year risk ratio**	**5-year absolute risk (%)**	**5-year risk ratio**	**10-year absolute risk**	**10-year risk ratio**
**Primary definition**									
LTRA	572/95,709	0.36	1.00	0.89	1.07	0.37	1.02	0.78	0.97
No LTRA	532/95,709	0.36	Reference	0.84	Reference	0.36	Reference	0.80	Reference
**Parkinson's disease by GP record**									
LTRA	600/95,709	0.38	1.03	0.90	1.13	0.41	1.12	0.84	0.97
No LTRA	551/95,709	0.37	Reference	0.80	Reference	0.37	Reference	0.86	Reference
**Parkinson's disease by hospital admission**									
LTRA	535/95,709	0.27	1.05	0.83	1.13	0.31	1.20	0.79	1.04
No LTRA	508/95,709	0.26	Reference	0.73	Reference	0.26	Reference	0.76	Reference
**Parkinson's disease by GP record or a hospital admission**									
LTRA	780/95,709	0.47	1.00	1.17	1.10	0.52	1.12	1.12	1.01
No LTRA	731/95,709	0.47	Reference	1.07	Reference	0.44	Reference	1.10	Reference

LTRA, leukotriene receptor antagonist; GP, general practice.

Supplementary Table 7. Sensitivity analysis: point estimates of the absolute risks and risk ratios absolute risks and risk ratios for Parkinson's disease comparing LTRA treatment versus no LTRA treatment, with at least five year record history.

		Intention-to-treat				Per protocol with cumulative dose model			
	No. of events/ No. patients	5-year absolute risk (%)	5-year risk ratio	10-year absolute risk (%)	10-year risk ratio	5-year absolute risk (%)	5-year risk ratio	10-year absolute risk (%)	10-year risk ratio
**Primary definition**									
LTRA	505/ 83,773	0.37	1.11	0.88	1.15	0.38	1.10	0.82	1.04
No LTRA	445/ 83,773	0.34	Reference	0.76	Reference	0.35	Reference	0.79	Reference
**Parkinson's disease by GP record**									
LTRA	537/ 83,773	0.39	1.09	0.92	1.09	0.42	1.13	0.90	1.05
No LTRA	491/ 83,773	0.36	Reference	0.84	Reference	0.37	Reference	0.86	Reference
**Parkinson's disease by hospital admission**									
LTRA	467/ 83,773	0.26	1.04	0.82	1.11	0.29	1.16	0.80	1.05
No LTRA	418/ 83,773	0.25	Reference	0.74	Reference	0.25	Reference	0.77	Reference
**Parkinson's disease by GP record or a hospital admission**									
LTRA	696/ 83,773	0.48	1.02	1.20	1.09	0.50	1.06	1.20	1.06
No LTRA	641/ 83,773	0.47	Reference	1.10	Reference	0.47	Reference	1.13	Reference

LTRA, leukotriene receptor antagonist; GP, general practice.

Supplementary Table 8. Sensitivity analysis: point estimates of the absolute risks and risk ratios for Parkinson's disease comparing LTRA treatment versus no LTRA treatment, with a 3-year lead time.

		**Intention-to-treat**		**Per protocol with cumulative dose model**	
	**No. of events/ No. patients**	**10-year absolute risk (%)**	**10-year risk ratio**	**10-year absolute risk (%)**	**10-year risk ratio**
**Primary definition**					
LTRA	422/95,531	0.71	1.12	0.58	0.88
No LTRA	379/95,534	0.64	Reference	0.65	Reference
**Parkinson's disease by GP record**					
LTRA	461/95,526	0.76	1.13	0.65	0.92
No LTRA	414/95,535	0.67	Reference	0.71	Reference
**Parkinson's disease by hospital admission**					
LTRA	439/95,595	0.72	1.11	0.66	0.96
No LTRA	398/95,596	0.65	Reference	0.69	Reference
**Parkinson's disease by GP record or a hospital admission**					
LTRA	605/95,484	1.00	1.13	0.91	0.97
No LTRA	542/95,494	0.88	Reference	0.93	Reference

LTRA, leukotriene receptor antagonist; GP, general practice.

Supplementary Table 9. Sensitivity analysis: point estimates of the absolute risks and risk ratios for anosmia, constipation, dizziness, and urinary incontinence (autonomic and sensory presentations associated with Parkinson's) comparing LTRA treatment versus no LTRA treatment.

		**Intention-to-treat**				**Per protocol with cumulative dose model**			
	**No. of events/ No. patients**	**5-year absolute risk (%)**	**5-year risk ratio**	**10-year absolute risk (%)**	**10-year risk ratio**	**5-year absolute risk (%)**	**5-year risk ratio**	**10-year absolute risk**	**10-year risk ratio**
**Anosmia**									
LTRA	576/95,709	0.47	1.21	0.78	1.32	0.43	1.13	0.71	1.19
No LTRA	435/95,709	0.39	Reference	0.59	Reference	0.38	Reference	0.59	Reference
**Constipation**									
LTRA	12,989/95,709	9.50	1.05	18.16	1.07	9.50	1.03	18.48	1.07
No LTRA	12,013/95,709	9.03	Reference	16.92	Reference	9.17	Reference	17.23	Reference
**Dizziness**									
LTRA	18,240/95,709	12.90	1.09	26.44	1.07	12.51	1.03	26.22	1.04
No LTRA	16,704/95,709	11.82	Reference	24.65	Reference	12.15	Reference	24.48	Reference
**Hearing loss**									
LTRA	13,504/95,709	9.92	1.04	20.16	1.05	10.29	1.06	21.70	1.10
No LTRA	12,711/95,709	9.56	Reference	19.15	Reference	9.64	Reference	19.63	Reference
**Urinary incontinence**									
LTRA	6,022/95,709	4.36	1.03	8.39	1.07	4.57	1.09	9.43	1.23
No LTRA	5,594/95,709	4.41	Reference	7.80	Reference	4.20	Reference	7.66	Reference

LTRA, leukotriene receptor antagonist.

Supplementary Table 10. Sensitivity analysis: point estimates of the absolute risks and risk ratios for Parkinson's disease comparing LTRA treatment versus no LTRA treatment, stratified by age groups and sex.

		Intention-to-treat					Per protocol with cumulative dose model				
	**No. of events/ No. patients**	**5-year absolute risk (%)**	**5-year risk ratio**	**10-year absolute risk (%)**	**10-year risk ratio**	**p-value for interaction**	**5-year absolute risk (%)**	**5-year risk ratio**	**10-year absolute risk**	**10-year risk ratio**	**P-value for interaction**
**Parkinson's disease (primary definition)**											
>=65 years						0.87					0.26
LTRA	374/40,729	0.83	1.01	2.14	1.13		0.87	1.06	1.91	0.99	
No LTRA	338/40,729	0.82	Reference	1.90	Reference		0.82	Reference	1.94	Reference	
65 years											
LTRA	199/56,320	0.22	1.31	0.59	1.12		0.25	1.47	0.68	1.28	
No LTRA	199/56,320	0.17	Reference	0.53	Reference		0.17	Reference	0.53	Reference	
Male						0.02					0.03
LTRA	285/35,200	0.68	0.98	1.70	1.01		0.73	1.09	1.58	0.92	
No LTRA	298/35,215	0.70	Reference	1.69	Reference		0.67	Reference	1.70	Reference	
Female											
LTRA	288/61,849	0.35	1.22	0.91	1.25		0.37	1.19	0.94	1.23	
No LTRA	239/61,834	0.29	Reference	0.72	Reference		0.31	Reference	0.76	Reference	

LTRA, leukotriene receptor antagonist.
